# The time-varying relationship between economic globalization and the ideological center of gravity of party systems

**DOI:** 10.1371/journal.pone.0212945

**Published:** 2019-02-27

**Authors:** Ingo Rohlfing, Tobias Schafföner

**Affiliations:** 1 Cologne Center for Comparative Politics, University of Cologne, Cologne, Germany; 2 Faculty of Social Sciences, University of Bremen, Bremen, Germany; Universidad de Castilla-La Mancha, SPAIN

## Abstract

Does economic globalization influence the positioning of parties and, as a consequence, the ideological characteristics of party systems? Answering this question is important because we need to understand the constraints that parties face in formulating policies from which voters have to choose. In our paper, we take a *systemic* perspective and conceptualize a party system’s *ideological center of gravity* as the outcome of interest. We define the center of gravity as the weighted mean position of all parliamentary parties in a country that represents the position to which parties gravitate. We start by formulating static hypotheses on the effect of imports and exports on the center of gravity and derive their underlying mechanisms. We further derive dynamic hypotheses stipulating varying effects over time based on the premise that partisan attitudes toward globalization have undergone multiple changes over the last decades. A time-series cross-section analysis of 129 elections in 15 Western European countries from 1974 to 2015 finds evidence for opposite effects of exports and imports in the pooled data. Additionally, a moving-window analysis indicates that the relationship between globalization and the center of gravity varies over time. This is a significant finding because it suggests that economic globalization has an influence on party systems and that it is important to test for time-varying effects.

## 1 Introduction

Political parties made economic globalization possible by reducing barriers to the free flow of goods and services [1]. However, did its promotion backfire by influencing the ways in which parties position themselves on national and international economic issues and, as a consequence, the ideological characteristics of party systems? There is extensive research on how globalization is related to political, social and economic output and outcomes and party positioning [2–4], but there have been relatively few studies on its relationship with party systems. Research on globalization effects on party positioning yields contradictory evidence. There is evidence of an "efficiency effect" and rightward shifts of parties [5], probably moderated by the median voter [6], and for a "compensation effect" and leftward shifts of party positions [7, 8]. These studies present valuable insights, but they do not offer information on the *systemic effects* of globalization because of the fallacies of inferring effects on party systems from globalization effects on individual parties. The effects of economic globalization on party systems work through the positioning of individual parties but, to study these effects, the empirical analysis must center on party systems and cannot be inferred from a party-level analysis. An analysis of the relationship between globalization, party positioning and their implications for the characteristics of party systems is of societal importance. Economic globalization could affect party positioning and, by implication, the ideological characteristics of party systems and policies offered to voters on a systemic level. Depending on how globalization affects party systems, it might indirectly and negatively shape citizen satisfaction with those parties and with democracy as a whole [9].

In our paper, we take a *systemic* perspective, with a party system’s *ideological center of gravity* as the outcome of interest. We define the center of gravity as the weighted mean position of all parliamentary parties in a country. In section 2, we introduce the concept of the ideological center of gravity and demonstrate that it is conceptually different from other measures commonly used in research on party systems. In section 3, we explain the way in which we decompose ‘economic globalization’ into flows of imports and exports because the two dimensions create different incentives for the positions taken by political parties. Initially, we formulate static hypotheses on the effect of each globalization dimension on the center of gravity. We continue by arguing that it is of crucial importance to take a *dynamic* perspective and trace globalization effects over time, which, to our knowledge, has not yet been done in the party politics literature on globalization. The rationale for a dynamic view is that the attitudes of parties and the public toward globalization have, on multiple occasions, undergone massive changes over the last decades, making it unlikely that there is a uniform effect of economic globalization over time. We develop three hypotheses on how globalization affected the center of gravity between the 1970s and early 2010s, which is our period of analysis.

We present our empirical strategy and data in section 4. In section 5, we discuss the results of a time-series cross-section regression involving 129 elections from 15 Western European democracies from 1974 to 2015. Our analysis includes the recent wave of globalization and goes beyond previous studies that excluded the latest Euro crisis. The results point to the advantages of supplementing a pooled, static analysis with a dynamic perspective. The results for the pooled analysis fail to reject the null hypotheses of no effects for the two globalization dimensions. This finding is in accord with previous research that, at best, found mixed results for globalization effects on party positioning. In contrast, the disaggregated analysis supports our argument that the association between the globalization dimensions and party systems changes over time and includes significant and non-significant estimates of varying size. We discuss the implications of our results in section 7 and make concluding remarks and discuss our study’s limitations in section 8.

## 2 The ideological center of gravity of party systems

Our analysis focuses on party systems and their ideological center of gravity (henceforth COG). Following conventional usage, we use ‘party ideology’ synonymously with a party’s position on a dimension of party competition, which is the economic dimension in our paper. That position depends on how the party positions itself on the issues or policies that constitute the dimension [10]. Gross and Sigelman [11] introduced the COG as one element of party systems that has received scant attention compared to other measures such as fractionalization and polarization [12]. We define the COG as the mean positioning of parties on a specific dimension, with party positions weighted by their vote shares. We use the vote share instead of the parliamentary seat share to avoid distorting effects deriving from the disproportionality of the electoral system. At the heart of our analysis is the *economic* dimension of party competition because this is where we expect to observe the consequences of economic globalization. The COG conveys information about the hypothetical point on the economic dimension to which parties gravitate. The COG is an important measure because it indicates whether parties take positions such that the center is located more on the left, right or middle of the economic dimension and the hypothetical point on the dimension from which voters can choose among parties. We are aware of the argument that economic globalization made parties more responsive on a non-economic or social dimension because of globalization constraints on their behavior on the economic dimension [6, 13]. If this argument was true, there might still be an effect of globalization on the COG on an economic dimension. The argument about the decreasing room of maneuver is tied to the claim that party positions converge in a specific range of the economic dimension and the COG should shift in response to globalization. Globalization effects on party positioning on a social dimension, which are not our theoretical interest, are therefore compatible with an effect of globalization on the COG.

Conceptually, the COG is distinct from polarization as an alternative and more widely studied metric of party systems and the convergence and divergence of party positions [8, 14]. Polarization measures the spread of party positions and the range of positions from which voters can choose. The convergence or divergence of party positions captures whether the diversity of party positions becomes broader or more narrow over time. Polarization and convergence/divergence are important areas of study in their own right, but they are empirically uninformative as to where the COG is located on the economic dimension. It is conceivable that economic globalization is unrelated to polarization and convergence of party positions, but causes parties to move, on average, to the right or left of the economic dimension, thus affecting the policies from which voters can choose. In this perspective, the empirical analysis of the COG is a complement to the study of polarization and convergence and necessary to developing a more complete picture about the relationship between economic globalization and party systems.

## 3 Formulation of hypotheses

### 3.1 Economic globalization and the center of gravity: Static perspective

We define *economic globalization* as the transborder flow of goods and investments and conceptualize it as a multidimensional phenomenon [2]. These trade and capital flows are facilitated by low tariff and non-tariff barriers. We take a disaggregated perspective based on the premise that there is no uniform globalization effect because different components of economic globalization have different consequences for party systems. Our analysis centers on the consequences of the flow of imports and exports. Compared to capital flows, trade flows are the most apparent component of economic globalization and lead to greater scrutiny by voters and parties [15]. The decision is supported by existing research finding no significant effect of foreign direct investment (FDI) on party positioning [8]. Below, we will introduce capital flows in the form of FDI as a robustness test. For the outcome, we focus on the economic dimension because of empirical evidence that the policy space is not well-described by a single left-right dimension and that an economic dimension is central to party competition in virtually all Western European countries ([16], chapters 5, 6).

The sum of total trade relative to the economic output is regularly used as a measure of economic globalization (KOF index [17]). In contrast, we argue and then test the assumption that it is necessary to decompose trade into imports and exports because they have different economic consequences for countries. These economic differences need to be examined and understood because political parties holding different positions toward the national economy respond differently to these national economic effects of globalization. We argue that the actual responses of parties to this incentive differ due to the initially dissimilar positions that parties occupy on the economic dimension. In the following discussion, we adhere to the convention that smaller values on a dimension, as well as a negative correlation between a covariate and the center of gravity, represent more leftish positions and changes.

The central premise of our hypotheses is that parties perceive imports negatively and exports positively. The difference in the perceptions of imports and exports can be explained in three ways, the first being derived from a standard political economy model of political support of organized groups [18, 19], which has previously been effectively applied to tariff making and trade cooperation [20, 21]. Higher imports increase competitive pressure on domestic companies producing goods that compete with imported goods (henceforth, “import-competers”). At the same time, they benefit domestic producers who require imports to serve as inputs to their production process (henceforth, “input-users”) and consumers by lowering the prices of the good. The unilateral reduction of tariffs might be welfare-enhancing, but it creates political backlash because of the organized opposition of import-competers. In contrast, input-users support higher import levels because it is to their advantage. Exporters, the third organized group that is relevant here, are also interested in rising import levels in their home countries. They follow the idea of reciprocity to leverage enhanced domestic market access for easier foreign access and, thus, rising imports at home and abroad.

We argue that parties confronting this constellation of domestic groups target those groups’ interests with specific policy proposals. The negative perception of imports creates an incentive for sheltering import-competers from imports by creating non-tariff barriers to trade as well as supporting measures such as subsidies. A study by Kono [22] corroborates this reasoning by showing that reduced tariff levels coincided with an increase in less visible non-tariff barriers to trade. An example of the use of subsidies to reduce import competition is the agricultural sector in many developed countries. Parties from both the left and right supported subsidizing agricultural producers for an extended period of time to shelter them from import competition, at least to some degree. In the 1980s, for example, the European Community and the United States were waging a “subsidy war” in the wheat sector that was costly to both sides and came at the expense of exporters from third countries ([23] p. 153). With regard to the consequences for party position taking, we argue that the incentive to favor protectionist leftist policies is stronger, the more parties are positioned to the right of the spectrum because parties on the left are already endorsing the idea of sheltering national producers. Import levels should therefore be negatively correlated with the COG.

The second mechanism works via the expectations of voters who feel more exposed to the pressure of rising imports. Voters with jobs that are vulnerable to rising imports expect parties to diminish their vulnerability, for example, by spending more on job training programs, etc. or by increasing spending (compensation hypothesis [24]). A counterargument is that left parties might also move more to the right of the economic dimension, reflecting their belief that spending levels cannot be maintained under conditions of economic globalization (efficiency hypothesis [25]). Our reasoning on the incentives for party positioning that work via voters does not require that welfare spending or spending more generally increases, which is the traditional focus of the compensation hypothesis. Instead, we argue that parties have incentives to offer policies that shelter voters from import competition, which might work in the same way as for import-competers, i.e., by pursuing statist industrial policies such as granting subsidies to industries. Trade policy making of the current Trump administration is an example of the protection of workers in import-competing sectors without financial compensation or increased welfare spending. The steel sector, which faces strong competition, is simply protected by increased tariff levels that reduce the level of imports and (supposedly) save jobs in the US steel industry. Following this argument, parties that are located on the left have already assumed a position that is in accord with the demand for higher compensation, whatever form it might take. The further a party is located to the right, the stronger the incentive to adjust its position and make a move to the left to compete for compensation-demanding voters.

A third possible mechanism is a mercantilist understanding of trade policy making. Mercantilism is long outdated among economists [26], but one can observe mercantilist thinking over the past 200 years until the present. Countries that import more than they export run current account deficits, which are traditionally considered to be undesirable because they are thought to be a sign of economic weakness [15]. Current account deficits and surpluses have been a salient issue since the beginning of modern trade relations in the 19^th^ century [27] and were also central to the recent Euro crisis. Germany has been running an account surplus since the mid-2000s and has lauded this as a sign of Germany’s economic strength and a role model for struggling countries such as Greece and Portugal [28]. This reasoning reflects the view that export surpluses equal strength and that high levels of imports are bad for an economy (which does not make sense in economic terms, but that is another matter). The policy implications of mercantilism are to make import-competers more competitive because the stronger they are, the less goods will be imported.

We argue that the mechanisms should hold in times of increasing international division of labor and a shift from inter-industry to intra-industry trade. If one perceives imports as an indication of domestic economic weakness or as a threat to the support of import-competers, it does not matter whether goods are traded on an inter-industry or intra-industry level. For example, this is reflected in the mercantilist move by the current Trump administration, the arguments of pro-Brexit groups who wanted to retake control over trade relations and imports and the support of German car manufacturers by the German government, that repeatedly and with different partisan composition prevented the regulation of emissions in the EU Council of Ministers.

*Hypothesis 1: Increasing levels of imports are associated with a leftward shift of the center of gravity and decreasing levels of imports with a rightward shift on the economic dimension of party competition*.

In contrast to imports, we find that *exports* are welcomed by all parties because they benefit exporters, who increase their support for parties strengthening the exporter’s competitiveness [29]. Workers employed by exporters also benefit from higher exports because that secures their jobs and increases their income. The agricultural industry is a case in point for these arguments because, for decades, developed countries have implemented export promotion programs to support their producers ([30], p. 116). From a mercantilist perspective, exports are desirable simply because they contribute to a current account surplus.

These points at least hold for developed economies that export manufactured products and services, although it might differ for developing countries predominantly exporting primary goods or those that export rare goods such as the minerals needed in the telecommunications industry. However, our theoretical arguments are confined to developed economies. Exports have been interpreted this way from the inception of the trade doctrine of mercantilism until the present day [15], including the Euro crisis wherein the ailing states were blamed for a lack of economic competitiveness and weak export industries. We assume that parties agree on the need to support exporters via appropriate policies, whereas the exact policies that are favored depend on whether a party is positioned on the left or right side of the political spectrum. Following theories on partisan ideology and economic policy making [31, 32], we argue that parties located more to the right of the spectrum endorse supply-side policies such as lower corporate taxes and deregulation of the labor market. Parties on the left embrace demand-side policies that are unsuitable for the support for exporters. Instead, they seek to shelter domestic producers from import competition via tariff and non-tariff barriers because of their belief that less competition at home means enhanced competitiveness abroad [33]. Another means by which parties can promote exports are subsidies, which are also an instrument of the political left, either in the form of export subsidies or subsidies specifically targeted at domestic producers [34]. In contrast to lower taxes and less regulation, subsidies represent an intervention of the state into the market and are not an element of supply-side politics.

Following this reasoning, leftist and rightist parties are already located on the proper sides of the spectrum, “proper” meaning that the current position is largely in line with the incentives created by exports. When exports increase, we expect parties located on the left to either remain stationary or move more to the left, on average, while parties on the right either stay put or shift further to the right. We therefore hypothesize that the rightward shifts of rightist parties and leftward shifts of leftist parties cancel each other out, with the consequence that the ideological center of gravity remains in place.

*Hypothesis 2: Changes in the level of exports are unrelated with a party system’s center of gravity on the economic dimension of party competition*.

We do not argue that all parties held uniform views on economic globalization during these periods (the same holds for the hypotheses we develop in the following section). However, we believe that it is possible to develop arguments applicable to many, in particular, the major European parties that have more weight for a party system’s COG.

### 3.2 Economic globalization and the center of gravity: Dynamic perspective

Economic globalization has been a contested issue among the public, politicians and organized groups since the beginning of the modern globalization era in the mid-19^th^ century [15]. We expect the two static hypotheses to hold in an aggregate analysis pooling the data from multiple decades because these should be, on average, a relationship between imports, exports and the COG. However, we also believe that it is important to take a dynamic perspective based on the assumption that the parties’ view on and responses to imports and exports have changed over time. One could choose any four decades from 1860, which is usually seen as the starting point of modern trade relations [35], and would observe at least one major change in economic globalization and how countries perceived and responded to it (see for example, [15]).

This general empirical insight motivates the formulation of multiple dynamic hypotheses for our period of analysis, which ranges from 1974 to 2015. We divide it into three stages according to our expectation of Western European parties’ responses to imports and exports. The expectations and hypotheses are derived from existing research on partisan behavior during these four decades and our own perspective on the empirical developments. We are unable to precisely determine the breakpoints between the three periods because policy beliefs seamlessly supersede one another. However, we think that it is possible to determine the approximate beginning and end of a stage. The empirical analysis of the dynamic hypotheses is a moving-window analysis that does not require a sharp differentiation between the three periods.

The first stage ranges from the 1960s to the mid-1980s. This period is characterized by the economic problems of the 1960s and 1970s, which became manifest in the budget and currency problems of the United States [36], the end of the Bretton Woods system, the two oil crises and the economic stagnation combined with high inflation that existed until the early 1980s. Keynesian demand policies, which are inward-oriented and seek to stimulate domestic demand via public deficit spending, prevailed during the 1970s among left parties [37]. At the same time, parties on the right of the spectrum started leaning toward monetarist policies. The Reagan administration initiated the Tax Reform Act of 1986, which set the precedent for cutting the top marginal income tax rates in many Western European countries [38]. On the international level, it would be appropriate to include the GATT Tokyo Round, but that only achieved moderate progress because states were cautious about pushing forward liberalization in difficult economic times [39]. Taken together, we argue that the positions of leftist and rightist parties in the 1970s and early 1980s were driven by the motivations that we previously laid out for the static hypotheses.

*Hypothesis 3: Levels of exports are not associated with the center of gravity between the 1970s and the mid-1980s*.

Imports were seen negatively by all parties in difficult economic times, who sought to counter them with leftist, protectionist measures and account for a negative association between imports and the COG. For example, Western countries used their superior political and economic power to protect their textiles industry against the upcoming competition of developing countries by negotiating import quotas and voluntary export restraints for textile exports [40].

*Hypothesis 4: Levels of imports are negatively associated with the center of gravity between the 1970s and the mid-1980s*.

The late 1980s and 1990s represented a turning point because left parties, social-democratic and socialist parties in particular, started embracing monetarist and supply-side policies [41]. One can date the peak of this period of moderation to the late 1990s and early 2000s ([42], p. 801), when leftist governments in Europe pursued “The Third Way”, lowered corporate taxes and engaged in cutbacks of the state in a manner not previously conceivable. For example, in a series of reforms, Labour raised the bar for the receipt of welfare state benefits to increase pressure on the unemployed to find a job ([43], p. 84). Regardless of party ideology, parties endorsed the idea of privatizing state-owned monopolists in a wide range of sectors, including aviation, postal services and telecommunication [44, 45].

Because parties on the left and right endorsed supply-side policies, liberalization and increased economic globalization during this period, we expect that the link between imports and exports and the COG was most pronounced between the mid-1980s and early 2000s. Parties on the right also reflected a changed attitude towards globalization by taking more radical positions and pushing for stronger retrenchment of the state and lower taxes than did the left parties [46]. For the period between the mid-1980s and early 2000s, we hypothesize that a positive association between exports and the COG existed because both left parties and right parties pursued supply-side policies with the goal of stimulating them.

*Hypothesis 5: Between the mid-1980s and the early 2000s, the association between levels of exports and the center of gravity is positive*.

With regard to imports, we expect that the negative association is stronger in the second period than in the first. A strengthening negative effect is a direct consequence of economic liberalization and globalization because it stimulated exports and economic integration. Since trade liberalization is usually reciprocal, countries have to buy an increase in exports by accepting higher levels of imports [33]. Import-competers feel increased pressure from rising imports and voters become increasingly vulnerable to tougher competition, making them susceptible to more radical parties that propose protectionist policies (economic nationalism, [47, 48]). We argue that left and right parties did not become blind to producers and voters exposed to import competition and endorse policies that at least partially compensate for the increased exposure that parties expected to follow due to import competition [24]. As Kono [22] shows, measures of protection and support that are less visible than tariffs increase when tariffs decrease. Tariff cuts and higher levels of imports more generally therefore do not automatically imply that parties stand by and watch when competition increases for import-competers. Two examples for a demand-side policy that is aimed at workers that falls right in the high days of economic integration and liberalization in the second period is the introduction of a minimum wage in the United Kingdom (1999) and Ireland (2000) [49]. We therefore theorize that higher imports shift the COG to the left in the second period and that the effect is stronger than in the first period.

*Hypothesis 6: Between the mid-1980s and the early 2000s, the association between levels of imports and the center of gravity is more negative than in the first period*.

The stock market crash and economic downturn of the early 2000s constrained national budgets and marked a turning point in attitudes toward economic liberalization among the public and parties. On the international level, the adverse economic development and demise of liberalization were foreshadowed by public protests against a continuation of economic globalization, for example, in Genoa in 2001, and the failure to successfully conclude the so-called Doha Round [50]. The comparatively low-interest rate policies of central banks aimed at supporting economic growth in Western countries then contributed to the second stock market crash in the late 2000s [51], which lead to increased skepticism to economic liberalization and deregulation. We argue that, during that decade, leftist parties reversed their attitudes and turned again to demand-side policies and away from supply-side policies ([52], chapter 6). Parties in the right spectrum could ignore neither the changed economic environment nor public attitudes toward globalization and we expect that they had to moderate their position as well. Take the case of Denmark, for example: In November 2001, the right-wing liberal Venstre party won the general election, formed a minority government with the Conservative People’s Party and governed until 2011. Under two prime ministers, Anders Fogh Rasmussen and Lars Løkke Rasmussen, there were no significant reforms of the welfare state, and their public spending exceeded that of the Social Democratic previous government [53].

We expect the financial crisis that started in 2007 further affects the COG in three ways that are specific to this period. First, the need to bail out banks to avoid bankruptcy meant that countries nationalized or subsidized them ([52], chapter 6), which are traditionally left issues. This was a quick response of parties regardless of their ideology, which we believe to be reflected in the positions of parties in the late 2000s and early 2010s to some degree. Altogether, this should move all parties more to the left. Second, in the medium run, the financial crisis spurred attempts to more tightly re-regulate the financial sector and at least partially reverse the liberalization measures taken in the 1990s and early 2000s. Third, the financial crisis of 2007, bailouts of banks and austerity policies in some countries contributed to the formation of new and strengthening of existing populist parties on the left and right of the spectrum. Left-wing and right-wing populist parties differ in many respects, but have in common that they favor economic nationalism. A key element of economic nationalism is to reduce imports by raising barriers to trade as classic left policy positions [54]. While economically nationalist parties on the left and right have protectionism and economic radicalism in common [55], we expect them to differ in their attitudes toward exports. Exports are compatible with economic nationalism because it is not equivalent with autarchy and the left-right differences between left-wing and right-wing parties should matter here in the ways we have theorized.

Because of these developments, we argue for the third period that the relationship between imports, exports and the COG is again becoming more similar to the relationship in the first period. Existing, non-populist parties were confronted with the consequences of the financial crises of the early 2000s and 2007, seen as a result of the liberalization progress in the previous decade, and returned to what could be called their traditional policies. This development is complemented by the formation and rise of economically nationalist parties that seek protectionist measures against imports.

*Hypothesis 7: Since the early 2000s, the association between levels of exports and imports and the center of gravity is positive and attenuating compared to the second period*.

Table 1 summarizes the expected effects of exports and imports on the ideological center of gravity of party systems.

**Table 1:** Hypotheses—Expected effects of globalization variables on the COG.

	Exports	Imports
**Pooled effect**	**o**	**−**
**Time-variant effects**		
1970s—mid 1980s	**o**	**−**
mid 1980s—early 2000s	**+ +**	**− −**
2000s	**+**	**−**

## 4 Data and empirical strategy

### 4.1 Dependent variable

The dependent variable is derived from the Manifesto Project Database (MARPOR) data [56]. MARPOR is based on human codings of party platforms and assigns quasi-sentences to fifty-six policy categories, plus one category for non-assignable quasi-sentences. These are divided by the number of all quasi-sentences to measure the percentage of all quasi-sentences for each category. We use the percentages to estimate the party position on an economic dimension. In Table 2, we compare existing constructions of the economic dimension of party competition [57–61].

**Table 2:** Comparison of economic dimension of party competition variables.

		Laver/ Budge	Benoit/ Laver	Tavits	Bakker/ Hobolt	Prosser	max
**Right Categories**							
401	Free Market Economy	x	x	x	x	x	**x**
402	Incentives: Positive	x	x	x	x		**x**
407	Protectionism: Negative	x	x	x	x	x	**x**
410	Economic Growth: Positive				x		**x**
414	Economic Orthodoxy	x	x	x	x	x	**x**
505	Welfare State Limitation	x	x		x	x	**x**
507	Education Limitation				x	x	**x**
702	Labour Groups: Negative				x	x	**x**
**Left Categories**							
403	Market Regulation	x	x	x	x	x	**x**
404	Economic Planning	x	x	x	x		**x**
405	Corporatism / Mixed Economy			x	x		**x**
406	Protectionism: Positive	x	x	x	x		**x**
409	Keynesian Demand Management				x		**x**
411	Technology and Infrastructure: Positive					x	**x**
412	Controlled Economy	x	x	x	x	x	**x**
413	Nationalisation	x	x		x	x	**x**
415	Marxist Analysis				x		**x**
503	Equality: Positive				x	x	**x**
504	Welfare State Expansion		x		x	x	**x**
506	Education Expansion		x		x	x	**x**
701	Labour Groups: Positive		x		x	x	**x**

The comparison shows a large intersecting set, with all categories being used belonging to MARPOR domains four, five and seven. Not one of the 21 categories that is coded right by one approach is coded as left by another, or vice versa. However, the different choices of categories for the measurement of party positions reflect that theory does not dictate the choice of one specific set of items. Because of this uncertainty, we decided to follow a maximum approach toward the measurement of party positions on an economic dimension. The maximum approach builds the union of all 21 categories that have been used in one of the five studies to measure party positions. Conceptually, we find this approach defensible because each of the 21 categories can be plausibly linked to the economic dimension of party competition.

We follow customary practice and derive a position estimate by subtracting left from right percentages. A drawback of building the difference is that the estimate is not “independent of irrelevant alternatives” because changes in the number of quasi-sentences not feeding directly into the economic position estimate might affect that estimate ([61], p. 91). We address this problem and standardize the difference between right and left *number* by dividing it with the sum of the right and left percentages: (R-L)/(R+L) [62].

A simple example illustrates the advantage of the ratio measure: For a party manifesto that only consists of 60 right and 20 left quasi-sentences, the right and left percentages are 75 and 25 and the position estimate 50 on a scale from -100 to +100. The standardized measure is 0.5 on a scale ranging from -1 to +1. Now imagine the party adds 20 new quasi-sentences that are neither right nor left on the economic dimension. The non-adjusted difference is 40 (60–20), meaning the party would be estimated to be slightly more centrist, although neither the right nor left content of the manifesto changed. In contrast, the ratio is unaffected by the 20 new sentences because their number remains constant and we again estimate a position at 0.5. We decided against a logit transformation of right and left quasi-sentences [63]. Our procedure takes the same problem into account as the logit transformation, but we find it more intuitive. Because of the importance of the economic dimension and the relatively high number of quasi-sentences underlying it, there is also no need to smoothen estimates to address low numbers of quasi-sentences.

Under certain circumstances, the use of the ratio measure can generate undesired results: The fewer the number of included items is, the easier it can lead to very polarized scales. A manifesto with one or only few right issues and no left issues receives the extreme score of +1. To take that scenario into account it is reasonable to include the highest number of issues that are clearly attributable to either left or right economic policy positions. We therefore continue with our “broad” definition of the economic party positions. Table 3 shows the correlations of equally-constructed economic left-right measures that are based on the different selections of categories shown in Table 2. The strength of the correlations ranges from moderate to very high levels. In a robustness analysis we report below, we test whether the empirical results depend on the choice of measurement approach.

**Table 3:** Correlation matrix for different ratio measures.

	**max**	Laver/ Budge	Benoit/ Laver	Tavits	Bakker/ Hobolt	Prosser
max	**1**					
Laver/Budge	**0,4543**	1				
Benoit/Laver	**0,8989**	0,5378	1			
Tavits	**0,4825**	0,9658	0,5526	1		
Bakker/Hobolt	**0,9711**	0,4719	0,9173	0,4991	1	
Prosser	**0,8800**	0,4163	0,8584	0,4365	0,8132	1

**Fig 1:**
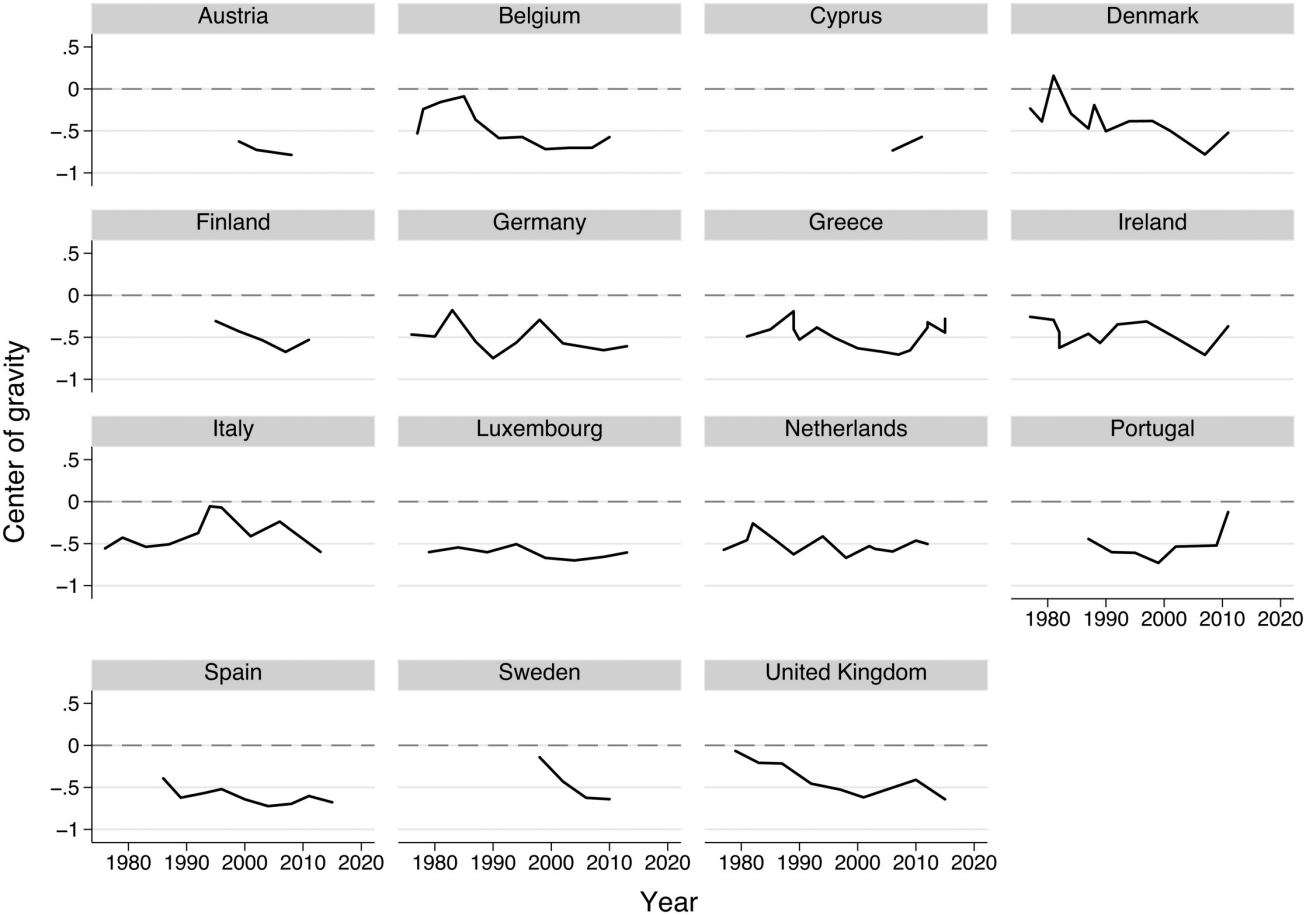
Center of gravity for 15 countries for our period of analysis.

We calculate the COG of a country at time *t* by weighting the position of each parliamentary party on the economic dimension, derived with the maximum approach, with its vote share and taking the sum over the weighted positions. The range of the center lies between -1 and +1 from left to right. Fig 1 presents the development of the COG for the 15 countries and period of analysis that we cover with our study. The figure demonstrates that, both within and between countries, the COG fluctuates over time, which is the variation we seek to exploit. The mean COG across all observations is -0.48, which partially reflects that the economic dimension includes more left than right categories. The line plot might overstate the number of observations per country, which is equal to or less than nine for nine countries out of fifteen. Detrending the imports and exports variables is therefore not warranted [64].

### 4.2 Main covariates

We operationalize ‘economic globalization’ via measures of capital and trade flows and use data extracted from the World Bank’s “World Development Indicators” [65]. We measure the export and import dimension with exports and imports of goods and services standardized with the gross domestic product (GDP) of a country. We lag the globalization variables by one year to account for a temporal lag of the potential effects.

### 4.3 Additional covariates

We add a public opinion measure as a control variable because parties have been found to be responsive to public opinion and the median voter [66], which might have follow-up effects for the COG. We calculate the mean self-reported left-right position of respondents in the Eurobarometer as a measure for the mean voter position. Empirical studies show that the mean voter and mean citizen position are almost perfectly correlated [67], allowing their synonymous use. Since there is no time-series cross-section data allowing us to calculate the mean voter position on the economic dimension, we must rely on public opinion data that locate respondents on the main left-right dimension. The economic dimension and main dimension are strongly correlated in some countries and only weakly in others ([16]), chapter 4, 5], meaning that the public opinion variable is likely to contain measurement error. The public opinion variable is lagged by one year. If more than one Eurobarometer wave was available per year, we pooled data from multiple waves before calculating the mean voter position. We use GDP growth as a control for a country’s economic development. The willingness to implement supply-side reforms increases, the worse the economic situation. GDP per capita controls for the *level* of economic development because more developed countries might have a more leftist center due to an increased focus on postmaterialist values in the electorate as opposed to materialism [68]. All control variables are lagged by one year.

In total, our data comprises 129 observations from elections in 15 countries from 1974 to 2015. We aimed to maximize the spatio-temporal coverage of our data with a focus on Europe to have sufficient power for the moving-window analysis. The main data constraint derives from the availability of the economic variables and the Eurobarometer data. An overview of the variables, operationalization and sources as well as a list of included elections is included in the supporting information (see S1 and S2 Tables as well as S1 Fig). Descriptive statistics for key and control variables are shown in Table 4. See S7 Table for descriptive statistics of the KOF indices that we use below.

**Table 4:** Descriptive statistics.

	Mean	Standard Deviation	Minimum	Maximum	N
**Economic COG**	**-0.4847**	**0.1811**	**-0.7856**	**0.1574**	**129**
Exports (% GDP)	43.56	29.53	14.43	189.24	129
Imports (% GDP)	42.18	23.70	16.89	159.36	129
Public Opinion	5.3428	0.4137	4.3883	6.3902	129
GDP growth (%)	2.0877	2.7025	-9.1325	9.2690	129
GDP/capita (thsd USD)	24,066	17,523	2,923	112,852	129
Trade Balance (% GDP)	1.3749	7.7344	-14.2083	30.3527	129
FDI net inflows (% GDP)	4.8279	14.2971	-3.6792	142.2570	116

### 4.4 Empirical strategy

We first focus on the time-invariant hypotheses with ordinary least-squares regression (OLS). A test for first-order serial autocorrelation rejects the null of no autocorrelation. A test for second-order autocorrelation fails to reject the null. Two F-tests for country fixed-effects and year fixed- effects fail to reject the null of no fixed effects. Based on the diagnostics, we decided to estimate an OLS regression with a lagged dependent variable and standard errors clustered by countries and years. The test for first-order serial autocorrelation when the lagged dependent variable is included fails to reject the null hypothesis of no autocorrelation. Although the F-tests indicate that there is no unobserved heterogeneity between or within countries, we opted for the two-way clustering of standard errors because the panel structure of the data makes it plausible to argue that errors are correlated on both dimensions [69]. Clustered standard errors have been shown to be superior to fixed effects when the unobserved effect varies instead of being constant, which we find plausible in an analysis covering four decades [70]. The following analysis is completely documented in a Stata log-file (S1 File).

## 5 Analysis of time-invariant hypotheses

**Fig 2:**
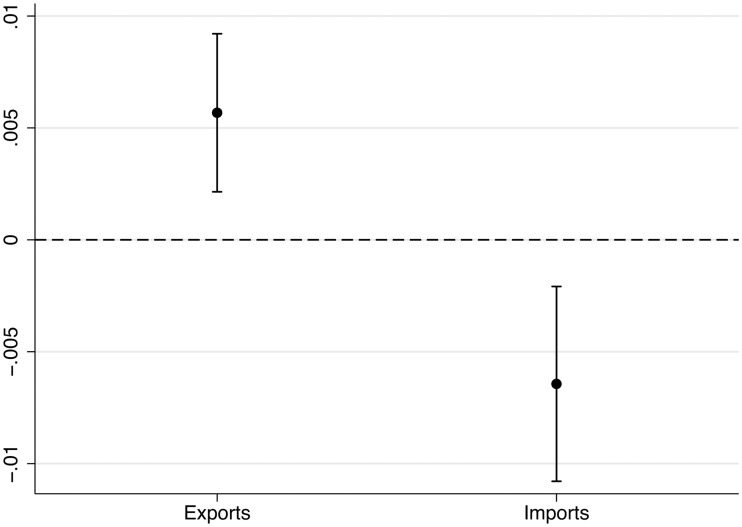
Marginal effect and 95% confidence interval for key independent variables. *n* = 129; OLS model with two-way clustered standard errors and lagged DV.

We present the estimates for imports and exports in Fig 2 and present the full regression results in Table 5, column 1. The estimate for imports is negative and significant at 0.05 and allows us to reject the null hypothesis of no association. The estimate for exports is positive and significant at 0.01, unexpectedly leading us to reject hypothesis 2 stipulating no association between export levels and the COG. Both estimates have in common that the confidence interval is relatively wide and the uncertainty large.

**Table 5:** Regression results for baseline model and alternative specifications.

	Two-way base	FDI	PCSE	Trade balance
Imports	-0.0064*	-0.0073**	-0.0064**	
	(0.0026)	(0.0026)	(0.0023)	
Exports	0.0057**	0.0064**	0.0057***	
	(0.0022)	(0.0022)	(0.0017)	
FDI		0.0008		
		(0.0005)		
Median voter	0.0876*	0.0780*	0.0876*	0.0756*
	(0.0350)	(0.0313)	(0.0430)	(0.0381)
GDP growth	-0.0218***	-0.0214***	-0.0218***	-0.0224***
	(0.0033)	(0.0037)	(0.0043)	(0.0033)
GDP/capita	-0.0000**	-0.0000**	-0.0000**	-0.0000***
	(0.0000)	(0.0000)	(0.0000)	(0.0000)
Lagged DV	0.3442***	0.3177***	0.3442***	0.3584***
	(0.0531)	(0.0595)	(0.1011)	(0.0570)
Trade balance				0.0047*
				(0.0020)
Constant	-0.6347**	-0.5846**	-0.6347**	-0.5842**
	(0.1936)	(0.1786)	(0.2220)	(0.2150)
R^2^	0.39	0.37	0.39	0.39
N	129	116	129	129

Standard errors in parentheses; two-sided tests; p < .05 *; p < .01 **; p < .001 ***.

Estimates for alternative specifications that confirm the results in Fig 2 are presented in Table 5 and S3 Table. S3 Table shows that the results are robust to using different measurement approaches of party positions on the economic dimension. For each of the five previously used measurement approaches we summarized in Table 2, we get significant and negative estimates for imports and significant and positive estimates for exports. As a further robustness test, we included foreign direct investment (FDI) as an independent variable. We measure it via the net inflows of FDI relative to GDP. We do not expect systematic adjustments of party positions in response to changes in FDI flows because of their lower salience in the public discourse and political competition and lower economic salience. This argument can be stressed with the comparison of the relative size of exports (in our sample on average 44% of GDP) and imports (42% of GDP) with the FDI net inflows (on average 5% of GDP with Luxembourg being an outlier). The inclusion of FDI net inflows reduces the number of available elections from 129 to 116. The effects for exports and imports are robust to the inclusion of FDI and the effects for FDI are small and statistically undistinguishable from zero in pooled and time-variant analyses (see below). We further observe that the results are robust to the estimation of panel-correct standard errors [71].

The parallel development of imports and exports in some countries (S2 Fig) hints at a multicollinearity problem when using both as covariates in the same model. The visual impression is supported by a variance inflation factor (VIF) for the import variable and export variable higher than conventional maximum of 10. The VIF is 35 for exports and 32 for the import variable. Two variables do contain less information, the higher their degree of collinearity and the less precise the estimate of the coefficient becomes. In this perspective, it is a positive finding that the estimates for imports and exports are significant. However, a high degree of multicollinearity also has the potential to render estimates highly sensitive to minor changes in the data ([72], p. 11). As a first robustness test reported in the last column of Table 5, we replace the imports and exports variables with a trade balance variable subtracting imports from exports. This eliminates the multicollinearity problem and follows the recommendation to aggregate collinear variables ([72], pp. 14–15). Following our theoretical arguments, we expect a positive correlation between the trade balance and the COG because a higher level of exports relative to imports should, on average, move parties to the right on the economic dimension. The estimate for the trade balance variable is positive and significant at 0.05.

As a second robustness test, we run the baseline model with case-wise and country-wise deletion. The size and direction of estimates for imports and exports are not sensitive to deleting cases or countries because the point estimates have little variance and the sign of the estimate is always the same. This is an important finding because it indicates that collinearity does not induce sensitivity of the estimates to minor (case-wise deletion) and not so minor (country-wise deletion) changes of the data. We take this as evidence that multicollinearity does not undermine the interpretability of our estimates for imports and exports.

With regard to the significance of the estimates, for imports, we observe that one estimate out of 129 is not significant at .05. This is not evidence of non-robustness because we should expect one non-significant estimate just by chance (S3 Fig). All 129 estimates are significant for exports (S4 Fig). When we estimate the models without Greece (no. 7) or Ireland (no. 8), the estimates for imports and exports cease to be significant at .05 and only achieve significance at .10 (S5 and S6 Figs). The point estimates are in the same narrow range as for the other models and substantively identical. Greece contributes the largest number of observations to the analysis (*n* = 15) and its exclusion reduces the efficiency of the estimates most, which is evidenced by having the greatest confidence interval. Ireland adds eleven cases to the analysis, which equals about nine percent of the total number of cases. We argue that this does not reflect fundamental problems with the inclusion of either of the two countries, but that the estimates lack precision because of collinearity (see above) and because removing 10–15 cases from a sample of 129 cases can make an important difference for statistical significance at a level of .05. At the request of a reviewer, we also estimated one-way and two-way fixed effects models that we discuss in the appendix (S4 Table).

## 6 Analysis of time-variant hypotheses

**Fig 3:**
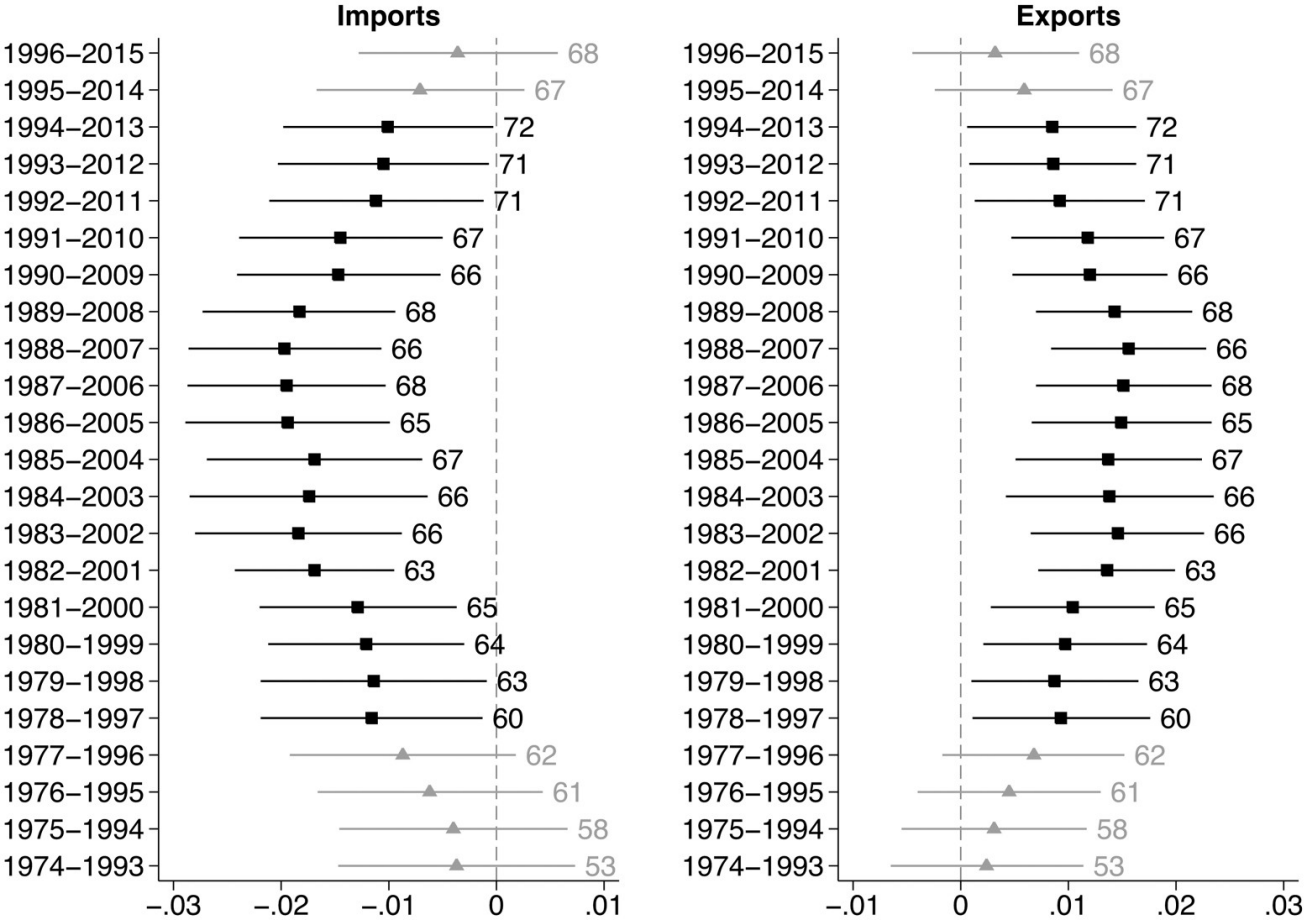
Estimates for imports and exports over time. Numbers next to confidence intervals are observations per regression.

We test hypotheses 3 to 7 with a moving-window analysis estimating the OLS regression with two-way clustered standard and a lagged DV for subsets of the total period of analysis (Fig 3). Each window covers 20 years and is moved forward by one year, taking 1974 as the starting point. The number of observations increases from 53 for the first window up to 68 for the last window because data availability improves for more recent periods. We do not control for multiple testing because the windows have been determined in an exploratory manner as their size and number do not follow from theory. The results are robust to changes in the size and number of windows.

The charts show that the association between imports and exports and the COG varies over time. The marginal effects are close to zero for the early windows, gradually becoming more negative for imports and more positive for exports. This confirms hypothesis 3 stipulating a non-association between exports and the COG in the first part of our period of analysis. The findings do not allow us to reject the null hypothesis for imports, contradicting hypothesis 4 stipulating a negative association between imports and the COG. In the early 1980s, the associations between imports, exports and the COG become statistically distinguishable from zero and have the expected direction, yielding evidence in line with hypotheses 5 (positive effects for exports) and 6 (negative effects for imports) for the second period. The estimates decrease in size and become non-significant again at the end of the third period. Hypothesis 7, stipulating attenuating positive respectively negative effects for exports and imports in the 2000s can be confirmed. We again take multicollinearity problems into account and replace imports and exports with the trade balance variable. The results confirm the findings we present here (S7 Fig).

**Fig 4:**
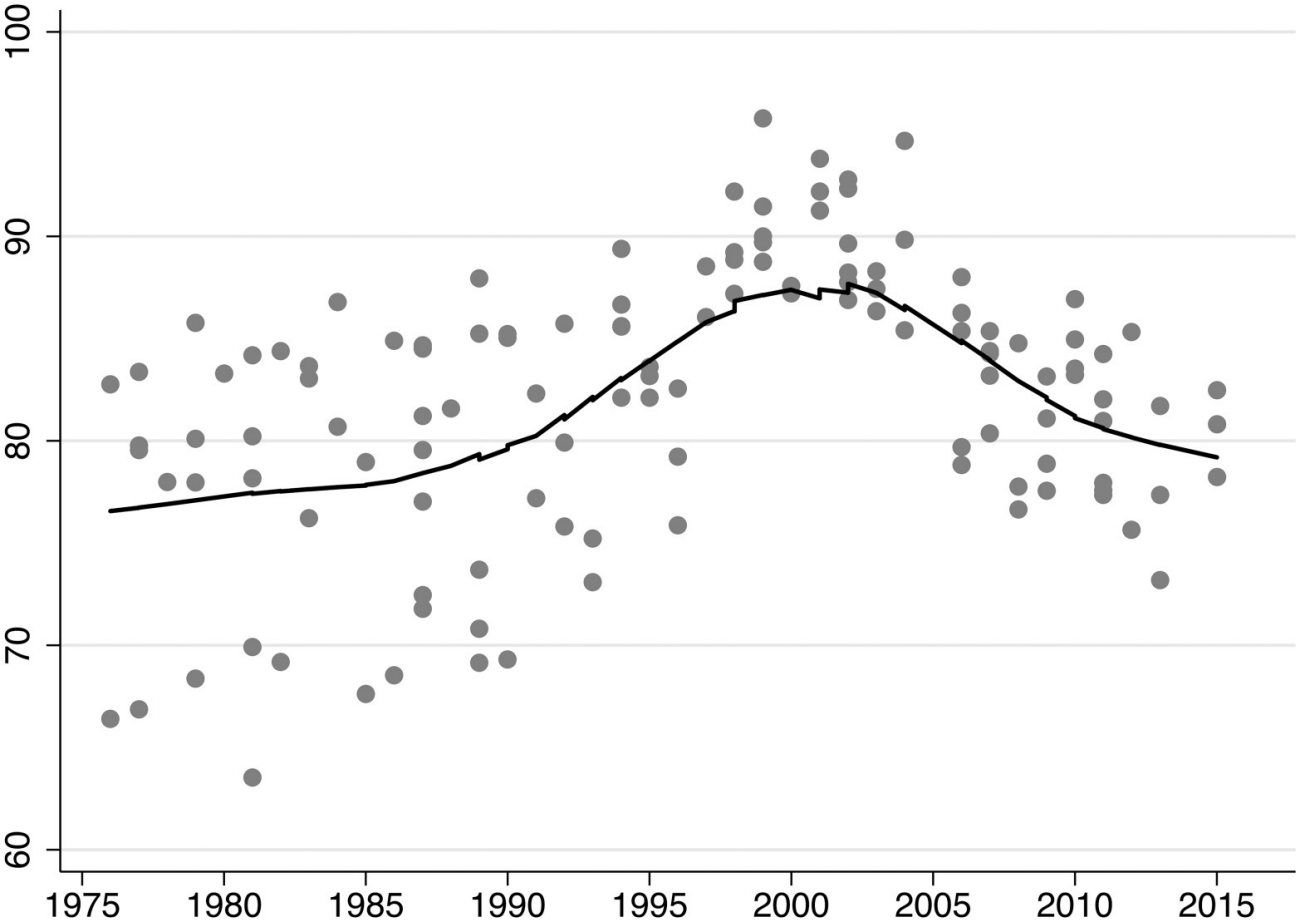
KOF economic globalization index, de jure (one-year lag). Lowess smoother, bandwith = 0.5.

We derived the hypotheses on the time-varying effect of imports and exports from the assumption that the attitudes of parties toward economic globalization changed over the past four decades. Since we are not able to measure these attitudes directly, we test two additional observable implications that should find empirical support if globalization attitudes varied over time. We argue that parties of the right and the left in particular assumed more positive attitudes from the mid-1980s until the early 2000s. Globalization skepticism then increased again because of the stock market crash of 2000 and the economic downturn. If this is correct, we should observe a reduction of barriers to trade and financial flows that lasted until the early 2000s and an increase in impediments to trade and finance thereafter. The implication is based on the argument that attitudes do not only drive party positioning, but also actual policy making. We visually assess this implication by plotting the KOF de jure index for economic globalization over time and trace its development with a Lowess smoother (Fig 4) [73]. For the KOF data, we always present the variable lagged by one year. Higher numbers on the index reflect lower barriers to trade and finance flows. In this analysis, we cannot link any data point to the rationales of a specific left or right party. However, we believe that the data are still indicative because of variation in the partisan composition of governments and parliaments between countries and within countries over time. This variation rules out, for example, that increases in liberalization are exclusively attributable to rightist parties while declining liberalization is solely attributable to leftist parties. The development of de jure economic globalization follows the expected pattern, with a globalization bump around 2000 and a decrease to a de jure globalization level that is similar to the level of the mid-1980s. S8 Fig shows that de jure trade and financial globalization follow the same pattern, with the decline in finance being larger than in trade.

**Fig 5:**
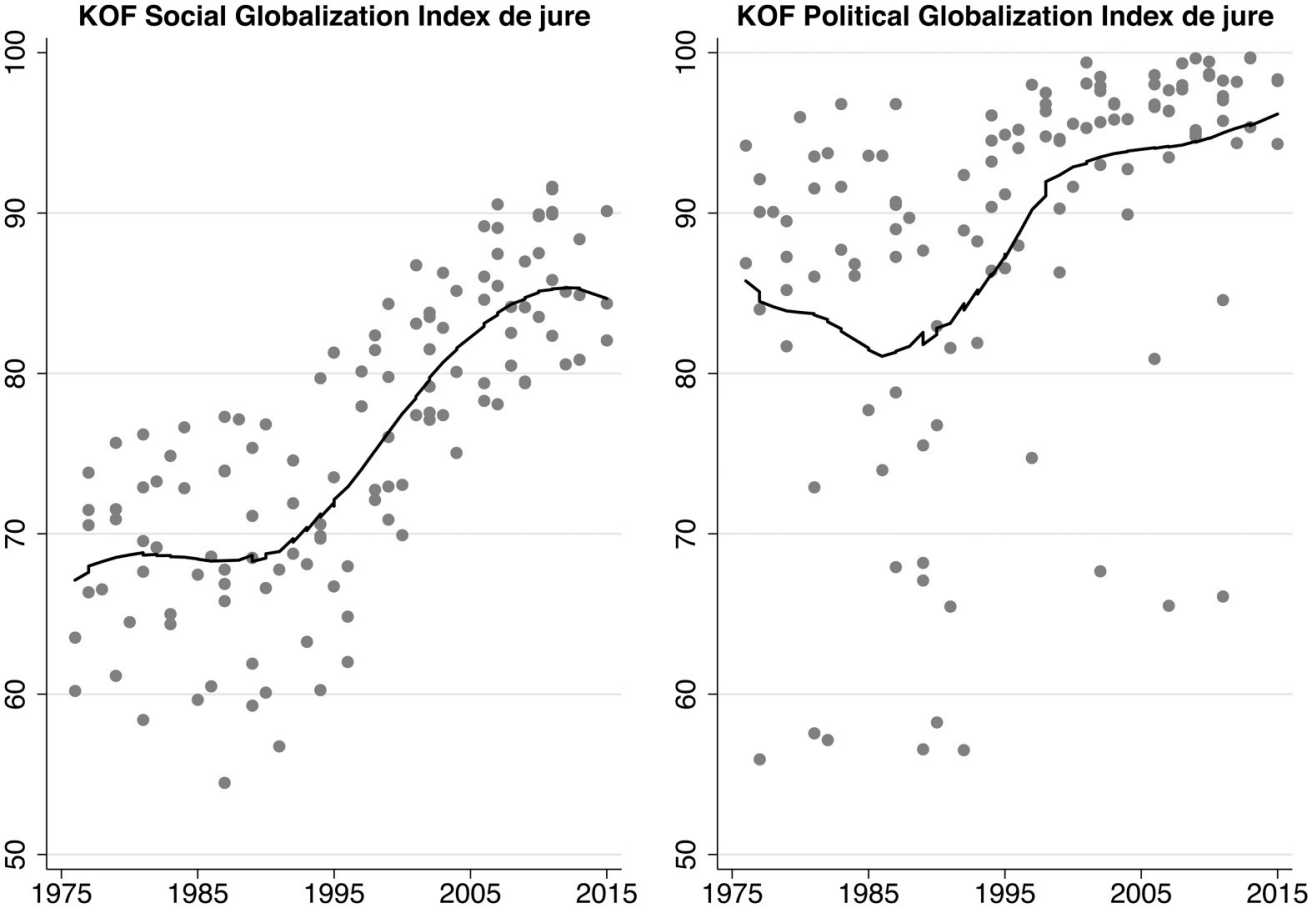
De jure social and political globalization (one-year lag). Lowess smoother, bandwith = 0.5.

The second observable implication is related to the first and focuses on two other globalization dimensions. If attitudes toward *economic* globalization changed over time, we should not see the same pattern of a bump around 2000 for de jure political and social globalization. This implication can be conceived of as an informal placebo test because we do not expect to see the pattern for outcomes other than de jure economic globalization. Fig 5 demonstrates that de jure globalization in the political and social domains follows different patterns. All three domains have increases at the globalization level since the mid-1980s in common. In contrast to economic globalization, political and social de jure globalization keep their level until the end of our period of analysis. The difference in the development of de jure globalization in economics and the other two domains lends additional credence to our argument about time-varying attitudes toward economic globalization.

## 7 Discussion

The empirical results show that economic globalization is associated with the development of a party system’s center of gravity, but the link is different across dimensions of globalization and time. We summarize our findings in Table 6 and compare them with the hypotheses we formulated in Section 3. The two hypotheses that fail to be supported concern the static hypothesis for exports and the hypothesis on imports during the first of our three periods. Our hypothesis on the absence of an effect of exports on the COG was based on the premise that both the parties on the left and right value rising exports, but promote them with different policies, which results in no significant change of the COG on the systematic level. The positive effect of exports suggests that this premise is incorrect. Three non-exclusive reasons can account for this. First, left and right parties propose different policies to foster exports and right parties do this more strongly than left parties, leading to a rightward shift of the COG. A possible reason for this could be that exporting companies might be more influential and more often among the constituency of right parties, which therefore seek export-promoting polices more than left parties. Second, contrary to the compensation hypothesis, changing expectations of voters are also conceivable: Workers in the export sectors who benefit from rising exports by occupying high-skilled jobs and rising wages could demand policies fostering the reason for their economic success that are picked up by party platforms. Third, left parties and right parties might pursue similar, supply-side policies when it comes to the promotion of exports.

The failure to reject the null hypothesis of no effects of imports on the COG during the first period of analysis can be attributed to the bad state of the world economy in the mid-1970s and early 1980s. The breakdown of the Bretton Woods system and looming problems of the world economy in the early 1970s might have led to a leftward shift of the COG in the years before the start of our period of analysis and that we expected to happen in the late 1970s. Once left and right parties adjusted their positions leftward because of undesired imports during bad economic times, there was then ideological stagnation during the first subperiod of analysis until economic globalization took off since the mid-1980s. We find this explanation most plausible because it is not obvious to us why parties that traditionally see imports as something negative would not react by becoming more protectionist during economic downturns.

**Table 6:** Results and expectations (in parentheses).

	Exports	Imports
**Pooled effect**	**+** (o)	**−** (−)
**Time-variant effects**		
1970s—mid 1980s	**o** (o)	**o** (−)
mid 1980s—early 2000s	**++** (++)	**− −** (− −)
2000s	**+** (+)	**−** (−)

**Fig 6:**
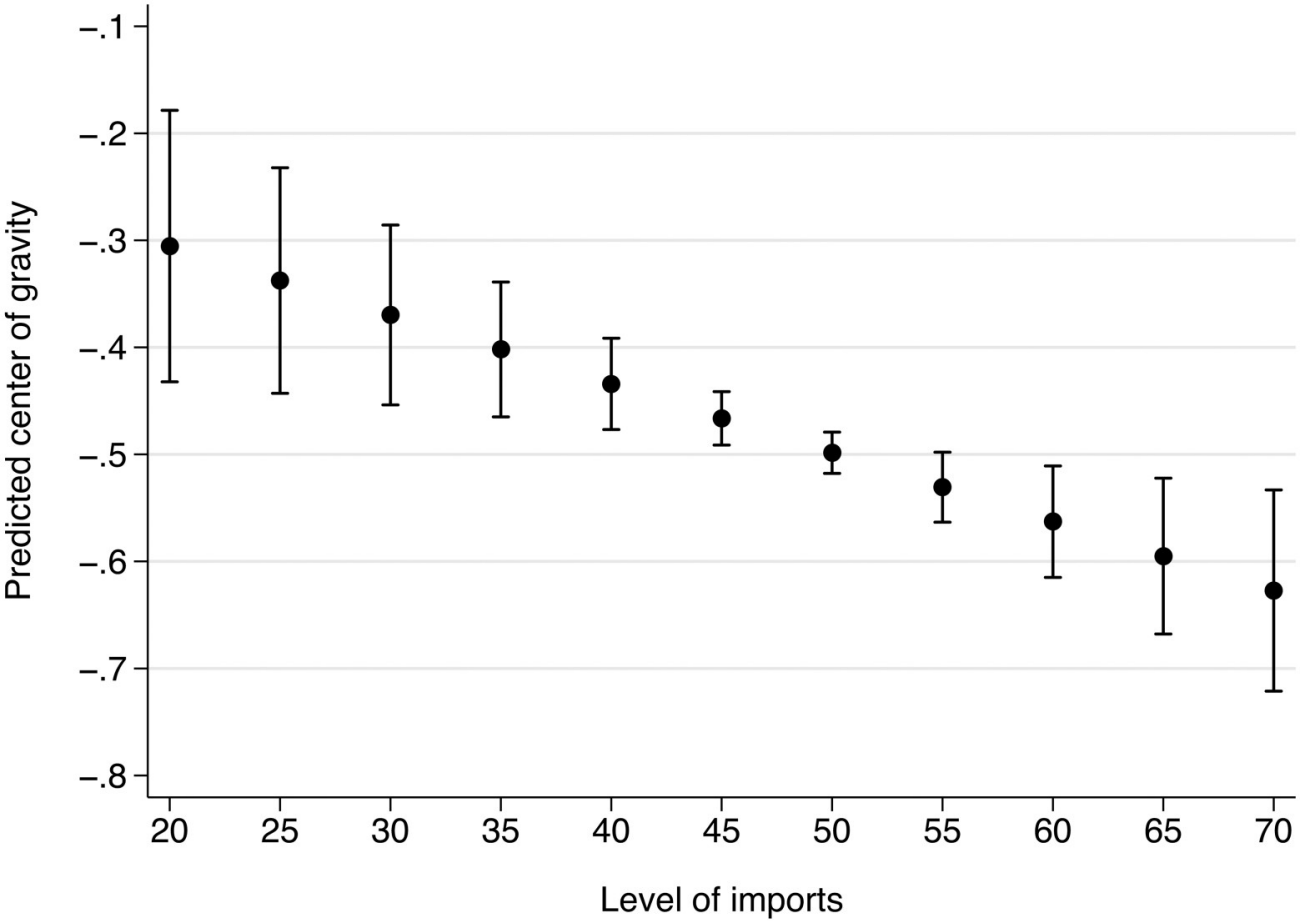
Predicted center of gravity for imports for export level of 50%.

We enhance the substantive interpretation of the estimates by plotting the predicted COG and 95% confidence interval for levels of imports ranging from 20% to 70% in steps of 5%. S2 Fig shows that this is the range of import levels for most countries. We fix the exports variable at a value of 50%. This gives us a conservative predicted COG because exports have the opposite effect on the COG than imports. The predicted COG moves from about -0.31, which is predicted with large uncertainty, to about -0.63, which is predicted with higher precision (Fig 6). We take this to be a substantively significant effect because imports have been continuously growing in our period of observation.

**Fig 7:**
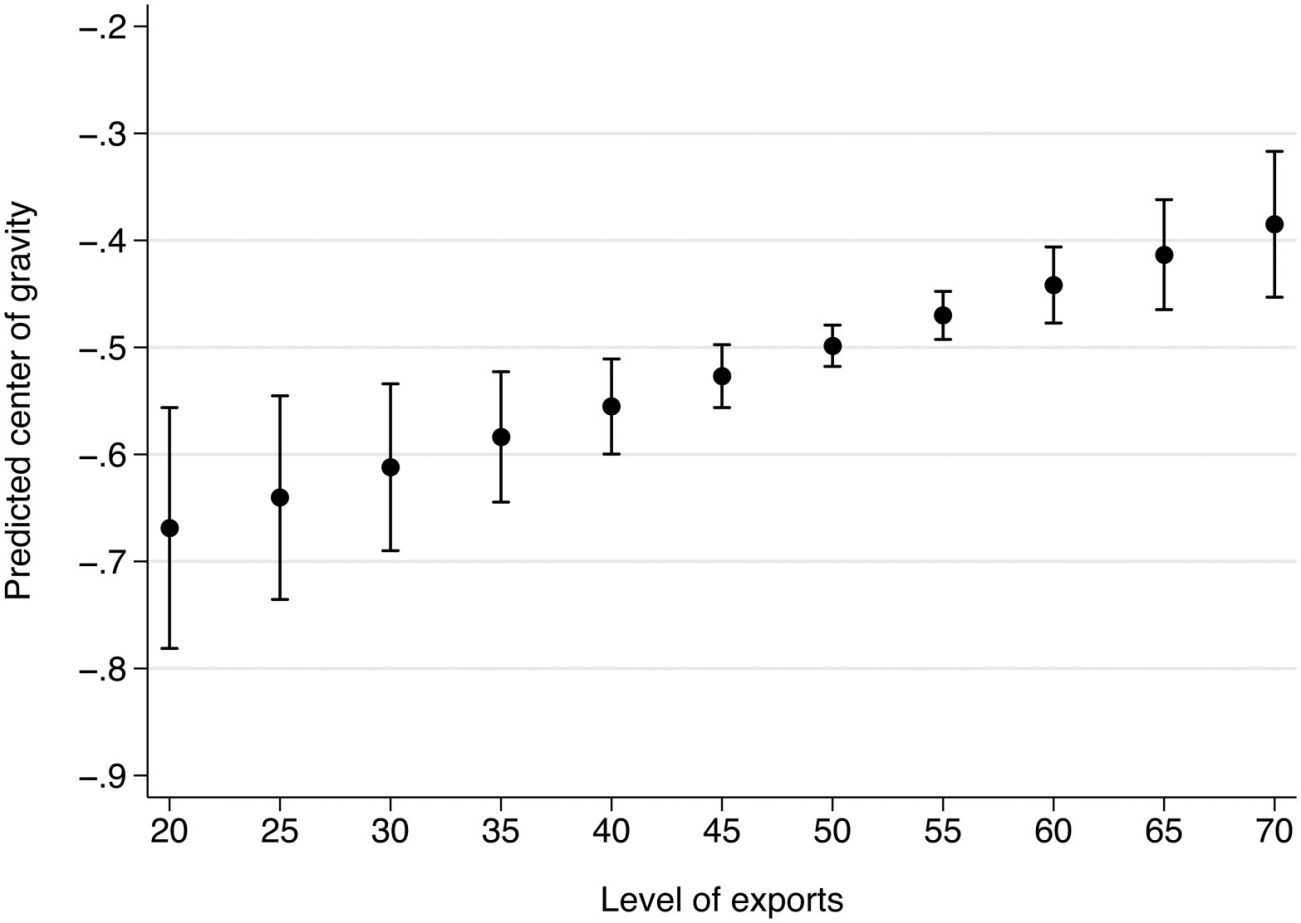
Predicted center of gravity for exports for import level of 50%.

Fig 7 presents the equivalent plot for export levels and import levels fixed at 50%. The predicted COG becomes more centrist as the level of exports increases, but the level of the COG is lower for the equivalent level of imports. The predicted COG is about -0.67 for export levels of 20% and at about -0.63 for import levels of 70%. The same holds for the opposite end of the spectrum, with a prediction of about -0.38 for the maximum level of exports, 70%, and of about -0.31 for the lower level of imports. In comparison and based on the larger absolute estimate for imports (see Table 5), imports have a slightly stronger effect on the COG, leading us to expect a more leftist COG than equivalent levels of exports.

We conclude the discussion by addressing potential problems of endogeneity, reverse causation and identification. First, economic globalization is not a force of nature, but has been made possible by political decisions [1]. This might create concerns about endogeneity between trade and investment flows and party positions and reverse causation according to which parties first change their position [74], with systemic consequences for the COG, and then reduce barriers to trade. We believe that these are not problems for our analysis because most liberalization measures have been negotiated under the auspices of international institutions such as the GATT or in regional or bilateral trade treaties [21, 75]. These agreements phase-in trade and investment liberalization measures, for example, by gradually reducing a tariff over multiple years instead of making a huge cut in one year [76]. In addition, economic actors need to adjust to such phased-in measures and trade and investment flows follow these changes with a certain degree of delay. For this reason, trade and investment flows at year *t* should be explained with partisan positions during the negotiations of an agreement and be unrelated to the positions that parties took in the year *t*-1. An additional reason speaking against endogeneity and reverse causation is that most countries in our data have been members of the EU for the greatest part of the period of analysis. Since the 1960s, the Commission of the EU has been negotiating trade treaties on behalf of its member states, implying that the liberalization measures and downstream effects on trade and investment flows are not directly influenced by the positions of parties. The Commission of the EU is in contact with the governments of its member states. Because of the large number of member states and the fact that many governments are coalition governments, the bargaining position of the Commission is, if at all, a compromise of the individual party positions. This breaks the link between any individual party position, the final negotiation outcome and downstream economic effects.

Second, an alternative interpretation for our findings is that there might be different effects of imports and exports, but they only mask broader globalization effects that we cannot capture with a disaggregated perspective. We use the KOF *de facto* globalization index as an independent variable to test whether there are general effects of economic globalization [73]. The *de facto* dimension of economic globalization has the two subdimensions, ‘trade globalization’ and ‘financial globalization’, comprising a total of eight variables (see [77, 78]). We run four models with the economic globalization index, the trade index, the financial index and the trade and finance index combined. In each model, the index or indices replace the import, export and FDI variables (S5 Table). None of the index estimates are significant in any of the four models and we are unable to reject the null hypothesis of aggregated globalization effects on party systems. There might be aggregate effects that are smaller than those we can detect with our data, but we take this as supporting evidence for a disaggregated perspective on individual economic variables.

Third, even if there were no aggregated economic effects, a criticism might be that the findings for imports and exports only represent a broader globalization trend, which also includes the political and social realm. We use the measures for de facto political and social globalization of the KOF index to test this argument. We run two models each with political globalization and social globalization as a covariate and a third model with economic, political and social globalization combined (S6 Table). The estimates are not significant in any of these models. We cannot reject the null of no effects of political and social globalization on the center of gravity and interpret this as evidence that the significant estimates for imports and exports are genuine economic effects and do not reflect a broader globalization effect.

Fourth, the effect of economic variables on party systems is difficult to identify because of the interplay of the dimensions of economic globalization and because economic variables, public opinion and party behavior are interrelated in ways that we cannot properly model in an observational setting. We have formulated causal hypotheses on the causal effect of globalization and party systems and the underlying mechanisms, but the empirical strategy does not allow us to conclude that the results can be interpreted in causal terms. We have found empirical associations between the dimensions of economic globalization and the COG that are indicative of a causal relationship, but it should be kept in mind that the evidence has been derived from a model-based analysis of observational data that confronts identification issues [79].

## 8 Conclusion

Political parties are involved in making decisions about the trajectory of globalization. We reverse the perspective and present results indicating that imports and exports, as the two most important facets of economic globalization, might influence the positions that parties take on an economic dimension of party competition. If correct (see previous section), this has important empirical consequences for voter representation because the average positioning of parties reflects the options from which voters can choose. On a more general level, our study indicates that, as two other salient concepts in the globalization literature, the COG and convergence/divergence are best considered in conjunction to formulate a complete picture.

The estimates of our pooled and dynamic analysis are in discord with previous research that found no or inconsistent effects of globalization on party positioning using pooled data [6, 80]. The variation in the findings suggests that one should be careful in making inferences across levels of analysis, which is parties and party systems for our research topic, to avoid aggregation problems when making bottom-up inferences and problems of ecological inference for top-down conclusions. In addition, the findings of our moving-window analysis point to the benefits of going beyond a pooled analysis and analyze time-varying effects of globalization. This is particularly promising when the pooled dataset spans multiple decades and different globalization episodes and political and economic paradigms. Because of the challenges to the causal analysis that we addressed in the previous section, our results are best considered a promising basis for future studies on globalization effects along those lines that engage in design-based inference to make causal inferences on stronger ground.

## Supporting information

S1 Table Variables, operationalization and sources. (PDF)

S2 Table Countries and elections. (PDF)

S3 Table Regression results for alternative constructions of the dependent variable. (PDF)

S4 Table Regression results for fixed-effects models. (PDF)

S5 Table Results for KOF indices of de facto economic globalization. (PDF)

S6 Table Results for KOF de facto economic, political and social globalization. (PDF)

S7 Table Descriptive statistics for KOF indices. (PDF)

S1 Fig Elections per year. (TIF)

S2 Fig Development of imports and exports. (TIF)

S3 Fig Estimates for imports in baseline model with case-wise deletion. (TIF)

S4 Fig Estimates for exports in baseline model with case-wise deletion. (TIF)

S5 Fig Country-wise deletion, imports. 1 Austria, 2 Belgium, 3 Cyprus, 4 Denmark, 5 Finland, 6 Germany, 7 Greece, 8 Ireland, 9 Italy, 10 Luxembourg, 11 Netherlands, 12 Portugal, 13 Spain, 14 Sweden, 15 United Kingdom.
(TIF)

S6 Fig Country-wise deletion, exports. 1 Austria, 2 Belgium, 3 Cyprus, 4 Denmark, 5 Finland, 6 Germany, 7 Greece, 8 Ireland, 9 Italy, 10 Luxembourg, 11 Netherlands, 12 Portugal, 13 Spain, 14 Sweden, 15 United Kingdom.
(TIF)

S7 Fig Model with trade balance, moving-window analysis. Numbers next to confidence intervals are observations per regression. We run the moving-window analysis with the trade balance variable instead of imports and exports to account for multicollinearity in the baseline model because of the correlation between imports and exports. The results confirm the theoretical arguments and findings and the estimates for trade balance display the expected development. The effects become positively significant during the second subperiod and tend to get smaller and remain significant in the third subperiod. As before, the confidence intervals are wide and the coefficient is estimated with relatively large uncertainty.
(TIF)

S8 Fig De jure trade and financial globalization (one-year lag). Lowess smoother, bandwith = 0.5.
(TIF)

S1 File Stata log-file. (PDF)
