# Supplementary material for: The time-varying relationship between economic globalization and the ideological center of gravity of party systems
Source: PLoS One. 2019 Feb 27;14(2):e0212945. doi: 10.1371/journal.pone.0212945 (PMC6392286; doi:10.1371/journal.pone.0212945)
Supplement: S1 Table — (PDF) [file pone.0212945.s001.pdf]

S1 Table. Variables, operationalization and sources.

Variable	Operationalization	Source
Center of gravity	Left-right position of parliamentary parties on economic dimension weighted by their seat share	Volkens et al.
Import level	Imports in percentage of GDP (lagged by one year)	WDI
Export level	Imports in percentage of GDP (lagged by one year)	WDI
Public opinion	Mean (overall) left-right position of citizens (lagged by one year)	Eurobarometer
GDP growth	Annual change of real GDP in percent (lagged by one year)	WDI
GDP per capita	GDP per capita in thousand USD (lagged by one year)	WDI
Net inflow of foreign direct investment	Foreign direct investment inflow in percentage of GDP (lagged by one year)	WDI
Trade balance	Difference between exports and imports relative to GDP (lagged by one year)	WDI
KOF de facto and de jure globalization indices	Indices based on different measures of globalization (lagged by one year)	Gygli et al.
