# Supplementary material for: The time-varying relationship between economic globalization and the ideological center of gravity of party systems
Source: PLoS One. 2019 Feb 27;14(2):e0212945. doi: 10.1371/journal.pone.0212945 (PMC6392286; doi:10.1371/journal.pone.0212945)
Supplement: S2 Table — (PDF) [file pone.0212945.s002.pdf]

S2 Table. Countries and elections.

Country	Elections
Austria	1999; 2002; 2006; 2008
Belgium	1977; 1978; 1981; 1985; 1987; 1991; 1995; 1999; 2003; 2007; 2010
Cyprus	2006; 2011
Denmark	1977; 1979; 1981; 1984; 1987; 1988; 1990; 1994; 1998; 2001; 2007; 2011
Finland	1995; 1999; 2003; 2007; 2011
Germany	1976; 1980; 1983; 1987; 1990; 1994; 1998; 2002; 2009; 2013
Greece	1981; 1985; 1989; 1989; 1990; 1993; 1996; 2000; 2004; 2007; 2009; 2012; 2012; 2015; 2015
Ireland	1977; 1981; 1982; 1982; 1987; 1989; 1992; 1997; 2002; 2007; 2011
Italy	1976; 1979; 1983; 1987; 1992; 1994; 1996; 2001; 2006; 2008; 2013
Luxembourg	1979; 1984; 1989; 1994; 1999; 2004; 2009; 2013
Netherlands	1977; 1981; 1982; 1986; 1989; 1994; 1998; 2002; 2003; 2006; 2010; 2012
Portugal	1987; 1991; 1995; 1999; 2002; 2009; 2011
Spain	1986; 1989; 1993; 1996; 2000; 2004; 2008; 2011; 2015
Sweden	1998; 2002; 2006; 2010
United Kingdom	1979; 1983; 1987; 1992; 1997; 2001; 2010; 2015
