# Supplementary material for: The time-varying relationship between economic globalization and the ideological center of gravity of party systems
Source: PLoS One. 2019 Feb 27;14(2):e0212945. doi: 10.1371/journal.pone.0212945 (PMC6392286; doi:10.1371/journal.pone.0212945)
Supplement: S3 Table — (PDF) [file pone.0212945.s003.pdf]

S3 Table. Regression results for alternative constructions of the dependent variable.

	**Baseline model**	Laver/ Budge	Benoit/ Laver	Tavits	Bakker/ Hobolt	Prosser
Imports	-0.0064* (0.0026)	-0.0189*** (0.0041)	-0.0109*** (0.0028)	-0.0168*** (0.0032)	-0.0070* (0.0027)	-0.0076*** (0.0022)
Exports	0.0057** (0.0022)	0.0151*** (0.0031)	0.0098*** (0.0024)	0.0130*** (0.0025)	0.0063** (0.0022)	0.0065*** (0.0018)
Median voter	0.0876* (0.0350)	0.1822** (0.0571)	0.1058* (0.0409)	0.2161*** (0.0454)	0.0653 (0.0381)	0.0929* (0.0408)
GDP growth	-0.0218*** (0.0033)	-0.0123 (0.0090)	-0.0253*** (0.0052)	-0.0171* (0.0069)	-0.0213*** (0.0046)	-0.0215*** (0.0041)
GDP/ capita	-0.0000** (0.0000)	-0.0000 (0.0000)	-0.0000* (0.0000)	-0.0000 (0.0000)	-0.0000** (0.0000)	-0.0000* (0.0000)
Lagged DV	0.3442*** (0.0531)	0.2556 (0.1739)	0.3441*** (0.0735)	0.2835 (0.1680)	0.3711*** (0.0807)	0.2710*** (0.0106)
Constant	-0.6347** (0.1936)	-0.4676 (0.3044)	-0.6612** (0.2101)	-0.6544** (0.2372)	-0.4171* (0.2018)	-0.8898*** (0.2091)
Adj. R^2	0.36	0.17	0.33	0.20	0.34	0.28
N	129	129	129	129	129	129

Standard errors in parentheses; two-sided tests; p < .05 *; p < .01 **; p < .001 ***.
