# Supplementary material for: The time-varying relationship between economic globalization and the ideological center of gravity of party systems
Source: PLoS One. 2019 Feb 27;14(2):e0212945. doi: 10.1371/journal.pone.0212945 (PMC6392286; doi:10.1371/journal.pone.0212945)
Supplement: S4 Table — (PDF) [file pone.0212945.s004.pdf]

S4 Table. Baseline model with one-way and two-way fixed effects.

	Country FE	Year FE	Two-way FE
Imports	-0.0049<br>(0.0047)	-0.0055<br>(0.0041)	-0.0005<br>(0.0061)
Exports	0.0061<br>(0.0037)	0.0049<br>(0.0036)	0.0074<br>(0.0044)
Median voter	0.0639<br>(0.0590)	0.0735<br>(0.0384)	0.0941<br>(0.0648)
GDP growth	-0.0205***<br>(0.0055)	-0.0149<br>(0.0083)	-0.0112<br>(0.0084)
GDP/capita	-0.0000*<br>(0.0000)	-0.0000<br>(0.0000)	-0.0000*<br>(0.0000)
Lagged DV	0.2848***<br>(0.0846)	0.3915***<br>(0.0824)	0.3104***<br>(0.0914)
Constant	-0.7496*<br>(0.3328)	-0.6652**<br>(0.2295)	-1.2102**<br>(0.4029)
Country FE	Yes	No	Yes
Year FE	No	Yes	Yes
R^2	0.45	0.62	0.70
N	129	129	129

Standard errors in parentheses; two-sided tests; p < .05 *; p < .01 **; p < .001 ***.

As requested by a reviewer, we estimate models with one-way and two-way fixed effects (FE). In all models, the coefficient for imports and exports is no longer significant; however, we attach no meaning to these results for the following reasons.

First, as we noted in the manuscript, the F-tests, which are standard for testing as to whether fixed effects should be estimated, are non-significant. Following the econometric literature [1, p. 284], this leads us to decide against the estimation of fixed-effects models. It is correct that the p-value for the F-test for the period FE is small. However, it is above .10, which is, by convention, the maximally acceptable (two-sided) p-value threshold used in a discussion of marginal effects. We do neither see why the interpretation of the p-value should be much more liberal for specification tests nor do we know of any econometric text making this recommendation.

Second, the reviewer noted that some period dummies are significant, which is correct. A look at the country dummies shows four that are also significant ($p < 0.05$), meaning that the *share* of significant country dummies is higher than for year dummies. However, the p-value for the F-test for country FE is large, at about 0.62. In our view, this means that selected significant dummies are not indicative of the need to estimate FE. To our knowledge, the econometric literature does not attach substantive or statistical meaning to the estimates for selected dummies [1, p. 284], but focuses solely on the F-test [for example, 2, p. 281].

Third, the use of a lagged DV with fixed effects became the “Beck-Katz standard” in political science. However, the econometric literature offers a more nuanced discussion of FE estimation. Plümper et al. [3, p. 333-334] show that the inclusion of unit fixed effects is problematic when the theoretical interest is on the level of a variable, which is the case here. In a recent paper, Plümper and Troeger [4] show that unit FE can lead to poorer estimates if the model is dynamically misspecified. Although we wish we had modeled the dynamics correctly, Plümper and Troeger argue that this is very challenging, even when a lagged DV model is estimated, because dynamic misspecification can take many forms. Angrist and Pischke [5, chapter 5] note that the assumption of time-invariant effects that can be captured with FE is demanding and usually not plausible, which also is our position.

Fourth, FE demean the variables. In a two-way FE model, the variables are demeaned within and between units, leaving little variation left that is to be explained, which is, in our view, a high price to pay when the F-test suggests FE are not needed in the first place. Equally important, our theoretical perspective does not require us to use country or period FE. For example, country FE could be in order when the hypotheses explicitly refer to changes over time. Since we are equally interested in explaining and modeling variation between and within units, we formulated our hypotheses accordingly.

1. Beck N. Time-Series-Cross-Section-Data: What Have We Learned in the Past Few Years? Annual Review of Political Science. 2001;4:271-93.
2. Kittel B, Winner H. How reliable is pooled analysis in political economy? The globalization-welfare state nexus revisited. European Journal of Political Research. 2005;44(2):269-93.
3. Plümper T, Troeger V, Manow P. Panel data analysis in comparative politics: Linking method to theory. European Journal of Political Research. 2005;44(2):327-54.
4. Plümper T, Troeger VE. Not so Harmless After All: The Fixed-Effects Model. Political Analysis. 2018;advance access:1-25. 2018/12/04. doi: 10.1017/pan.2018.17.
5. Angrist JD, Pischke J-S. Mostly Harmless Econometrics: An Empiricist's Companion. Princeton: Princeton University Press; 2009.
