# Supplementary material for: The time-varying relationship between economic globalization and the ideological center of gravity of party systems
Source: PLoS One. 2019 Feb 27;14(2):e0212945. doi: 10.1371/journal.pone.0212945 (PMC6392286; doi:10.1371/journal.pone.0212945)
Supplement: S5 Table — (PDF) [file pone.0212945.s005.pdf]

S5 Table. Results for KOF indices of de facto economic globalization.

	Economic	Trade	Financial	Trade and Financial
Economic Globalization	-0.0010<br>(0.0008)			
Trade Globalization		-0.0009<br>(0.0007)		-0.0004<br>(0.0009)
Financial Globalization			-0.0009<br>(0.0008)	-0.0006<br>(0.0010)
Median voter	0.0947*<br>(0.0477)	0.0950*<br>(0.0459)	0.0890<br>(0.0462)	0.0941*<br>(0.0452)
GDP growth	-0.0194***<br>(0.0038)	-0.0194***<br>(0.0039)	-0.0197***<br>(0.0037)	-0.0195***<br>(0.0039)
GDP/capita	-0.0000**<br>(0.0000)	-0.0000**<br>(0.0000)	-0.0000**<br>(0.0000)	-0.0000**<br>(0.0000)
Lagged DV	0.3606***<br>(0.0561)	0.3612***<br>(0.0539)	0.3641***<br>(0.0570)	0.3609***<br>(0.0564)
Constant	-0.6703**<br>(0.2393)	-0.6843**<br>(0.2420)	-0.6403**<br>(0.2285)	-0.6665**<br>(0.2235)
Adj. R^2	0.35	0.35	0.35	0.34
N	129	129	129	129

Standard errors in parentheses; two-sided tests; p < .05 *; p < .01 **; p < .001 ***.
