# Supplementary material for: The time-varying relationship between economic globalization and the ideological center of gravity of party systems
Source: PLoS One. 2019 Feb 27;14(2):e0212945. doi: 10.1371/journal.pone.0212945 (PMC6392286; doi:10.1371/journal.pone.0212945)
Supplement: S6 Table — (PDF) [file pone.0212945.s006.pdf]

S6 Table. Results for KOF de facto economic, political and social globalization.

	Political	Social	All dimensions
Political Globalization	0.0010<br>(0.0010)		0.0012<br>(0.0010)
Social Globalization		-0.0044<br>(0.0027)	-0.0040<br>(0.0032)
Economic Globalization			-0.0005<br>(0.0010)
Median voter	0.0848*<br>(0.0416)	0.1031*<br>(0.0405)	0.1182*<br>(0.0471)
GDP growth	-0.0188***<br>(0.0040)	-0.0189***<br>(0.0042)	-0.0173***<br>(0.0041)
GDP/capita	-0.0000**<br>(0.0000)	-0.0000<br>(0.0000)	-0.0000<br>(0.0000)
Lagged DV	0.3626***<br>(0.0564)	0.3890***<br>(0.0484)	0.3671***<br>(0.0603)
Constant	-0.7549**<br>(0.2461)	-0.4655**<br>(0.1765)	-0.6732*<br>(0.2986)
Adj. R^2	0.34	0.36	0.36
N	129	129	129

Standard errors in parentheses; two-sided tests; p < .05 *; p < .01 **; p < .001 ***.
