# Supplementary material for: The time-varying relationship between economic globalization and the ideological center of gravity of party systems
Source: PLoS One. 2019 Feb 27;14(2):e0212945. doi: 10.1371/journal.pone.0212945 (PMC6392286; doi:10.1371/journal.pone.0212945)
Supplement: S7 Table — (PDF) [file pone.0212945.s007.pdf]

S7 Table. Descriptive statistics for KOF indices.

		Mean	Standard Deviation	Minimum	Maximum	N
de facto	Economic Globalization	64.02	19.511	22.25	92.73	129
	Trade Globalization	56.35	19.82	22.12	89.21	129
	Financial Globalization	71.69	21.46	18.08	99.32	129
	Social Globalization	74.42	8.30	50.34	89.29	129
	Political Globalization	85.84	12.34	42.99	99.36	129
de jure	Economic Globalization	81.78	6.68	63.52	95.77	129
	Trade Globalization	85.09	8.18	60.38	98.66	129
	Financial Globalization	78.41	7.71	63.24	96.06	129
	Social Globalization	75.48	9.02	54.47	91.65	129
	Political Globalization	88.72	11.43	55.93	99.70	129
