# Supplementary figures and images for: The time-varying relationship between economic globalization and the ideological center of gravity of party systems
Source: PLoS One. 2019 Feb 27;14(2):e0212945. doi: 10.1371/journal.pone.0212945 (PMC6392286; doi:10.1371/journal.pone.0212945)

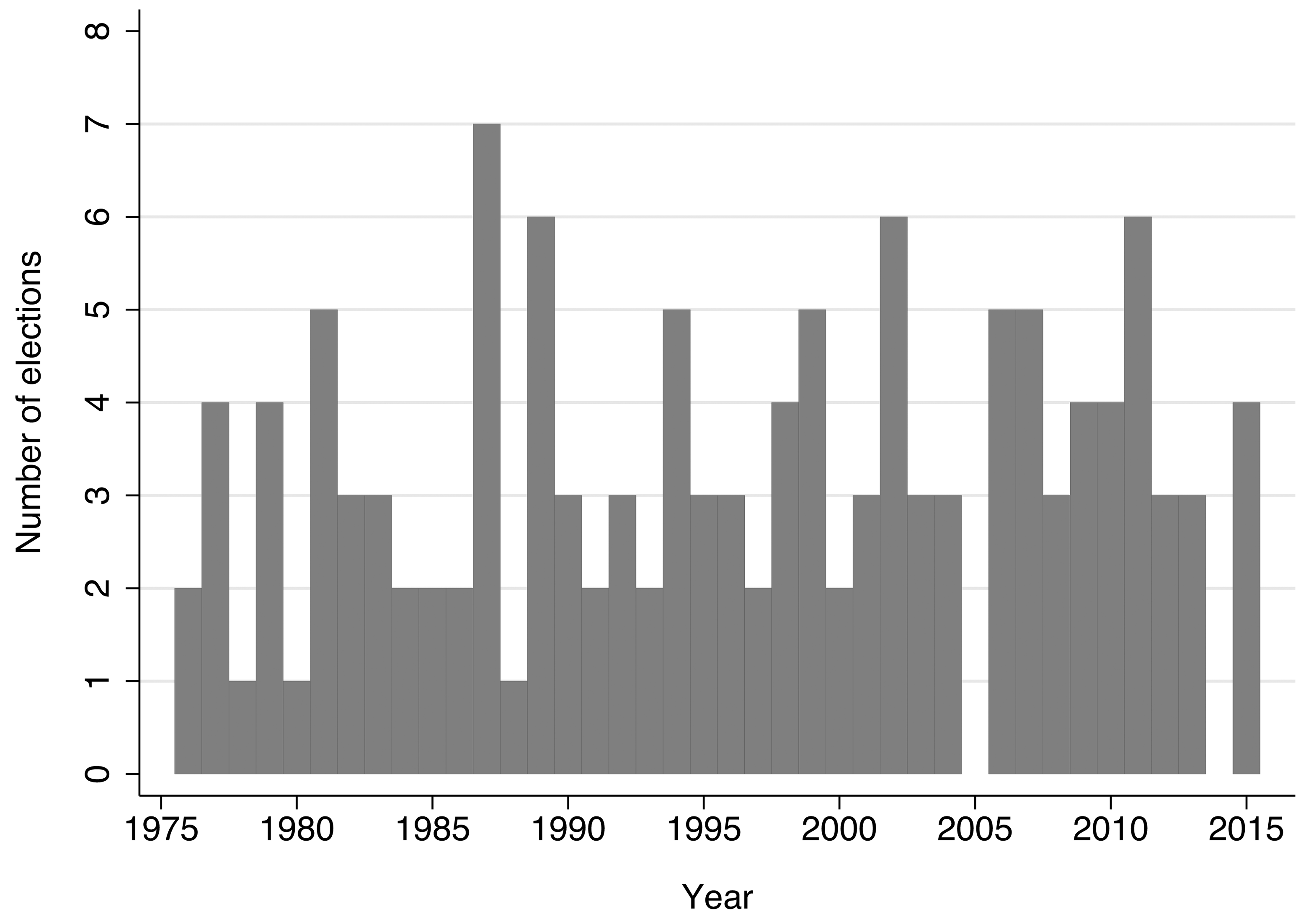

Supplement: S1 Fig — (TIF) [file pone.0212945.s008.tif]

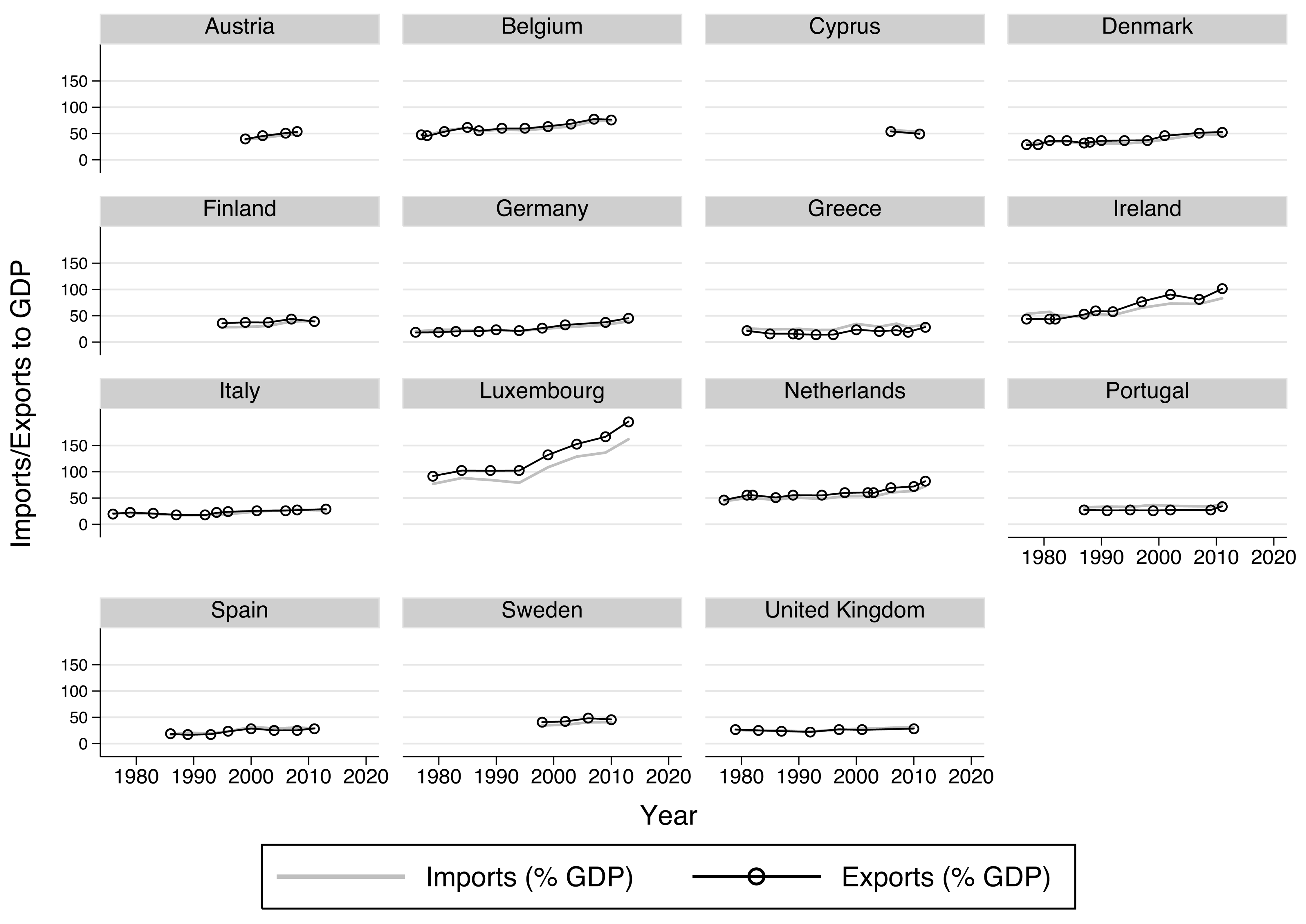

Supplement: S2 Fig — (TIF) [file pone.0212945.s009.tif]

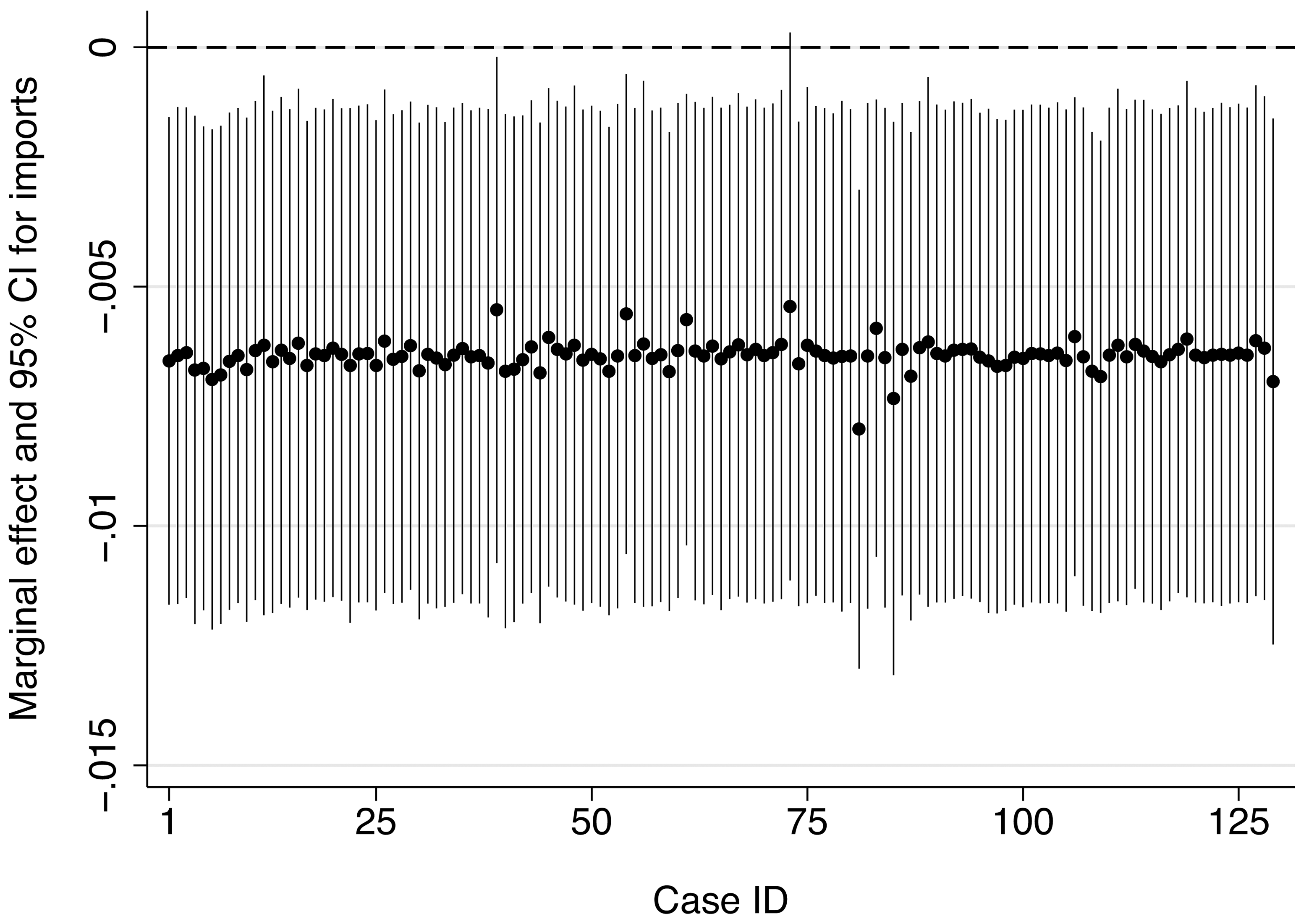

Supplement: S3 Fig — (TIF) [file pone.0212945.s010.tif]

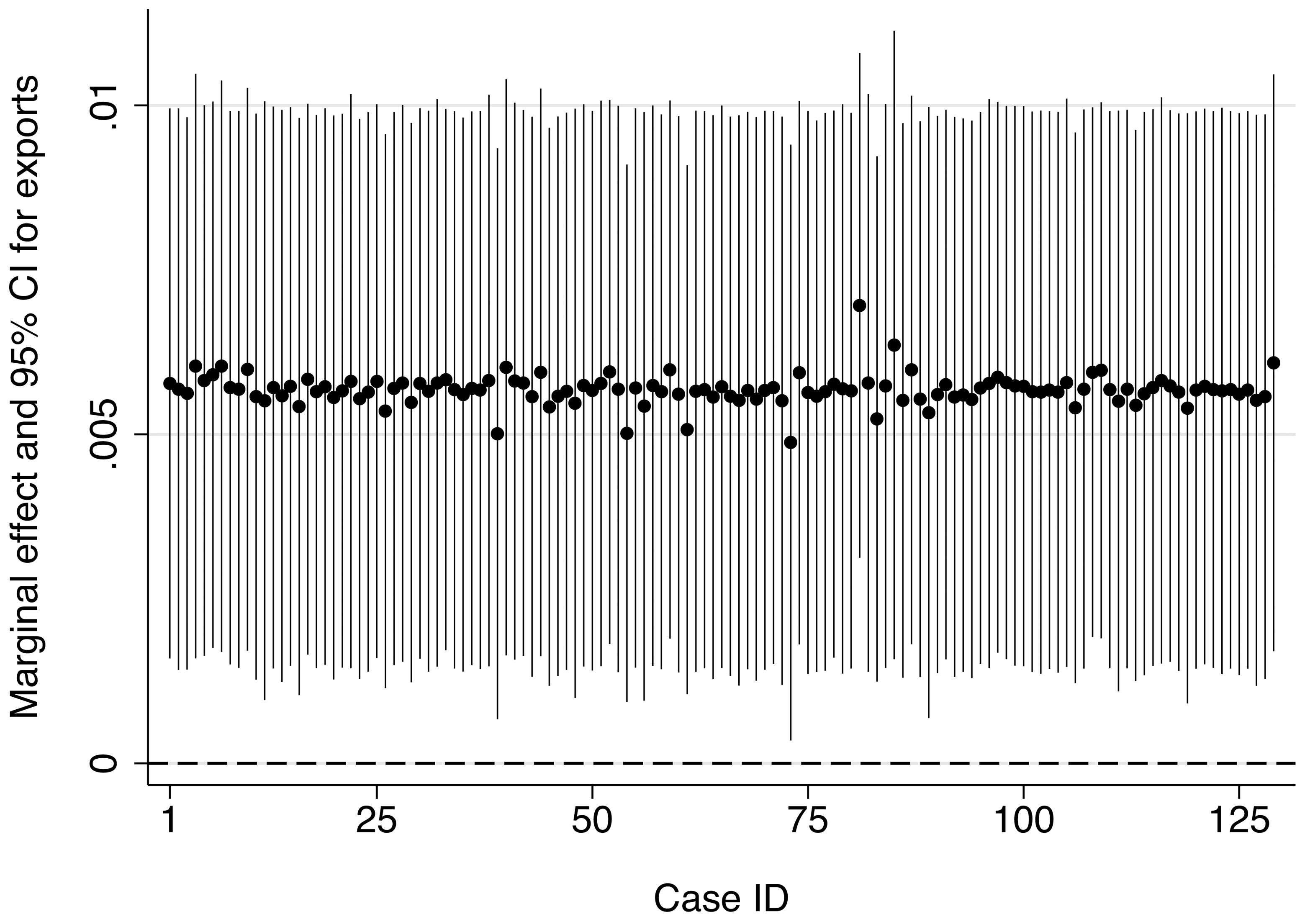

Supplement: S4 Fig — (TIF) [file pone.0212945.s011.tif]

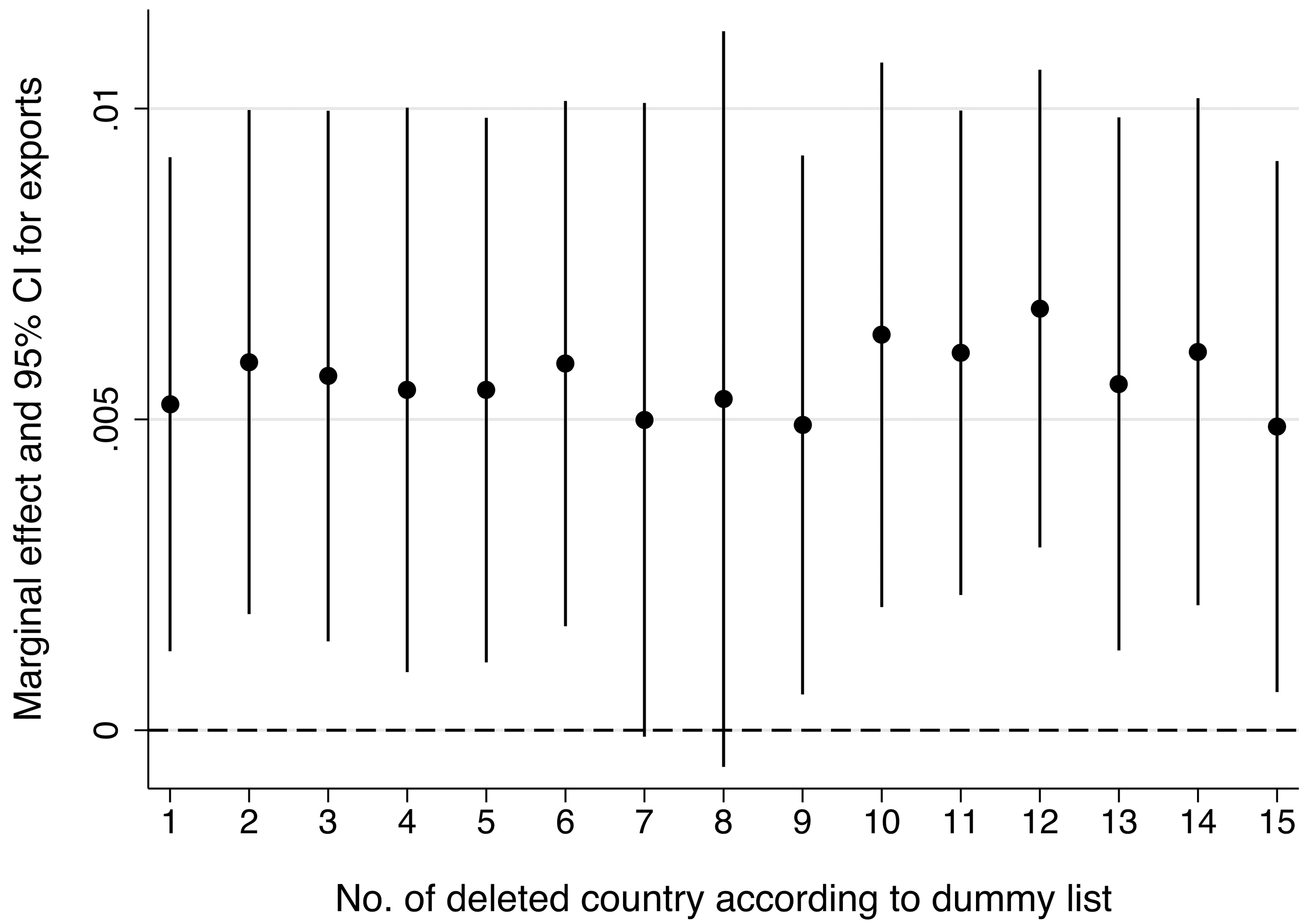

Supplement: S5 Fig — 1 Austria, 2 Belgium, 3 Cyprus, 4 Denmark, 5 Finland, 6 Germany, 7 Greece, 8 Ireland, 9 Italy, 10 Luxembourg, 11 Netherlands, 12 Portugal, 13 Spain, 14 Sweden, 15 United Kingdom. (TIF) [file pone.0212945.s012.tif]

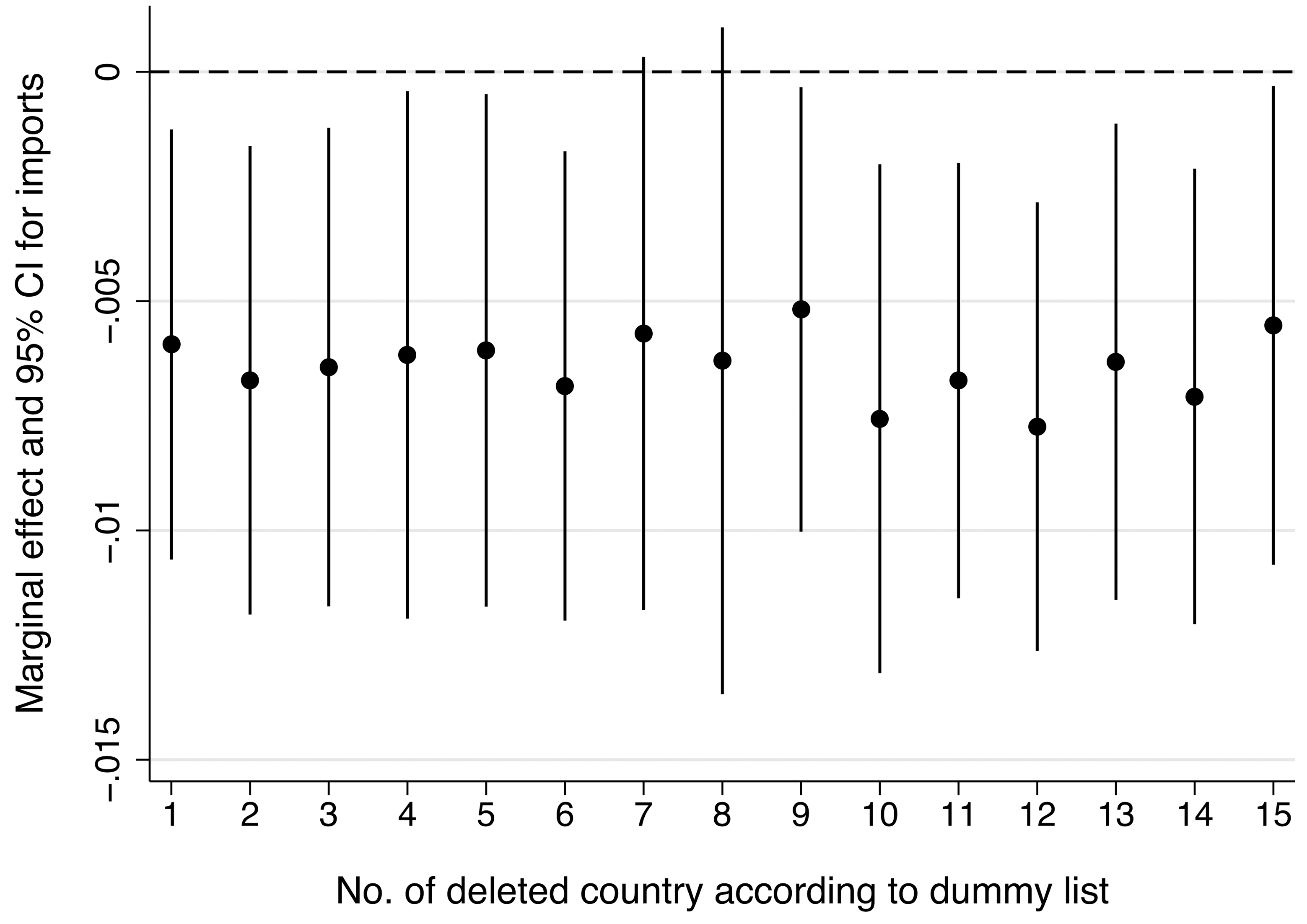

Supplement: S6 Fig — 1 Austria, 2 Belgium, 3 Cyprus, 4 Denmark, 5 Finland, 6 Germany, 7 Greece, 8 Ireland, 9 Italy, 10 Luxembourg, 11 Netherlands, 12 Portugal, 13 Spain, 14 Sweden, 15 United Kingdom. (TIF) [file pone.0212945.s013.tif]

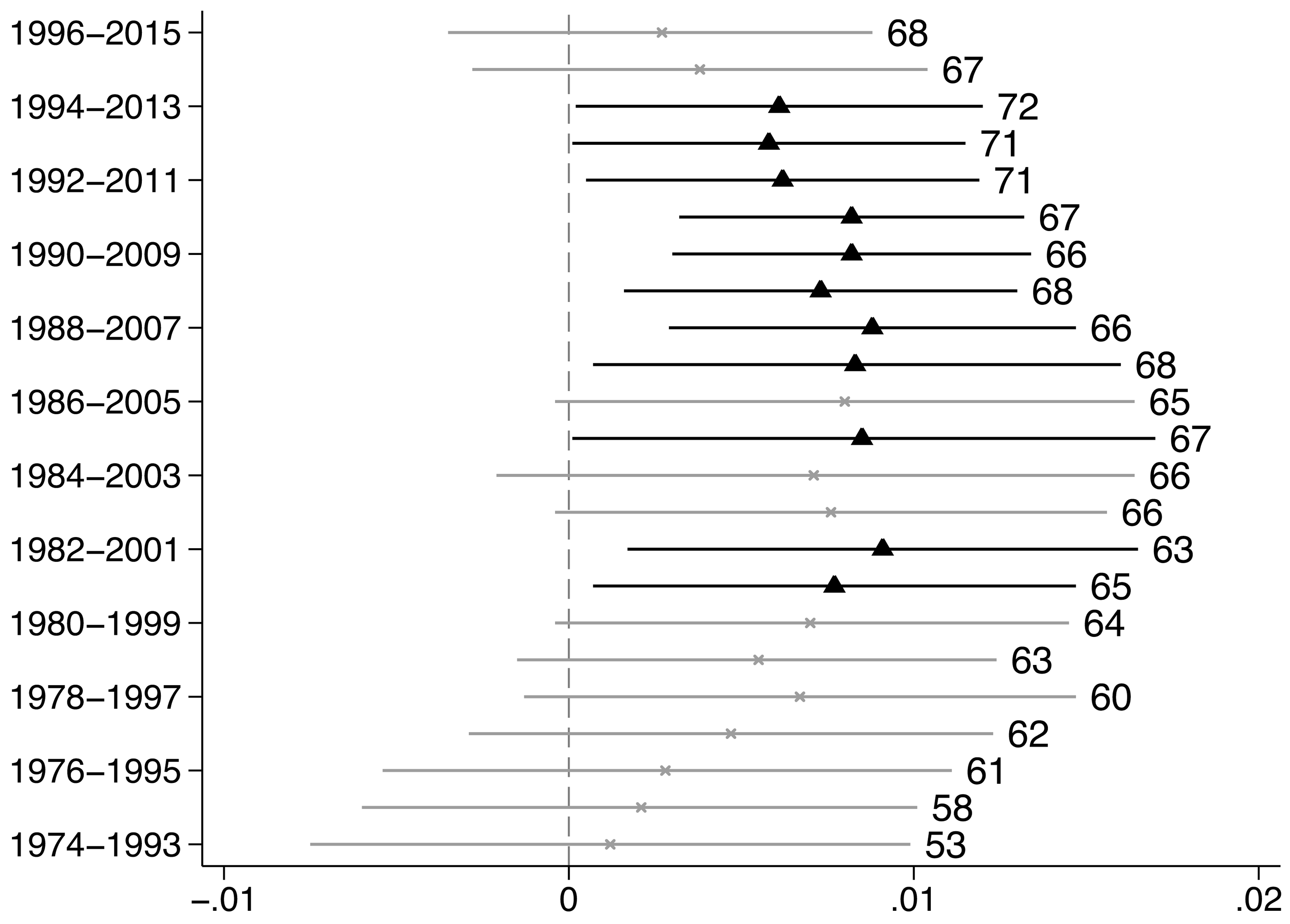

Supplement: S7 Fig — Numbers next to confidence intervals are observations per regression. We run the moving-window analysis with the trade balance variable instead of imports and exports to account for multicollinearity in the baseline model because of the correlation between imports and exports. The results confirm the theoretical arguments and findings and the estimates for trade balance display the expected development. The effects become positively significant during the second subperiod and tend to get smaller and remain significant in the third subperiod. As before, the confidence intervals are wide and the coefficient is estimated with relatively large uncertainty. (TIF) [file pone.0212945.s014.tif]

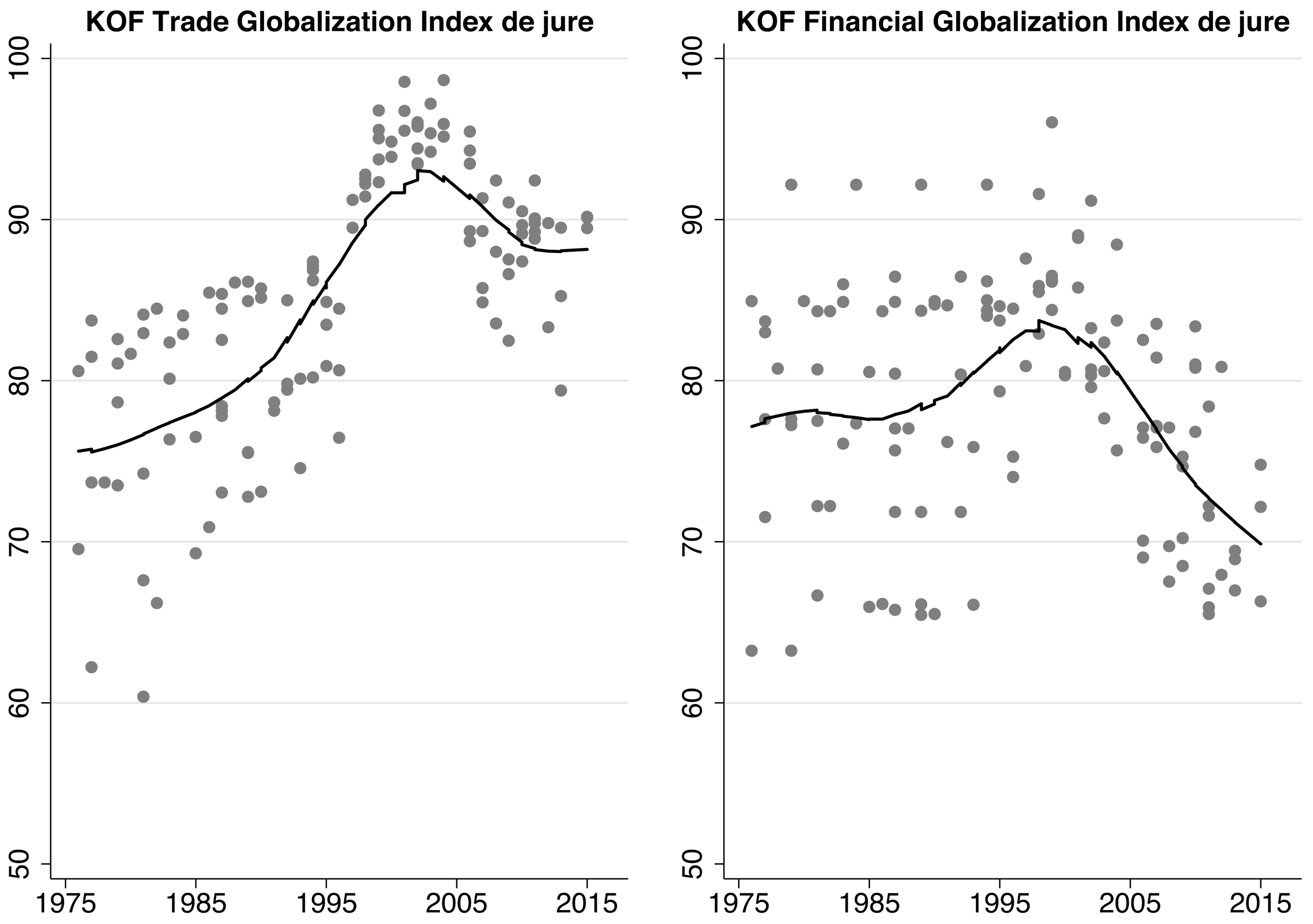

Supplement: S8 Fig — Lowess smoother, bandwith = 0.5. (TIF) [file pone.0212945.s015.tif]
