# Supplementary material for: The time-varying relationship between economic globalization and the ideological center of gravity of party systems
Source: PLoS One. 2019 Feb 27;14(2):e0212945. doi: 10.1371/journal.pone.0212945 (PMC6392286; doi:10.1371/journal.pone.0212945)
Supplement: S1 File — (PDF) [file pone.0212945.s016.pdf]

```
------------------------------------------------------------------------------------
> -----------------
      name:  <unnamed>
       log:  C:\data\sync\global\3 - PLOS\revision 2\OSF\center_of_gravity.log
  log type:  text
 opened on:   6 Feb 2019, 10:00:26

. 
. * Loading processed data. See plos_construction.do for data collection and preparati
> on
. use partysystems_complete, clear
(Rohlfing/Schaffoener: Globalization and Party Ideology)

. 
. *** Analysis
. gen basesample = cond(lr_eco_broad != . & ///
>         po_mean_l1 != . & ///
>         wdi_import_l1 != . & ///
>         wdi_export_l1 != . & ///
>         wdi_gdpgr_l1 != . & ///
>         wdi_gdpcapcur_l1, 1, 0)

. label var basesample "Base model sample w non-missing on all variables"

. 
. * We exclude Norway because of too few observations
. replace basesample = 0 if countryname=="Norway"
(1 real change made)

. keep if basesample == 1
(579 observations deleted)

. 
. * Descriptive statistics
. * Table 4
. tabstat lr_eco_broad wdi_export_l1 wdi_import_l1 tradebal_wdi_l1 wdi_fdiin_l1 ///
>         po_mean_l1 wdi_gdpgr_l1 wdi_gdpcapcur_l1, statistics(mean sd min max count)

   stats |  lr_eco~d  wdi_ex~1  wdi_im~1  tradeb~1  wd~in_l1  po_mea~1  ~dpgr_l1  wd~u
> r_l1
---------+--------------------------------------------------------------------------
> ----
    mean | -.4847193   43.5571   42.1822  1.374897  4.827869  5.342777  2.087704  2406
> 5.59
      sd |  .1810556  29.53257  23.69967  7.734388  14.29705  .4137312  2.702507  1752
> 3.07
     min | -.7856123  14.42528  16.88713 -14.20826 -3.679174  4.388316 -9.132494  2922
> .863
     max |  .1574465  189.2374  159.3568  30.35266   142.257   6.39023  9.269007  1128
> 51.5
       N |       129       129       129       129       116       129       129
>  129
------------------------------------------------------------------------------------
> ----

. quietly: tab countryname

. display as text "Sample covers " r(r) " countries"
Sample covers 15 countries
```

```
.
. * Plot for center of gravity, scatter plot
. * Fig 1
. scatter lr_eco_broad year, by(countryname, note("") graphregion(color(white))) ///
>         ms(i) c(l) lw(medthick) lc(gs0) ///
>         yline(0, lp(dash) lc(gs8)) ylabel(-1(0.5)0.5, labsize(medsmall) ///
>         angle(horizontal))  ///
>         ytitle("Center of gravity", size(small)) ///
>         xtitle("Year", size(small)) ///
>         legend(off)

. graph save center_of_gravity, replace
(note: file center_of_gravity.gph not found)
(file center_of_gravity.gph saved)

. graph export fig1.eps, replace
(note: file fig1.eps not found)
(file fig1.eps written in EPS format)

.
. * Plot of elections over time
. * S1 Fig
. quietly: sum year

. gen obsid = _n

. egen elecid = concat(edate obsid) // some elections are on same day

. destring elecid, generate (elecid_destring)
elecid: all characters numeric; elecid_destring generated as long

. bys year: egen eleccount = count(elecid_destring)

. twoway bar eleccount year, ///
>         graphregion(color(white)) ///
>         ylabel(0(1)8) ytitle("Number of elections") yscale(titlegap(*10)) ///
>         xlabel(1975(5)2016) xtitle("Year") xscale(titlegap(*10))

. graph save elec_per_year, replace
(note: file elec_per_year.gph not found)
(file elec_per_year.gph saved)

. graph export s1_fig.eps, replace
(note: file s1_fig.eps not found)
(file s1_fig.eps written in EPS format)

.
. * Plot of imports and exports
. * S2 Fig
. sort countryname edate

. scatter wdi_import wdi_export year, by(countryname, ///
>         graphregion(color(white)) note("")) ///
>         ylabel(0(50)150, labsize(small) angle(horizontal)) ///
>         ytitle("Imports/Exports to GDP", size(small)) ///
>         xtitle("Year", size(small)) ///
>         ms(i Oh) mc (black black) c(l l) lc(gs12 gs0) lw(medthick medium) ///
>         legend(label(1 "Imports (% GDP)") label(2 "Exports (% GDP)") ///
>         row(1) size(small))
```

```
. graph save impexp, replace
(note: file impexp.gph not found)
(file impexp.gph saved)

. graph export s2_fig.eps, replace
(note: file s2_fig.eps not found)
(file s2_fig.eps written in EPS format)

.
. * Setting tsset and xtset for assignment of TSCS structure
. egen panelvar_id = group(country)

. label var panelvar_id "Panel ID"

. sort country edate

. by country: gen timevar_id = _n

. label var timevar_id "Election ID per country"

. xtset panelvar_id timevar_id
       panel variable:  panelvar_id (unbalanced)
        time variable:  timevar_id, 1 to 15
                delta:  1 unit

.
. *** Regression analysis of center with controls
. * Test for first-order serial autocorrelation
. reg lr_eco_broad wdi_export_l1 wdi_import_l1 po_mean_l1 ///
>         wdi_gdpgr_l1 wdi_gdpcapcur_l1

      Source |       SS           df       MS      Number of obs   =       129
-------------+----------------------------------   F(5, 123)       =      9.43
       Model |  1.16277934         5  .232555868   Prob > F        =    0.0000
    Residual |  3.03320321       123  .024660189   R-squared       =    0.2771
-------------+----------------------------------   Adj R-squared   =    0.2477
       Total |  4.19598255       128  .032781114   Root MSE        =    .15704

----------------------------------------------------------------------------------
    lr_eco_broad |      Coef.   Std. Err.      t    P>|t|     [95% Conf. Interval]
-----------------+----------------------------------------------------------------
   wdi_export_l1 |   .0080815   .0027181     2.97   0.004     .0027011    .0134618
   wdi_import_l1 |  -.0097842   .0032364    -3.02   0.003    -.0161905   -.0033778
      po_mean_l1 |   .1202805   .0376971     3.19   0.002     .0456614    .1948996
    wdi_gdpgr_l1 |  -.0199779   .0055082    -3.63   0.000    -.0308811   -.0090747
wdi_gdpcapcur_l1 |  -4.44e-06   1.24e-06    -3.57   0.001    -6.90e-06   -1.98e-06
           _cons |  -.9181465   .1996989    -4.60   0.000    -1.313438   -.5228548
----------------------------------------------------------------------------------

. keep if e(sample)
(0 observations deleted)

. predict resid, resid

. sort country edate

. by country: generate resid_l = resid[_n-1]
(15 missing values generated)

. reg resid resid_l wdi_export_l1 wdi_import_l1 po_mean_l1

      Source |       SS           df       MS      Number of obs   =       114
-------------+----------------------------------   F(4, 109)       =      4.69
       Model |  .372192008         4  .093048002   Prob > F        =    0.0016
    Residual |  2.16450843       109  .019857876   R-squared       =    0.1467
-------------+----------------------------------   Adj R-squared   =    0.1154
       Total |  2.53670044       113  .022448676   Root MSE        =    .14092
```

```
------------------------------------------------------------------------------
       resid |      Coef.   Std. Err.      t    P>|t|     [95% Conf. Interval]
-------------+----------------------------------------------------------------
     resid_l |   .3882172   .0899972     4.31   0.000     .2098457    .5665888
wdi_export_l1 |  -.0015175   .0024303    -0.62   0.534    -.0063343    .0032994
wdi_import_l1 |   .0022708   .0030622     0.74   0.460    -.0037984      .00834
  po_mean_l1 |  -.0374981   .0347358    -1.08   0.283    -.1063434    .0313472
       _cons |   .1714743   .1788472     0.96   0.340    -.1829951    .5259437
------------------------------------------------------------------------------

. display as text "Null of no serial autocorrelation rejected"
Null of no serial autocorrelation rejected

. drop resid resid_l

.
. * Test for first-order serial autocorrelation when lagged DV is included
. reg lr_eco_broad wdi_export_l1 wdi_import_l1 po_mean_l1 ///
>         wdi_gdpgr_l1 wdi_gdpcapcur_l1 lr_eco_broad_l1

      Source |       SS           df       MS      Number of obs   =       129
-------------+----------------------------------   F(6, 122)       =     12.98
       Model |  1.63484778         6  .27247463   Prob > F        =    0.0000
    Residual |  2.56113477       122  .020992908   R-squared       =    0.3896
-------------+----------------------------------   Adj R-squared   =    0.3596
       Total |  4.19598255       128  .032781114   Root MSE        =    .14489

----------------------------------------------------------------------------------
    lr_eco_broad |      Coef.   Std. Err.      t    P>|t|     [95% Conf. Interval]
-----------------+----------------------------------------------------------------
   wdi_export_l1 |   .0056768   .0025586     2.22   0.028     .0006117    .0107418
   wdi_import_l1 |  -.0064362   .0030684    -2.10   0.038    -.0125105   -.0003619
      po_mean_l1 |    .087551   .0354595     2.47   0.015     .0173554    .1577467
    wdi_gdpgr_l1 |  -.0218497   .0050975    -4.29   0.000    -.0319407   -.0117587
wdi_gdpcapcur_l1 |  -3.59e-06   1.16e-06    -3.09   0.002    -5.88e-06   -1.29e-06
 lr_eco_broad_l1 |   .3442217   .0725892     4.74   0.000     .2005241    .4879193
           _cons |  -.6347477   .1937025    -3.28   0.001    -1.018201   -.2512943
----------------------------------------------------------------------------------

. keep if e(sample)
(0 observations deleted)

. predict resid, resid

. sort country edate

. by country: generate resid_l = resid[_n-1]
(15 missing values generated)

. reg resid resid_l wdi_export_l1 wdi_import_l1 po_mean_l1

      Source |       SS           df       MS      Number of obs   =       114
-------------+----------------------------------   F(4, 109)       =      0.56
       Model |  .043648642         4  .01091216   Prob > F        =    0.6918
    Residual |   2.1220256       109  .019468125   R-squared       =    0.0202
-------------+----------------------------------   Adj R-squared   =   -0.0158
       Total |  2.16567424       113  .019165259   Root MSE        =    .13953

------------------------------------------------------------------------------
       resid |      Coef.   Std. Err.      t    P>|t|     [95% Conf. Interval]
-------------+----------------------------------------------------------------
     resid_l |   .1162415   .0982062     1.18   0.239       -.0784    .3108829
wdi_export_l1 |  -.0011475   .0024051    -0.48   0.634    -.0059143    .0036194
wdi_import_l1 |   .0016335   .0030295     0.54   0.591    -.0043709    .0076378
  po_mean_l1 |  -.0379432   .0343504    -1.10   0.272    -.1060246    .0301382
       _cons |    .183233   .1769866     1.04   0.303    -.1675486    .5340146
------------------------------------------------------------------------------
```

```
. display as text "Null of no serial autocorrelation not rejected"
Null of no serial autocorrelation not rejected

. drop resid resid_l

.
. * Test for second-order serial autocorrelation
. reg lr_eco_broad wdi_export_l1 wdi_import_l1 po_mean_l1 ///
>         wdi_gdpgr_l1 wdi_gdpcapcur_l1

      Source |       SS           df       MS      Number of obs   =       129
-------------+----------------------------------   F(5, 123)       =      9.43
       Model |  1.16277934         5  .232555868   Prob > F        =    0.0000
    Residual |  3.03320321       123  .024660189   R-squared       =    0.2771
-------------+----------------------------------   Adj R-squared   =    0.2477
       Total |  4.19598255       128  .032781114   Root MSE        =    .15704

----------------------------------------------------------------------------------
    lr_eco_broad |      Coef.   Std. Err.      t    P>|t|     [95% Conf. Interval]
-----------------+----------------------------------------------------------------
   wdi_export_l1 |   .0080815   .0027181     2.97   0.004     .0027011    .0134618
   wdi_import_l1 |  -.0097842   .0032364    -3.02   0.003    -.0161905   -.0033778
      po_mean_l1 |   .1202805   .0376971     3.19   0.002     .0456614    .1948996
    wdi_gdpgr_l1 |  -.0199779   .0055082    -3.63   0.000    -.0308811   -.0090747
wdi_gdpcapcur_l1 |  -4.44e-06   1.24e-06    -3.57   0.001    -6.90e-06   -1.98e-06
           _cons |  -.9181465   .1996989    -4.60   0.000    -1.313438   -.5228548
----------------------------------------------------------------------------------

. keep if e(sample)
(0 observations deleted)

. predict resid, resid

. sort country edate

. by country: generate resid_l = resid[_n-1]
(15 missing values generated)

. by country: generate resid_l2 = resid[_n-2]
(30 missing values generated)

. reg resid resid_l resid_l2 wdi_export_l1 wdi_import_l1 po_mean_l1

      Source |       SS           df       MS      Number of obs   =        99
-------------+----------------------------------   F(5, 93)        =      3.56
       Model |  .378759628         5  .075751926   Prob > F        =    0.0055
    Residual |  1.98087865        93   .02129977   R-squared       =    0.1605
-------------+----------------------------------   Adj R-squared   =    0.1154
       Total |  2.35963827        98  .024077942   Root MSE        =    .14594

-------------------------------------------------------------------------------
        resid |      Coef.   Std. Err.      t    P>|t|     [95% Conf. Interval]
--------------+----------------------------------------------------------------
      resid_l |   .4502385   .1105356     4.07   0.000     .2307367    .6697403
     resid_l2 |  -.0670846   .1049011    -0.64   0.524    -.2753973    .1412282
wdi_export_l1 |   -.000369   .0028163    -0.13   0.896    -.0059616    .0052235
wdi_import_l1 |   .0008169    .003566     0.23   0.819    -.0062645    .0078983
   po_mean_l1 |  -.0395234   .0401607    -0.98   0.328    -.1192746    .0402278
        _cons |   .1938588   .2058323     0.94   0.349    -.2148833    .6026008
-------------------------------------------------------------------------------
```

```
. display as text "Null of no serial autocorrelation not rejected"
Null of no serial autocorrelation not rejected

. drop resid resid_l resid_l2

.
. * Test for second-order serial autocorrelation when lagged DV is included
. reg lr_eco_broad wdi_export_l1 wdi_import_l1 po_mean_l1 ///
>         wdi_gdpgr_l1 wdi_gdpcapcur_l1 lr_eco_broad_l1

      Source |       SS           df       MS      Number of obs   =       129
-------------+----------------------------------   F(6, 122)       =     12.98
       Model |  1.63484778         6   .27247463   Prob > F        =    0.0000
    Residual |  2.56113477       122  .020992908   R-squared       =    0.3896
-------------+----------------------------------   Adj R-squared   =    0.3596
       Total |  4.19598255       128  .032781114   Root MSE        =    .14489

----------------------------------------------------------------------------------
    lr_eco_broad |      Coef.   Std. Err.      t    P>|t|     [95% Conf. Interval]
-----------------+----------------------------------------------------------------
   wdi_export_l1 |   .0056768   .0025586     2.22   0.028     .0006117    .0107418
   wdi_import_l1 |  -.0064362   .0030684    -2.10   0.038    -.0125105   -.0003619
      po_mean_l1 |    .087551   .0354595     2.47   0.015     .0173554    .1577467
    wdi_gdpgr_l1 |  -.0218497   .0050975    -4.29   0.000    -.0319407   -.0117587
wdi_gdpcapcur_l1 |  -3.59e-06   1.16e-06    -3.09   0.002    -5.88e-06   -1.29e-06
 lr_eco_broad_l1 |   .3442217   .0725892     4.74   0.000     .2005241    .4879193
           _cons |  -.6347477   .1937025    -3.28   0.001    -1.018201   -.2512943
----------------------------------------------------------------------------------

. keep if e(sample)
(0 observations deleted)

. predict resid, resid

. sort country edate

. by country: generate resid_l = resid[_n-1]
(15 missing values generated)

. by country: generate resid_l2 = resid[_n-2]
(30 missing values generated)

. reg resid resid_l resid_l2 wdi_export_l1 wdi_import_l1 po_mean_l1

      Source |       SS           df       MS      Number of obs   =        99
-------------+----------------------------------   F(5, 93)        =      0.41
       Model |  .043885827         5  .008777165   Prob > F        =    0.8411
    Residual |  1.99301029        93  .021430218   R-squared       =    0.0215
-------------+----------------------------------   Adj R-squared   =   -0.0311
       Total |  2.03689612        98  .020784654   Root MSE        =    .14639

-------------------------------------------------------------------------------
        resid |      Coef.   Std. Err.      t    P>|t|     [95% Conf. Interval]
--------------+----------------------------------------------------------------
      resid_l |   .1113821   .1131855     0.98   0.328    -.1133818     .336146
     resid_l2 |  -.0407754   .1063406    -0.38   0.702    -.2519469     .170396
wdi_export_l1 |  -.0001091   .0028197    -0.04   0.969    -.0057084    .0054903
wdi_import_l1 |   .0003111   .0035678     0.09   0.931    -.0067739    .0073962
   po_mean_l1 |  -.0384159   .0399447    -0.96   0.339    -.1177382    .0409063
        _cons |   .1967068   .2050787     0.96   0.340    -.2105389    .6039525
-------------------------------------------------------------------------------
```

```
. display as text "Null of no serial autocorrelation not rejected"
Null of no serial autocorrelation not rejected

. drop resid resid_l resid_l2

.
. * Test for country fixed effects
. areg lr_eco_broad wdi_export_l1 wdi_import_l1 po_mean_l1 ///
>         wdi_gdpgr_l1 wdi_gdpcapcur_l1 lr_eco_broad_l1, absorb(countryname)

Linear regression, absorbing indicators         Number of obs     =        129
Absorbed variable: countryname                  No. of categories =         15
                                                F(   6,    108)   =       7.40
                                                Prob > F          =     0.0000
                                                R-squared         =     0.4501
                                                Adj R-squared     =     0.3483
                                                Root MSE          =     0.1462

------------------------------------------------------------------------------------
    lr_eco_broad |      Coef.   Std. Err.      t    P>|t|     [95% Conf. Interval]
-----------------+------------------------------------------------------------------
   wdi_export_l1 |   .0060645   .0037001     1.64   0.104    -.0012697    .0133987
   wdi_import_l1 |  -.0049146   .0047159    -1.04   0.300    -.0142622    .0044331
      po_mean_l1 |   .0639001   .0590459     1.08   0.282    -.0531391    .1809393
    wdi_gdpgr_l1 |  -.0205403   .0054625    -3.76   0.000     -.031368   -.0097125
wdi_gdpcapcur_l1 |  -4.58e-06   1.81e-06    -2.53   0.013    -8.17e-06   -9.96e-07
 lr_eco_broad_l1 |   .2848274    .084636     3.37   0.001     .1170641    .4525906
           _cons |  -.5961464   .3402635    -1.75   0.083    -1.270608    .0783148
------------------------------------------------------------------------------------
F test of absorbed indicators: F(14, 108) = 0.849             Prob > F = 0.615

. display as text "Null of no country fixed effects not rejected at .05"
Null of no country fixed effects not rejected at .05

.
. * Test for period fixed effects
. areg lr_eco_broad wdi_export_l1 wdi_import_l1 po_mean_l1 ///
>                 wdi_gdpgr_l1 wdi_gdpcapcur_l1 lr_eco_broad_l1, absorb(year)

Linear regression, absorbing indicators         Number of obs     =        129
Absorbed variable: year                         No. of categories =         38
                                                F(   6,     85)   =       6.34
                                                Prob > F          =     0.0000
                                                R-squared         =     0.6179
                                                Adj R-squared     =     0.4246
                                                Root MSE          =     0.1373

------------------------------------------------------------------------------------
    lr_eco_broad |      Coef.   Std. Err.      t    P>|t|     [95% Conf. Interval]
-----------------+------------------------------------------------------------------
   wdi_export_l1 |   .0048959   .0035673     1.37   0.174    -.0021969    .0119887
   wdi_import_l1 |  -.0054821   .0040867    -1.34   0.183    -.0136074    .0026433
      po_mean_l1 |    .073472   .0384018     1.91   0.059    -.0028811    .1498251
    wdi_gdpgr_l1 |   -.014944   .0082514    -1.81   0.074      -.03135     .001462
wdi_gdpcapcur_l1 |  -2.76e-06   2.24e-06    -1.23   0.222    -7.21e-06    1.70e-06
 lr_eco_broad_l1 |    .391474   .0823862     4.75   0.000     .2276682    .5552798
           _cons |  -.5780434   .2108386    -2.74   0.007    -.9972471   -.1588397
------------------------------------------------------------------------------------
F test of absorbed indicators: F(37, 85) = 1.372              Prob > F = 0.117
```

```
. display as text "Null of no period fixed effects not rejected at .05"
Null of no period fixed effects not rejected at .05

.
. * Baseline: OLS with two-way clustered standard errors and lagged DV
. xtset panelvar_id timevar_id
       panel variable:  panelvar_id (unbalanced)
        time variable:  timevar_id, 1 to 15
                delta:  1 unit

. // requires cluster2.ado file (see README file and supplementary material)
. cluster2 lr_eco_broad wdi_export_l1 wdi_import_l1 po_mean_l1 ///
>         wdi_gdpgr_l1 wdi_gdpcapcur_l1 lr_eco_broad_l1, fcluster(countryname) ///
>         tcluster(year)

Linear regression with 2D clustered SEs                 Number of obs =     129
                                                        F(  6,   124) =   14.69
                                                        Prob > F      =  0.0000
Number of clusters (countryname) =    15                R-squared     =  0.3896
Number of clusters (year) =      38                     Root MSE      =  0.1449
------------------------------------------------------------------------------
    lr_eco_broad |      Coef.   Std. Err.      t    P>|t|     [95% Conf. Interval]
-----------------+------------------------------------------------------------
   wdi_export_l1 |   .0056768   .0021612     2.63   0.010     .0013992    .0099543
   wdi_import_l1 |  -.0064362   .0026401    -2.44   0.016    -.0116618   -.0012106
      po_mean_l1 |    .087551   .0349771     2.50   0.014     .0183216    .1567805
    wdi_gdpgr_l1 |  -.0218497   .0032979    -6.63   0.000    -.0283771   -.0153223
wdi_gdpcapcur_l1 |  -3.59e-06   1.12e-06    -3.20   0.002    -5.81e-06   -1.37e-06
 lr_eco_broad_l1 |   .3442217   .0531408     6.48   0.000     .2390413    .4494022
           _cons |  -.6347477    .193572    -3.28   0.001    -1.017881   -.2516144
------------------------------------------------------------------------------

     SE clustered by countryname and year (multiple obs per countryname-year)


. display e(r2_a)
.35960358

. est sto twowayclusterDV

.
. * OLS with standard errors clustered by country and lagged DV
. reg lr_eco_broad wdi_export_l1 wdi_import_l1 po_mean_l1 ///
>         wdi_gdpgr_l1 wdi_gdpcapcur_l1 lr_eco_broad_l1, cluster(countryname)

Linear regression                               Number of obs     =        129
                                                F(6, 14)          =      70.67
                                                Prob > F          =     0.0000
                                                R-squared         =     0.3896
                                                Root MSE          =     .14489

                              (Std. Err. adjusted for 15 clusters in countryname)
------------------------------------------------------------------------------
                 |               Robust
    lr_eco_broad |      Coef.   Std. Err.      t    P>|t|     [95% Conf. Interval]
-----------------+------------------------------------------------------------
   wdi_export_l1 |   .0056768   .0018015     3.15   0.007      .001813    .0095405
   wdi_import_l1 |  -.0064362   .0022209    -2.90   0.012    -.0111995    -.001673
      po_mean_l1 |    .087551   .0365322     2.40   0.031     .0091972    .1659049
    wdi_gdpgr_l1 |  -.0218497   .0035172    -6.21   0.000    -.0293932   -.0143061
wdi_gdpcapcur_l1 |  -3.59e-06   1.02e-06    -3.51   0.003    -5.78e-06   -1.40e-06
 lr_eco_broad_l1 |   .3442217   .0592743     5.81   0.000      .217091    .4713524
           _cons |  -.6347477   .2036771    -3.12   0.008    -1.071592   -.1979037
------------------------------------------------------------------------------
```

```
. display e(r2_a)
.35960358

. est sto countryclusterDV

.
. * OLS with standard errors clustered by period and lagged DV
. reg lr_eco_broad wdi_export_l1 wdi_import_l1 po_mean_l1 ///
>         wdi_gdpgr_l1 wdi_gdpcapcur_l1 lr_eco_broad_l1, cluster(year)

Linear regression                               Number of obs     =        129
                                                F(6, 37)          =       9.80
                                                Prob > F          =     0.0000
                                                R-squared         =     0.3896
                                                Root MSE          =     .14489

                                  (Std. Err. adjusted for 38 clusters in year)
----------------------------------------------------------------------------------
                 |               Robust
    lr_eco_broad |      Coef.   Std. Err.      t    P>|t|     [95% Conf. Interval]
-----------------+----------------------------------------------------------------
   wdi_export_l1 |   .0056768   .0028341     2.00   0.053    -.0000657    .0114192
   wdi_import_l1 |  -.0064362    .003518    -1.83   0.075    -.0135644     .000692
      po_mean_l1 |    .087551   .0341742     2.56   0.015     .0183076    .1567945
    wdi_gdpgr_l1 |  -.0218497   .0045202    -4.83   0.000    -.0310085   -.0126909
wdi_gdpcapcur_l1 |  -3.59e-06   1.07e-06    -3.34   0.002    -5.76e-06   -1.41e-06
 lr_eco_broad_l1 |   .3442217   .0676036     5.09   0.000     .2072439    .4811995
           _cons |  -.6347477   .1868885    -3.40   0.002     -1.01342   -.2560756
----------------------------------------------------------------------------------

. display e(r2_a)
.35960358

. est sto periodclusterDV

.
. * OLS without clustering and lagged DV
. reg lr_eco_broad wdi_export_l1 wdi_import_l1 po_mean_l1 ///
>         wdi_gdpgr_l1 wdi_gdpcapcur_l1 lr_eco_broad_l1

      Source |       SS           df       MS      Number of obs   =       129
-------------+----------------------------------   F(6, 122)       =     12.98
       Model |  1.63484778         6   .27247463   Prob > F        =    0.0000
    Residual |  2.56113477       122  .020992908   R-squared       =    0.3896
-------------+----------------------------------   Adj R-squared   =    0.3596
       Total |  4.19598255       128  .032781114   Root MSE        =    .14489

----------------------------------------------------------------------------------
    lr_eco_broad |      Coef.   Std. Err.      t    P>|t|     [95% Conf. Interval]
-----------------+----------------------------------------------------------------
   wdi_export_l1 |   .0056768   .0025586     2.22   0.028     .0006117    .0107418
   wdi_import_l1 |  -.0064362   .0030684    -2.10   0.038    -.0125105   -.0003619
      po_mean_l1 |    .087551   .0354595     2.47   0.015     .0173554    .1577467
    wdi_gdpgr_l1 |  -.0218497   .0050975    -4.29   0.000    -.0319407   -.0117587
wdi_gdpcapcur_l1 |  -3.59e-06   1.16e-06    -3.09   0.002    -5.88e-06   -1.29e-06
 lr_eco_broad_l1 |   .3442217   .0725892     4.74   0.000     .2005241    .4879193
           _cons |  -.6347477   .1937025    -3.28   0.001    -1.018201   -.2512943
----------------------------------------------------------------------------------

. display e(r2_a)
.35960358
```

```
. est sto olsDV

.
. *** INTERNAL: clustering table for response letter
. esttab twowayclusterDV countryclusterDV periodclusterDV olsDV using cluster_table.rt
> f, replace ///
>         cells(b(star fmt(%9.4f)) se(par)) stats(r2_a N, fmt(2 0)) varwidth(10) ///
>         modelwidth(9) order(wdi_import_l1 wdi_export_l1 po_mean_l1 ///
>         wdi_gdpgr_l1 wdi_gdpcapcur_l1 lr_eco_broad_l1) ///
>         varlabels(wdi_import_l1 "Imports" wdi_export_l1 "Exports" ///
>         po_mean_l1 "Median voter" wdi_gdpgr_l1 "GDP growth" ///
>         wdi_gdpcapcur_l1 "GDP/capita" _cons "Constant" ///
>         lr_eco_broad_l1 "Lagged DV") ///
>         mlabels("two-way cluster" "country cluster" "period cluster" "no clustering"
> ) ///
>         addnote("Standard errors in parentheses; two-sided tests; p < .05 *; p < .01
>  **; p < .001 ***
> ") ///
>         nonumbers nolines
(note: file cluster_table.rtf not found)
(output written to cluster_table.rtf)

.
. * Two-way with FDI
. cluster2 lr_eco_broad wdi_export_l1 wdi_import_l1 wdi_fdiin_l1 po_mean_l1 ///
>         wdi_gdpgr_l1 wdi_gdpcapcur_l1 lr_eco_broad_l1, ///
>         fcluster(countryname) tcluster(year)

Linear regression with 2D clustered SEs              Number of obs =     116
                                                     F(  7,   111) =   10.86
                                                     Prob > F      =  0.0000
Number of clusters (countryname) =    15             R-squared     =  0.3703
Number of clusters (year) =     37                   Root MSE      =  0.1472
------------------------------------------------------------------------------
    lr_eco_broad |      Coef.   Std. Err.      t    P>|t|     [95% Conf. Interval]
-----------------+------------------------------------------------------------
   wdi_export_l1 |   .0064192   .0022359     2.87   0.005     .0019886    .0108498
   wdi_import_l1 |  -.0073428   .0026017    -2.82   0.006    -.0124982   -.0021874
    wdi_fdiin_l1 |   .0007719   .0005433     1.42   0.158    -.0003046    .0018485
      po_mean_l1 |   .0780226   .0313343     2.49   0.014     .0159316    .1401136
    wdi_gdpgr_l1 |   -.021441   .0037214    -5.76   0.000    -.0288152   -.0140668
wdi_gdpcapcur_l1 |  -4.02e-06   1.24e-06    -3.25   0.002    -6.47e-06   -1.56e-06
 lr_eco_broad_l1 |   .3176889   .0595204     5.34   0.000     .1997452    .4356326
           _cons |  -.5845746   .1785875    -3.27   0.001    -.9384577   -.2306915
------------------------------------------------------------------------------

     SE clustered by countryname and year (multiple obs per countryname-year)


. display e(r2_a)
.32952414

. est sto twowayfdi

.
. * Control for panel-correct standard errors (with lagged DV)
. /* We use one-way clustering by country to be able to predict residuals */
. reg lr_eco_broad wdi_export_l1 wdi_import_l1 po_mean_l1 ///
>         wdi_gdpgr_l1 wdi_gdpcapcur_l1 lr_eco_broad_l1, ///
>         cluster(countryname)

Linear regression                               Number of obs     =        129
                                                F(6, 14)          =      70.67
                                                Prob > F          =     0.0000
                                                R-squared         =     0.3896
                                                Root MSE          =     .14489
```

```
                               (Std. Err. adjusted for 15 clusters in countryname)
----------------------------------------------------------------------------------
                 |               Robust
    lr_eco_broad |      Coef.   Std. Err.      t    P>|t|     [95% Conf. Interval]
-----------------+----------------------------------------------------------------
   wdi_export_l1 |   .0056768   .0018015     3.15   0.007      .001813    .0095405
   wdi_import_l1 |  -.0064362   .0022209    -2.90   0.012    -.0111995    -.001673
      po_mean_l1 |    .087551   .0365322     2.40   0.031     .0091972    .1659049
    wdi_gdpgr_l1 |  -.0218497   .0035172    -6.21   0.000    -.0293932   -.0143061
wdi_gdpcapcur_l1 |  -3.59e-06   1.02e-06    -3.51   0.003    -5.78e-06   -1.40e-06
 lr_eco_broad_l1 |   .3442217   .0592743     5.81   0.000      .217091    .4713524
           _cons |  -.6347477   .2036771    -3.12   0.008    -1.071592   -.1979037
----------------------------------------------------------------------------------

. predict resid, resid

. robvar resid, by(countryname)

                             |        Summary of Residuals
         English country name |        Mean   Std. Dev.       Freq.
------------------------------+------------------------------------
                      Austria |  -.14821657   .06114611           4
                      Belgium |   .00235622   .13767632          11
                       Cyprus |  -.03223224    .1443641           2
                      Denmark |   .04589078   .19774856          12
                      Finland |   .00888126    .1411411           5
                      Germany |  -.04082236   .15803408          10
                       Greece |  -.00184241    .1099729          15
                      Ireland |   .02110633   .14259054          11
                        Italy |   .06347802   .18844333          11
                   Luxembourg |  -.00543528   .09340446           8
                  Netherlands |  -.02574308   .09287503          12
                     Portugal |   .02120511   .20437042           7
                        Spain |  -.01908336   .05408126           9
                       Sweden |  -.06900357   .14341611           4
               United Kingdom |   .02418458    .1537906           8
------------------------------+------------------------------------
                        Total |  -9.927e-12    .1414527         129

W0  =  1.38795407   df(14, 114)     Pr > F = 0.17022047

W50 =  0.92579084   df(14, 114)     Pr > F = 0.53371177

W10 =  1.32874002   df(14, 114)     Pr > F = 0.20149201

. // Null of equal variance of residuals not rejected at .05
. drop resid

. * Panel-correct standard errors with lagged DV (clustering not possible)
. xtpcse lr_eco_broad wdi_export_l1 wdi_import_l1 po_mean_l1 ///
>         wdi_gdpgr_l1 wdi_gdpcapcur_l1 lr_eco_broad_l1, p

Linear regression, correlated panels corrected standard errors (PCSEs)

Group variable:   panelvar_id                   Number of obs     =        129
Time variable:    timevar_id                    Number of groups  =         15
Panels:           correlated (unbalanced)       Obs per group:
Autocorrelation:  no autocorrelation                          min =          2
Sigma computed by pairwise selection                          avg =        8.6
                                                              max =         15
Estimated covariances      =       120          R-squared         =     0.3896
Estimated autocorrelations =         0          Wald chi2(6)      =      53.69
Estimated coefficients     =         7          Prob > chi2       =     0.0000
```

```
------------------------------------------------------------------------------
                 |           Panel-corrected
    lr_eco_broad |      Coef.   Std. Err.      z    P>|z|     [95% Conf. Interval]
-----------------+----------------------------------------------------------------
   wdi_export_l1 |   .0056768   .0017209     3.30   0.001     .0023038    .0090498
   wdi_import_l1 |  -.0064362   .0023137    -2.78   0.005     -.010971   -.0019015
      po_mean_l1 |    .087551   .0429697     2.04   0.042      .003332    .1717701
    wdi_gdpgr_l1 |  -.0218497   .0043223    -5.06   0.000    -.0303212   -.0133782
wdi_gdpcapcur_l1 |  -3.59e-06   1.17e-06    -3.08   0.002    -5.87e-06   -1.30e-06
 lr_eco_broad_l1 |   .3442217   .1011411     3.40   0.001     .1459889    .5424545
           _cons |  -.6347477   .2219833    -2.86   0.004    -1.069827   -.1996684
------------------------------------------------------------------------------

. est sto pcse

.
. * Estimating model with trade balance
. quietly: reg lr_eco_broad wdi_export_l1 wdi_import_l1 po_mean_l1 ///
>         wdi_gdpgr_l1 wdi_gdpcapcur_l1 lr_eco_broad_l1, cluster(countryname)

. vif

    Variable |       VIF       1/VIF
-------------+----------------------
wdi_export~1 |     34.81    0.028724
wdi_import~1 |     32.24    0.031013
wdi_gd~ur_l1 |      2.52    0.397199
  po_mean_l1 |      1.31    0.762010
lr_eco_bro~1 |      1.21    0.823064
wdi_gdpgr_l1 |      1.16    0.864206
-------------+----------------------
    Mean VIF |     12.21

. display as text "VIF for imports and exports above 10"
VIF for imports and exports above 10

. cluster2 lr_eco_broad tradebal_wdi_l1 po_mean_l1 wdi_gdpgr_l1 ///
>         wdi_gdpcapcur_l1 lr_eco_broad_l1, ///
>         fcluster(countryname) tcluster(year)

Linear regression with 2D clustered SEs                Number of obs =     129
                                                       F(  5,   124) =   18.07
                                                       Prob > F      =  0.0000
Number of clusters (countryname) =    15               R-squared     =  0.3859
Number of clusters (year) =      38                    Root MSE      =  0.1447
------------------------------------------------------------------------------
    lr_eco_broad |      Coef.   Std. Err.      t    P>|t|     [95% Conf. Interval]
-----------------+----------------------------------------------------------------
 tradebal_wdi_l1 |   .0046506   .0019676     2.36   0.020     .0007562     .008545
      po_mean_l1 |   .0755837   .0381416     1.98   0.050     .0000907    .1510766
    wdi_gdpgr_l1 |  -.0224096   .0033014    -6.79   0.000     -.028944   -.0158753
wdi_gdpcapcur_l1 |  -3.98e-06   1.06e-06    -3.77   0.000    -6.07e-06   -1.89e-06
 lr_eco_broad_l1 |   .3583743   .0569677     6.29   0.000     .2456192    .4711294
           _cons |   -.584189   .2149946    -2.72   0.008    -1.009723   -.1586545
------------------------------------------------------------------------------

     SE clustered by countryname and year (multiple obs per countryname-year)


. est sto tradebal
```

```
. quietly: reg lr_eco_broad tradebal_wdi_l1 po_mean_l1 wdi_gdpgr_l1 ///
>         wdi_gdpcapcur_l1 lr_eco_broad_l1, cluster(countryname)

. vif

    Variable |       VIF       1/VIF  
-------------+----------------------
wdi_gd~ur_l1 |      2.13    0.469236
tradebal_w~1 |      1.87    0.534243
lr_eco_bro~1 |      1.15    0.867358
wdi_gdpgr_l1 |      1.14    0.878446
  po_mean_l1 |      1.11    0.899678
-------------+----------------------
    Mean VIF |      1.48

. 
. * Summarizing baseline results
. // requires ado-file esttab (see README file)
. * Table 5
. esttab twowayclusterDV twowayfdi pcse ///
>         tradebal using reg_table.rtf, replace ///
>         cells(b(star fmt(%9.4f)) se(par)) stats(r2 N, fmt(2 0)) varwidth(10) ///
>         modelwidth(9) order(wdi_import_l1 wdi_export_l1 wdi_fdiin_l1 po_mean_l1 ///
>         wdi_gdpgr_l1 wdi_gdpcapcur_l1 lr_eco_broad_l1 tradebal_wdi_l1 _cons) ///
>         varlabels(wdi_import_l1 "Imports" wdi_export_l1 "Exports" ///
>         wdi_fdiin_l1 "FDI" po_mean_l1 "Median voter" wdi_gdpgr_l1 "GDP growth" ///
>         wdi_gdpcapcur_l1 "GDP/capita" lr_eco_broad_l1 "Lagged DV" ///
>         _cons "Constant" tradebal_wdi_l1 "Trade balance") ///
>         mlabels("two-way cluster+DV" "country cluster+DV" "year cluster+DV" "PCSE" "
> FDI" "trade balan
> ce") ///
>         addnote("Standard errors in parentheses; two-sided tests; p < .05 *; p < .01
>  **; p < .001 ***
> ") ///
>         nonumbers nolines
(note: file reg_table.rtf not found)
(output written to reg_table.rtf)

. // r2 instead of ar2 because the latter is not part of one regression
. 
. 
. * Visualization of results
. * Fig 2
. /* We use one-way clustering by country to be able to use margins */
. quietly: reg lr_eco_broad wdi_export_l1 wdi_import_l1 po_mean_l1 ///
>         wdi_gdpgr_l1 wdi_gdpcapcur_l1 lr_eco_broad_l1, cluster(countryname)

. margins, dydx(wdi_export_l1 wdi_import_l1)

Average marginal effects                        Number of obs     =        129
Model VCE    : Robust

Expression   : Linear prediction, predict()
dy/dx w.r.t. : wdi_export_l1 wdi_import_l1

------------------------------------------------------------------------------
             |            Delta-method
             |      dy/dx   Std. Err.      t    P>|t|     [95% Conf. Interval]
-------------+----------------------------------------------------------------
wdi_export_l1 |   .0056768   .0018015     3.15   0.007      .001813    .0095405
wdi_import_l1 |  -.0064362   .0022209    -2.90   0.012    -.0111995    -.001673
------------------------------------------------------------------------------
```

```
. marginsplot, xtitle("") ytitle("") title("") ///
>         graphregion(color(white)) ///
>         xlabel(1 "Exports" 2 "Imports", labsize(medsmall)) ///
>         yline(0, lp(dash) lc(gs0)) ///
>         plotopts(connect(i) mcolor(gs0)) ciopts(lcolor(gs0)) ///
>         xscale(range(0.5 2.5))

  Variables that uniquely identify margins: _deriv

. graph save basemodel, replace
(note: file basemodel.gph not found)
(file basemodel.gph saved)

. graph export fig2.eps, replace
(note: file fig2.eps not found)
(file fig2.eps written in EPS format)

.
.
. *** Baseline model with KOF Indices
. * De facto economic globalization
. xtset panelvar_id timevar_id
       panel variable:  panelvar_id (unbalanced)
        time variable:  timevar_id, 1 to 15
                delta:  1 unit

. cluster2 lr_eco_broad kofecgidf_l1 po_mean_l1 wdi_gdpgr_l1 ///
>         wdi_gdpcapcur_l1 lr_eco_broad_l1, fcluster(countryname) tcluster(year)

Linear regression with 2D clustered SEs                Number of obs =     129
                                                       F(  5,   124) =   16.96
                                                       Prob > F      =  0.0000
Number of clusters (countryname) =    15               R-squared     =  0.3720
Number of clusters (year) =      38                    Root MSE      =  0.1464
------------------------------------------------------------------------------
    lr_eco_broad |      Coef.   Std. Err.      t    P>|t|     [95% Conf. Interval]
-----------------+------------------------------------------------------------
    kofecgidf_l1 |  -.0010487   .0008122    -1.29   0.199    -.0026562    .0005588
      po_mean_l1 |   .0946707   .0476798     1.99   0.049      .000299    .1890424
    wdi_gdpgr_l1 |  -.0194309   .0037725    -5.15   0.000    -.0268977   -.0119641
wdi_gdpcapcur_l1 |  -1.80e-06   6.71e-07    -2.68   0.008    -3.13e-06   -4.71e-07
 lr_eco_broad_l1 |    .360579   .0560973     6.43   0.000     .2495467    .4716113
           _cons |  -.6703074   .2392834    -2.80   0.006    -1.143916   -.1966986
------------------------------------------------------------------------------

     SE clustered by countryname and year (multiple obs per countryname-year)


. display e(r2_a)
.34648741

. est sto kofec

.
. * Subdimension de facto trade globalization
. cluster2 lr_eco_broad koftrgidf_l1 po_mean_l1 wdi_gdpgr_l1 ///
>         wdi_gdpcapcur_l1 lr_eco_broad_l1, fcluster(countryname) tcluster(year)

Linear regression with 2D clustered SEs                Number of obs =     129
                                                       F(  5,   124) =   16.97
                                                       Prob > F      =  0.0000
Number of clusters (countryname) =    15               R-squared     =  0.3706
Number of clusters (year) =      38                    Root MSE      =  0.1465
------------------------------------------------------------------------------
    lr_eco_broad |      Coef.   Std. Err.      t    P>|t|     [95% Conf. Interval]
-----------------+------------------------------------------------------------
    koftrgidf_l1 |  -.0008763   .0006772    -1.29   0.198    -.0022167    .0004641
      po_mean_l1 |     .09498   .0459356     2.07   0.041     .0040605    .1858995
    wdi_gdpgr_l1 |  -.0193731   .0038828    -4.99   0.000    -.0270582    -.011688
wdi_gdpcapcur_l1 |  -2.02e-06   7.25e-07    -2.79   0.006    -3.46e-06   -5.85e-07
 lr_eco_broad_l1 |    .361153   .0538827     6.70   0.000     .2545041     .467802
           _cons |  -.6842538   .2420279    -2.83   0.005    -1.163295   -.2052128
```

```
--------------------------------------------------------------------------------

     SE clustered by countryname and year (multiple obs per countryname-year)


. display e(r2_a)
.34503467

. est sto koftr

.
. * Subdimension de facto financial globalization
. cluster2 lr_eco_broad koffigidf_l1 po_mean_l1 wdi_gdpgr_l1 ///
>         wdi_gdpcapcur_l1 lr_eco_broad_l1, fcluster(countryname) tcluster(year)

Linear regression with 2D clustered SEs                Number of obs =     129
                                                       F(  5,   124) =   17.07
                                                       Prob > F      =  0.0000
Number of clusters (countryname) =    15               R-squared     =  0.3713
Number of clusters (year) =      38                    Root MSE      =  0.1465
--------------------------------------------------------------------------------
    lr_eco_broad |      Coef.   Std. Err.      t    P>|t|     [95% Conf. Interval]
-----------------+--------------------------------------------------------------
    koffigidf_l1 |  -.0009074   .0007621    -1.19   0.236    -.0024158    .0006011
      po_mean_l1 |   .0889995   .0461673     1.93   0.056    -.0023785    .1803776
    wdi_gdpgr_l1 |  -.0196622   .0037495    -5.24   0.000    -.0270835    -.012241
wdi_gdpcapcur_l1 |  -1.79e-06   6.22e-07    -2.88   0.005    -3.02e-06   -5.57e-07
 lr_eco_broad_l1 |    .364094   .0569525     6.39   0.000     .2513691    .4768189
           _cons |   -.640253   .2285156    -2.80   0.006    -1.092549   -.1879566
--------------------------------------------------------------------------------

     SE clustered by countryname and year (multiple obs per countryname-year)


. display e(r2_a)
.34570532

. est sto koffi

.
. * Subdimensions trade and financial globalization
. cluster2 lr_eco_broad koftrgidf_l1 koffigidf_l1 po_mean_l1 wdi_gdpgr_l1 ///
>         wdi_gdpcapcur_l1 lr_eco_broad_l1, fcluster(countryname) tcluster(year)

Linear regression with 2D clustered SEs                Number of obs =     129
                                                       F(  6,   124) =   14.59
                                                       Prob > F      =  0.0000
Number of clusters (countryname) =    15               R-squared     =  0.3720
Number of clusters (year) =      38                    Root MSE      =  0.1470
--------------------------------------------------------------------------------
    lr_eco_broad |      Coef.   Std. Err.      t    P>|t|     [95% Conf. Interval]
-----------------+--------------------------------------------------------------
    koftrgidf_l1 |  -.0004496   .0009402    -0.48   0.633    -.0023105    .0014112
    koffigidf_l1 |  -.0005975   .0010193    -0.59   0.559    -.0026151      .00142
      po_mean_l1 |    .094106   .0451601     2.08   0.039     .0047214    .1834905
    wdi_gdpgr_l1 |  -.0194571   .0039196    -4.96   0.000     -.027215   -.0116992
wdi_gdpcapcur_l1 |  -1.78e-06   6.41e-07    -2.78   0.006    -3.05e-06   -5.14e-07
 lr_eco_broad_l1 |   .3608757   .0563957     6.40   0.000     .2492529    .4724986
           _cons |  -.6664582   .2235229    -2.98   0.003    -1.108873   -.2240439
--------------------------------------------------------------------------------

     SE clustered by countryname and year (multiple obs per countryname-year)
```

```
. display e(r2_a)
.34115711

. est sto koftrfi

.
. * Summarizing KOF results for economic globalization
. * S5 Table
. esttab kofec koftr koffi koftrfi using kof_econglob.rtf, replace ///
>         cells(b(star fmt(%9.4f)) se(par)) stats(r2_a N, fmt(2 0)) varwidth(10) ///
>         modelwidth(9) order(kofecgidf_l1 koftrgidf_l1 koffigidf_l1 po_mean_l1 ///
>         wdi_gdpgr_l1 wdi_gdpcapcur_l1 _cons) ///
>         varlabels(kofecgidf_l1 "Economic Globalization" koftrgidf_l1 "Trade Globaliz
> ation" ///
>         koffigidf_l1 "Financial Globalization" ///
>         po_mean_l1 "Median voter" wdi_gdpgr_l1 "GDP growth" ///
>         wdi_gdpcapcur_l1 "GDP/capita" lr_eco_broad_l1 "Lagged DV" _cons "Constant")
> ///
>         mlabels("Economic" "Trade" "Financial" "Trade and Financial") ///
>         addnote("Standard errors in parentheses; two-sided tests; p < .05 *; p < .01
>  **; p < .001 ***
> ") ///
>         nonumbers nolines
(note: file kof_econglob.rtf not found)
(output written to kof_econglob.rtf)

.
. ** Test for political and social globalization effects
. * Dimension de facto social globalization
. cluster2 lr_eco_broad kofsogidf_l1 po_mean_l1 wdi_gdpgr_l1 ///
>         wdi_gdpcapcur_l1 lr_eco_broad_l1, fcluster(countryname) tcluster(year)

Linear regression with 2D clustered SEs                Number of obs =     129
                                                       F(  5,   124) =   18.89
                                                       Prob > F      =  0.0000
Number of clusters (countryname) =    15               R-squared     =  0.3842
Number of clusters (year) =      38                    Root MSE      =  0.1449
------------------------------------------------------------------------------
    lr_eco_broad |      Coef.   Std. Err.      t    P>|t|     [95% Conf. Interval]
-----------------+------------------------------------------------------------
    kofsogidf_l1 |  -.0043638   .0026907    -1.62   0.107    -.0096894    .0009619
      po_mean_l1 |   .1030831   .0404779     2.55   0.012     .0229661    .1832002
    wdi_gdpgr_l1 |  -.0189497    .004194    -4.52   0.000    -.0272508   -.0106486
wdi_gdpcapcur_l1 |  -9.61e-07   1.01e-06    -0.95   0.342    -2.96e-06    1.03e-06
 lr_eco_broad_l1 |   .3890153   .0483854     8.04   0.000     .2932471    .4847836
           _cons |  -.4654968   .1765107    -2.64   0.009    -.8148608   -.1161327
------------------------------------------------------------------------------

     SE clustered by countryname and year (multiple obs per countryname-year)


. display e(r2_a)
.35914232

. est sto kofsocdf

.
. * Dimension de facto political globalization
. cluster2 lr_eco_broad kofpogidf_l1 po_mean_l1 wdi_gdpgr_l1 ///
>                 wdi_gdpcapcur_l1 lr_eco_broad_l1, fcluster(countryname) tcluster(yea
> r)

Linear regression with 2D clustered SEs                Number of obs =     129
                                                       F(  5,   124) =   16.13
                                                       Prob > F      =  0.0000
Number of clusters (countryname) =    15               R-squared     =  0.3687
Number of clusters (year) =      38                    Root MSE      =  0.1468
------------------------------------------------------------------------------
    lr_eco_broad |      Coef.   Std. Err.      t    P>|t|     [95% Conf. Interval]
-----------------+------------------------------------------------------------
    kofpogidf_l1 |   .0009969    .000981     1.02   0.311    -.0009447    .0029385
      po_mean_l1 |   .0848361   .0415974     2.04   0.044     .0025033     .167169
```

```
    wdi_gdpgr_l1 |  -.0188329   .0040161    -4.69   0.000    -.0267818   -.0108841
wdi_gdpcapcur_l1 |  -2.46e-06   7.83e-07    -3.14   0.002    -4.01e-06   -9.11e-07
 lr_eco_broad_l1 |   .3625522   .0563597     6.43   0.000     .2510006    .4741038
           _cons |  -.7548693   .2461117    -3.07   0.003    -1.241993   -.2677452
------------------------------------------------------------------------------

     SE clustered by countryname and year (multiple obs per countryname-year)


. display e(r2_a)
.34303892

. est sto kofpoldf

.
. * Dimensions de facto economic, political and social globalization
. cluster2 lr_eco_broad kofecgidf_l1 kofsogidf_l1 kofpogidf_l1 po_mean_l1 ///
>         wdi_gdpgr_l1 wdi_gdpcapcur_l1 lr_eco_broad_l1, ///
>         fcluster(countryname) tcluster(year)

Linear regression with 2D clustered SEs                Number of obs =     129
                                                       F(  7,   124) =   13.50
                                                       Prob > F      =  0.0000
Number of clusters (countryname) =    15               R-squared     =  0.3905
Number of clusters (year) =      38                    Root MSE      =  0.1454
------------------------------------------------------------------------------
    lr_eco_broad |      Coef.   Std. Err.      t    P>|t|     [95% Conf. Interval]
-----------------+------------------------------------------------------------
    kofecgidf_l1 |  -.0004751   .0010386    -0.46   0.648    -.0025308    .0015806
    kofsogidf_l1 |   -.003953   .0031703    -1.25   0.215     -.010228     .002322
    kofpogidf_l1 |   .0012492   .0010184     1.23   0.222    -.0007664    .0032648
      po_mean_l1 |   .1182448   .0470619     2.51   0.013     .0250962    .2113934
    wdi_gdpgr_l1 |  -.0173391   .0041256    -4.20   0.000    -.0255048   -.0091734
wdi_gdpcapcur_l1 |  -7.23e-07   9.61e-07    -0.75   0.453    -2.63e-06    1.18e-06
 lr_eco_broad_l1 |   .3670947   .0603324     6.08   0.000     .2476799    .4865094
           _cons |  -.6732446   .2985506    -2.26   0.026     -1.26416   -.0823292
------------------------------------------------------------------------------

     SE clustered by countryname and year (multiple obs per countryname-year)


. display e(r2_a)
.35522821

. est sto kofalldf

.
. * Summarizing KOF results for political and social globalization
. * S6 Table
. esttab kofpoldf kofsocdf kofalldf using ///
>         kof_polsoc.rtf, replace ///
>         cells(b(star fmt(%9.4f)) se(par)) stats(r2_a N, fmt(2 0)) varwidth(10) ///
>         modelwidth(9) order(kofpogidf_l1 kofsogidf_l1 kofecgidf_l1 po_mean_l1 ///
>         wdi_gdpgr_l1 wdi_gdpcapcur_l1 lr_eco_broad_l1 _cons) ///
>         varlabels(kofpogidf_l1 "political glob." kofsogidf_l1 "social glob." ///
>         kofecgidf_l1 "economic glob." po_mean_l1 "median voter" wdi_gdpgr_l1 "GDP gr
> owth" ///
>         wdi_gdpcapcur_l1 "GDP/capita" lr_eco_broad_l1 "lagged DV" _cons "constant")
> ///
>         mlabels("political" "social" "all three dim.") ///
>         addnote("Standard errors in parentheses; two-sided tests; p < .05 *; p < .01
>  **; p < .001 ***
> ") ///
>         nonumbers nolines
(note: file kof_polsoc.rtf not found)
(output written to kof_polsoc.rtf)
```

```
.
.
. * Descriptive statistics for KOF indices
. * S7 Table
. tabstat kofecgidf_l1 koftrgidf_l1 koffigidf_l1 kofsogidf_l1 kofpogidf_l1 ///
>         kofecgidj_l1 koftrgidj_l1 koffigidj_l1 kofsogidj_l1 kofpogidj_l1, ///
>         statistics(mean sd min max count)

   stats |  kofecg..  koftrg..  koffig..  kofsog..  kofpog..  kofecg..  koftrg..  koff
> ig..  kofsog..
---------+----------------------------------------------------------------------------
> --------------
    mean |  64.02204  56.35476  71.68931  74.41664  85.83986  81.78366  85.08652  78.4
> 0978  75.48084
      sd |  19.51011  19.82085  21.46024  8.298691  12.33718  6.675667  8.176097  7.71
> 4445   9.01913
     min |  22.27495  22.12005  18.08207  50.34094  42.99296  63.52206  60.37791   63.
> 2379  54.47153
     max |  92.73499  89.20579  99.31701  89.28687  99.36353   95.7748  98.65701  96.0
> 5577  91.64677
       N |       129       129       129       129       129       129       129      
>  129       129
--------------------------------------------------------------------------------------
> --------------

   stats |  kofpog..
---------+----------
    mean |  88.71827
      sd |  11.42638
     min |  55.92981
     max |  99.70254
       N |       129
--------------------

.
.
. *** Lowess plots for visual assessment of de jure developments
. * de jure economic globalization
. * Fig 4
. lowess kofecgidj_l1 year, lineopts(color(gs0) lw(medthick)) mc(gs8) ///
>         note("") graphregion(color(white) lwidth(medium)) bwidth(.5) ///
>         legend(off) title("", size(medlarge)) ///
>         xlabel(1975(5)2017) xtitle("") ylabel (60(10)100) ytitle("")

. graph save econdejure, replace
(note: file econdejure.gph not found)
(file econdejure.gph saved)

. graph export fig4.eps, replace
(note: file fig4.eps not found)
(file fig4.eps written in EPS format)

.
. * de jure trade globalization
. lowess koftrgidj_l1 year, lineopts(color(gs0) lw(medthick)) mc(gs8) ///
>         note("") graphregion(color(white) lwidth(medium)) bwidth(.5) ///
>         legend(off) title("{bf:KOF Trade Globalization Index de jure}", size(medlarg
> e)) ///
>         xlabel(1975(10)2017, labsize(medlarge)) xtitle("") ylabel (50(10)100, labsiz
> e(medlarge)) ytit
> le("")
```

```
. graph save tradedejure, replace
(note: file tradedejure.gph not found)
(file tradedejure.gph saved)

.
. * de jure financial globalization
. lowess koffigidj_l1 year, lineopts(color(gs0) lw(medthick)) mc(gs8) ///
>         note("") graphregion(color(white) lwidth(medium)) bwidth(.5) ///
>         legend(off) title("{bf:KOF Financial Globalization Index de jure}", size(med
> large)) ///
>         xlabel(1975(10)2017, labsize(medlarge)) xtitle("") ylabel (50(10)100, labsiz
> e(medlarge)) ytit
> le("")

. graph save financedejure, replace
(note: file financedejure.gph not found)
(file financedejure.gph saved)

.
. * combination of trade and financial de jure
. * S8 Fig
. graph combine tradedejure.gph ///
>         financedejure.gph, ///
>         altshrink graphregion(color(white) lwidth(medium))

. graph save trade_fin_lowess, replace
(note: file trade_fin_lowess.gph not found)
(file trade_fin_lowess.gph saved)

. graph export s8_fig.eps, replace
(note: file s8_fig.eps not found)
(file s8_fig.eps written in EPS format)

.
. * de jure political globalization
. lowess kofpogidj_l1 year, lineopts(color(gs0) lw(medthick)) mc(gs8) ///
>         note("") graphregion(color(white) lwidth(medium)) bwidth(.5) ///
>         legend(off) title("{bf:KOF Political Globalization Index de jure}", size(med
> large)) ///
>         xlabel(1975(10)2015, labsize(medlarge)) xtitle("") ylabel (50(10)100, labsiz
> e(medlarge)) ytit
> le("")

. graph save politicaldejure, replace
(note: file politicaldejure.gph not found)
(file politicaldejure.gph saved)

. graph export politicaldejure.eps, replace
(note: file politicaldejure.eps not found)
(file politicaldejure.eps written in EPS format)

.
. * de jure social globalization
. lowess kofsogidj_l1 year, lineopts(color(gs0) lw(medthick)) mc(gs8) ///
>         note("") graphregion(color(white) lwidth(medium)) bwidth(.5) ///
>         legend(off) title("{bf:KOF Social Globalization Index de jure}", size(medlar
> ge)) ///
>         xlabel(1975(10)2015, labsize(medlarge)) xtitle("") ylabel (50(10)100, labsiz
> e(medlarge)) ytit
> le("")
```

```
. graph save socialdejure, replace
(note: file socialdejure.gph not found)
(file socialdejure.gph saved)

. graph export socialdejure.eps, replace
(note: file socialdejure.eps not found)
(file socialdejure.eps written in EPS format)

.
. * combination of social and political globalization de jure
. * Fig 5
. graph combine socialdejure.gph ///
>         politicaldejure.gph, ///
>         altshrink graphregion(color(white) lwidth(medium))

. graph save social_political_lowess, replace
(note: file social_political_lowess.gph not found)
(file social_political_lowess.gph saved)

. graph export fig5.eps, replace
(note: file fig5.eps not found)
(file fig5.eps written in EPS format)

.
.
. *** Manual moving-window analysis with full model
. gen window_1 = 1 if year < 1994
(76 missing values generated)

. forvalues n = 1/22 {
  2.         local window = 1+`n'
  3.         local start = 1974+`n'
  4.         local stop = 1993+`n'
  5.         gen window_`window' = `window' if year >= `start' & year <= `stop'
  6. }
(71 missing values generated)
(68 missing values generated)
(67 missing values generated)
(69 missing values generated)
(66 missing values generated)
(65 missing values generated)
(64 missing values generated)
(66 missing values generated)
(63 missing values generated)
(63 missing values generated)
(62 missing values generated)
(64 missing values generated)
(61 missing values generated)
(63 missing values generated)
(61 missing values generated)
(63 missing values generated)
(62 missing values generated)
(58 missing values generated)
(58 missing values generated)
(57 missing values generated)
(62 missing values generated)
(61 missing values generated)

.
. foreach v of varlist wdi_export_l1 wdi_import_l1 {
  2.         gen me_lrecomean_`v'_full = .
  3.         label var me_lrecomean_`v'_full "Moving window est for `v'"
  4.         gen upp_lrecomean_`v'_full = .
  5.         label var upp_lrecomean_`v'_full "Moving window est for `v'"
  6.         gen low_lrecomean_`v'_full = .
  7.         label var low_lrecomean_`v'_full "Moving window est for `v'"
  8. }
(129 missing values generated)
(129 missing values generated)
(129 missing values generated)
(129 missing values generated)
(129 missing values generated)
```

```
(129 missing values generated)

.
. forvalues n = 1/23 {
  2.         xtset panelvar_id timevar_id
  3.         quietly: cluster2 lr_eco_broad wdi_export_l1 wdi_import_l1 po_mean_l1 ///
>                 wdi_gdpgr_l1 wdi_gdpcapcur_l1 lr_eco_broad_l1 if window_`n' != ., //
> /
>                 fcluster(countryname) tcluster(year)
  4.         foreach v of varlist wdi_export_l1 wdi_import_l1 {
  5.                 replace me_lrecomean_`v'_full = _b[`v'] in `n'
  6.                 replace me_lrecomean_`v'_full = round(me_lrecomean_`v', .0001)
  7.                 replace upp_lrecomean_`v'_full = _b[`v']+1.96*_se[`v'] in `n'
  8.                 replace upp_lrecomean_`v'_full = round(upp_lrecomean_`v', .0001)
  9.                 replace low_lrecomean_`v'_full = _b[`v']-1.96*_se[`v'] in `n'
 10.                 replace low_lrecomean_`v'_full = round(low_lrecomean_`v', .0001)
 11.         }
 12. }
       panel variable:  panelvar_id (unbalanced)
        time variable:  timevar_id, 1 to 15
                delta:  1 unit
(1 real change made)
(1 real change made)
(1 real change made)
(1 real change made)
(1 real change made)
(1 real change made)
(1 real change made)
(1 real change made)
(1 real change made)
(1 real change made)
(1 real change made)
(1 real change made)
       panel variable:  panelvar_id (unbalanced)
        time variable:  timevar_id, 1 to 15
                delta:  1 unit
(1 real change made)
(1 real change made)
(1 real change made)
(1 real change made)
(1 real change made)
(1 real change made)
(1 real change made)
(1 real change made)
(1 real change made)
(1 real change made)
(1 real change made)
(1 real change made)
       panel variable:  panelvar_id (unbalanced)
        time variable:  timevar_id, 1 to 15
                delta:  1 unit
(1 real change made)
(1 real change made)
(1 real change made)
(1 real change made)
(1 real change made)
(1 real change made)
(1 real change made)
(1 real change made)
(1 real change made)
(1 real change made)
(1 real change made)
(1 real change made)
       panel variable:  panelvar_id (unbalanced)
        time variable:  timevar_id, 1 to 15
                delta:  1 unit
(1 real change made)
(1 real change made)
(1 real change made)
(1 real change made)
(1 real change made)
(1 real change made)
```

```
(1 real change made)
(1 real change made)
(1 real change made)
(1 real change made)
(1 real change made)
(1 real change made)
       panel variable:  panelvar_id (unbalanced)
        time variable:  timevar_id, 1 to 15
                delta:  1 unit
(1 real change made)
(1 real change made)
(1 real change made)
(1 real change made)
(1 real change made)
(1 real change made)
(1 real change made)
(1 real change made)
(1 real change made)
(1 real change made)
(1 real change made)
(1 real change made)
       panel variable:  panelvar_id (unbalanced)
        time variable:  timevar_id, 1 to 15
                delta:  1 unit
(1 real change made)
(1 real change made)
(1 real change made)
(1 real change made)
(1 real change made)
(1 real change made)
(1 real change made)
(1 real change made)
(1 real change made)
(1 real change made)
(1 real change made)
(1 real change made)
       panel variable:  panelvar_id (unbalanced)
        time variable:  timevar_id, 1 to 15
                delta:  1 unit
(1 real change made)
(1 real change made)
(1 real change made)
(1 real change made)
(1 real change made)
(1 real change made)
(1 real change made)
(1 real change made)
(1 real change made)
(1 real change made)
(1 real change made)
(1 real change made)
       panel variable:  panelvar_id (unbalanced)
        time variable:  timevar_id, 1 to 15
                delta:  1 unit
(1 real change made)
(1 real change made)
(1 real change made)
(1 real change made)
(1 real change made)
(1 real change made)
(1 real change made)
(1 real change made)
(1 real change made)
(1 real change made)
(1 real change made)
(1 real change made)
       panel variable:  panelvar_id (unbalanced)
        time variable:  timevar_id, 1 to 15
                delta:  1 unit
(1 real change made)
(1 real change made)
(1 real change made)
```

```
(1 real change made)
(1 real change made)
(1 real change made)
(1 real change made)
(1 real change made)
(1 real change made)
(1 real change made)
(1 real change made)
(1 real change made)
       panel variable:  panelvar_id (unbalanced)
        time variable:  timevar_id, 1 to 15
                delta:  1 unit
(1 real change made)
(1 real change made)
(1 real change made)
(1 real change made)
(1 real change made)
(1 real change made)
(1 real change made)
(1 real change made)
(1 real change made)
(1 real change made)
(1 real change made)
(1 real change made)
       panel variable:  panelvar_id (unbalanced)
        time variable:  timevar_id, 1 to 15
                delta:  1 unit
(1 real change made)
(1 real change made)
(1 real change made)
(1 real change made)
(1 real change made)
(1 real change made)
(1 real change made)
(1 real change made)
(1 real change made)
(1 real change made)
(1 real change made)
(1 real change made)
       panel variable:  panelvar_id (unbalanced)
        time variable:  timevar_id, 1 to 15
                delta:  1 unit
(1 real change made)
(1 real change made)
(1 real change made)
(1 real change made)
(1 real change made)
(1 real change made)
(1 real change made)
(1 real change made)
(1 real change made)
(1 real change made)
(1 real change made)
(1 real change made)
       panel variable:  panelvar_id (unbalanced)
        time variable:  timevar_id, 1 to 15
                delta:  1 unit
(1 real change made)
(1 real change made)
(1 real change made)
(1 real change made)
(1 real change made)
(1 real change made)
(1 real change made)
(1 real change made)
(1 real change made)
(1 real change made)
(1 real change made)
(1 real change made)
       panel variable:  panelvar_id (unbalanced)
        time variable:  timevar_id, 1 to 15
                delta:  1 unit
```

```
(1 real change made)
(1 real change made)
(1 real change made)
(1 real change made)
(1 real change made)
(1 real change made)
(1 real change made)
(1 real change made)
(1 real change made)
(1 real change made)
(1 real change made)
(1 real change made)
       panel variable:  panelvar_id (unbalanced)
        time variable:  timevar_id, 1 to 15
                delta:  1 unit
(1 real change made)
(1 real change made)
(1 real change made)
(1 real change made)
(1 real change made)
(1 real change made)
(1 real change made)
(1 real change made)
(1 real change made)
(1 real change made)
(1 real change made)
(1 real change made)
       panel variable:  panelvar_id (unbalanced)
        time variable:  timevar_id, 1 to 15
                delta:  1 unit
(1 real change made)
(1 real change made)
(1 real change made)
(1 real change made)
(1 real change made)
(1 real change made)
(1 real change made)
(1 real change made)
(1 real change made)
(1 real change made)
(1 real change made)
(1 real change made)
       panel variable:  panelvar_id (unbalanced)
        time variable:  timevar_id, 1 to 15
                delta:  1 unit
(1 real change made)
(1 real change made)
(1 real change made)
(1 real change made)
(1 real change made)
(1 real change made)
(1 real change made)
(1 real change made)
(1 real change made)
(1 real change made)
(1 real change made)
(1 real change made)
       panel variable:  panelvar_id (unbalanced)
        time variable:  timevar_id, 1 to 15
                delta:  1 unit
(1 real change made)
(1 real change made)
(1 real change made)
(1 real change made)
(1 real change made)
(1 real change made)
(1 real change made)
(1 real change made)
(1 real change made)
(1 real change made)
(1 real change made)
(1 real change made)
```

```
       panel variable:  panelvar_id (unbalanced)
        time variable:  timevar_id, 1 to 15
                delta:  1 unit
(1 real change made)
(1 real change made)
(1 real change made)
(1 real change made)
(1 real change made)
(1 real change made)
(1 real change made)
(1 real change made)
(1 real change made)
(1 real change made)
(1 real change made)
(1 real change made)
       panel variable:  panelvar_id (unbalanced)
        time variable:  timevar_id, 1 to 15
                delta:  1 unit
(1 real change made)
(1 real change made)
(1 real change made)
(1 real change made)
(1 real change made)
(1 real change made)
(1 real change made)
(1 real change made)
(1 real change made)
(1 real change made)
(1 real change made)
(1 real change made)
       panel variable:  panelvar_id (unbalanced)
        time variable:  timevar_id, 1 to 15
                delta:  1 unit
(1 real change made)
(1 real change made)
(1 real change made)
(1 real change made)
(1 real change made)
(1 real change made)
(1 real change made)
(1 real change made)
(1 real change made)
(1 real change made)
(1 real change made)
(1 real change made)
       panel variable:  panelvar_id (unbalanced)
        time variable:  timevar_id, 1 to 15
                delta:  1 unit
(1 real change made)
(1 real change made)
(1 real change made)
(1 real change made)
(1 real change made)
(1 real change made)
(1 real change made)
(1 real change made)
(1 real change made)
(1 real change made)
(1 real change made)
(1 real change made)
       panel variable:  panelvar_id (unbalanced)
        time variable:  timevar_id, 1 to 15
                delta:  1 unit
(1 real change made)
(1 real change made)
(1 real change made)
(1 real change made)
(1 real change made)
(1 real change made)
(1 real change made)
(1 real change made)
(1 real change made)
```

```
(1 real change made)
(1 real change made)
(1 real change made)

.
. quietly: reg lr_eco_broad wdi_export_l1 wdi_import_l1 ///
>         po_mean_l1 wdi_gdpgr_l1 wdi_gdpcapcur_l1 lr_eco_broad_l1

. /* quiet regression for generating e(sample) */
. gen window_obs = .
(129 missing values generated)

. label var window_obs "Obs per window"

. gen window_countries = .
(129 missing values generated)

. label var window_countries "Countries per window"

. forvalues n = 1/23 {
  2.         tab country if e(sample) & window_`n' == `n'
  3.         replace window_obs = r(N) in `n'
  4.         replace window_countries = r(r) in `n'
  5. }

         English country name |      Freq.     Percent        Cum.
------------------------------+-----------------------------------
                      Belgium |          6       11.32       11.32
                      Denmark |          7       13.21       24.53
                      Germany |          5        9.43       33.96
                       Greece |          6       11.32       45.28
                      Ireland |          7       13.21       58.49
                        Italy |          5        9.43       67.92
                   Luxembourg |          3        5.66       73.58
                  Netherlands |          5        9.43       83.02
                     Portugal |          2        3.77       86.79
                        Spain |          3        5.66       92.45
               United Kingdom |          4        7.55      100.00
------------------------------+-----------------------------------
                        Total |         53      100.00
(1 real change made)
(1 real change made)

         English country name |      Freq.     Percent        Cum.
------------------------------+-----------------------------------
                      Belgium |          6       10.34       10.34
                      Denmark |          8       13.79       24.14
                      Germany |          6       10.34       34.48
                       Greece |          6       10.34       44.83
                      Ireland |          7       12.07       56.90
                        Italy |          6       10.34       67.24
                   Luxembourg |          4        6.90       74.14
                  Netherlands |          6       10.34       84.48
                     Portugal |          2        3.45       87.93
                        Spain |          3        5.17       93.10
               United Kingdom |          4        6.90      100.00
------------------------------+-----------------------------------
                        Total |         58      100.00
(1 real change made)
(1 real change made)
```

English country name	Freq.	Percent	Cum.
Belgium	7	11.48	11.48
Denmark	8	13.11	24.59
Finland	1	1.64	26.23
Germany	6	9.84	36.07
Greece	6	9.84	45.90
Ireland	7	11.48	57.38
Italy	6	9.84	67.21
Luxembourg	4	6.56	73.77
Netherlands	6	9.84	83.61
Portugal	3	4.92	88.52
Spain	3	4.92	93.44
United Kingdom	4	6.56	100.00
Total	61	100.00	

(1 real change made)
(1 real change made)

English country name	Freq.	Percent	Cum.
Belgium	7	11.29	11.29
Denmark	8	12.90	24.19
Finland	1	1.61	25.81
Germany	5	8.06	33.87
Greece	7	11.29	45.16
Ireland	7	11.29	56.45
Italy	6	9.68	66.13
Luxembourg	4	6.45	72.58
Netherlands	6	9.68	82.26
Portugal	3	4.84	87.10
Spain	4	6.45	93.55
United Kingdom	4	6.45	100.00
Total	62	100.00	

(1 real change made)
(1 real change made)

English country name	Freq.	Percent	Cum.
Belgium	6	10.00	10.00
Denmark	7	11.67	21.67
Finland	1	1.67	23.33
Germany	5	8.33	31.67
Greece	7	11.67	43.33
Ireland	7	11.67	55.00
Italy	6	10.00	65.00
Luxembourg	4	6.67	71.67
Netherlands	5	8.33	80.00
Portugal	3	5.00	85.00
Spain	4	6.67	91.67
United Kingdom	5	8.33	100.00
Total	60	100.00	

(1 real change made)
(1 real change made)

English country name	Freq.	Percent	Cum.
Belgium	5	7.94	7.94
Denmark	8	12.70	20.63
Finland	1	1.59	22.22
Germany	6	9.52	31.75
Greece	7	11.11	42.86
Ireland	7	11.11	53.97
Italy	6	9.52	63.49
Luxembourg	4	6.35	69.84
Netherlands	6	9.52	79.37
Portugal	3	4.76	84.13
Spain	4	6.35	90.48
Sweden	1	1.59	92.06
United Kingdom	5	7.94	100.00

	Freq.	Percent	Cum.
Total	63	100.00	

(1 real change made)
(1 real change made)

English country name	Freq.	Percent	Cum.
Austria	1	1.56	1.56
Belgium	6	9.38	10.94
Denmark	7	10.94	21.88
Finland	2	3.13	25.00
Germany	6	9.38	34.38
Greece	7	10.94	45.31
Ireland	7	10.94	56.25
Italy	5	7.81	64.06
Luxembourg	4	6.25	70.31
Netherlands	6	9.38	79.69
Portugal	4	6.25	85.94
Spain	4	6.25	92.19
Sweden	1	1.56	93.75
United Kingdom	4	6.25	100.00
Total	64	100.00	

(1 real change made)
(1 real change made)

English country name	Freq.	Percent	Cum.
Austria	1	1.54	1.54
Belgium	6	9.23	10.77
Denmark	7	10.77	21.54
Finland	2	3.08	24.62
Germany	5	7.69	32.31
Greece	8	12.31	44.62
Ireland	7	10.77	55.38
Italy	5	7.69	63.08
Luxembourg	4	6.15	69.23
Netherlands	6	9.23	78.46
Portugal	4	6.15	84.62
Spain	5	7.69	92.31
Sweden	1	1.54	93.85
United Kingdom	4	6.15	100.00
Total	65	100.00	

(1 real change made)
(1 real change made)

English country name	Freq.	Percent	Cum.
Austria	1	1.59	1.59
Belgium	5	7.94	9.52
Denmark	7	11.11	20.63
Finland	2	3.17	23.81
Germany	5	7.94	31.75
Greece	7	11.11	42.86
Ireland	6	9.52	52.38
Italy	6	9.52	61.90
Luxembourg	4	6.35	68.25
Netherlands	5	7.94	76.19
Portugal	4	6.35	82.54
Spain	5	7.94	90.48
Sweden	1	1.59	92.06
United Kingdom	5	7.94	100.00
Total	63	100.00	

(1 real change made)
(1 real change made)

English country name	Freq.	Percent	Cum.
Austria	2	3.03	3.03
Belgium	5	7.58	10.61
Denmark	7	10.61	21.21
Finland	2	3.03	24.24
Germany	6	9.09	33.33
Greece	7	10.61	43.94
Ireland	5	7.58	51.52
Italy	6	9.09	60.61
Luxembourg	4	6.06	66.67
Netherlands	5	7.58	74.24
Portugal	5	7.58	81.82
Spain	5	7.58	89.39
Sweden	2	3.03	92.42
United Kingdom	5	7.58	100.00
Total	66	100.00	

(1 real change made)
(1 real change made)

English country name	Freq.	Percent	Cum.
Austria	2	3.03	3.03
Belgium	6	9.09	12.12
Denmark	7	10.61	22.73
Finland	3	4.55	27.27
Germany	5	7.58	34.85
Greece	7	10.61	45.45
Ireland	5	7.58	53.03
Italy	5	7.58	60.61
Luxembourg	4	6.06	66.67
Netherlands	6	9.09	75.76
Portugal	5	7.58	83.33
Spain	5	7.58	90.91
Sweden	2	3.03	93.94
United Kingdom	4	6.06	100.00
Total	66	100.00	

(1 real change made)
(1 real change made)

English country name	Freq.	Percent	Cum.
Austria	2	2.99	2.99
Belgium	6	8.96	11.94
Denmark	6	8.96	20.90
Finland	3	4.48	25.37
Germany	5	7.46	32.84
Greece	8	11.94	44.78
Ireland	5	7.46	52.24
Italy	5	7.46	59.70
Luxembourg	4	5.97	65.67
Netherlands	6	8.96	74.63
Portugal	5	7.46	82.09
Spain	6	8.96	91.04
Sweden	2	2.99	94.03
United Kingdom	4	5.97	100.00
Total	67	100.00	

(1 real change made)
(1 real change made)

English country name	Freq.	Percent	Cum.
Austria	2	3.08	3.08
Belgium	5	7.69	10.77
Denmark	6	9.23	20.00
Finland	3	4.62	24.62
Germany	5	7.69	32.31
Greece	7	10.77	43.08
Ireland	5	7.69	50.77
Italy	5	7.69	58.46
Luxembourg	4	6.15	64.62
Netherlands	6	9.23	73.85
Portugal	5	7.69	81.54
Spain	6	9.23	90.77
Sweden	2	3.08	93.85
United Kingdom	4	6.15	100.00
Total	65	100.00	

(1 real change made)
(1 real change made)

English country name	Freq.	Percent	Cum.
Austria	3	4.41	4.41
Belgium	5	7.35	11.76
Cyprus	1	1.47	13.24
Denmark	6	8.82	22.06
Finland	3	4.41	26.47
Germany	5	7.35	33.82
Greece	7	10.29	44.12
Ireland	5	7.35	51.47
Italy	6	8.82	60.29
Luxembourg	4	5.88	66.18
Netherlands	6	8.82	75.00
Portugal	5	7.35	82.35
Spain	5	7.35	89.71
Sweden	3	4.41	94.12
United Kingdom	4	5.88	100.00
Total	68	100.00	

(1 real change made)
(1 real change made)

English country name	Freq.	Percent	Cum.
Austria	3	4.55	4.55
Belgium	5	7.58	12.12
Cyprus	1	1.52	13.64
Denmark	6	9.09	22.73
Finland	4	6.06	28.79
Germany	4	6.06	34.85
Greece	8	12.12	46.97
Ireland	5	7.58	54.55
Italy	5	7.58	62.12
Luxembourg	4	6.06	68.18
Netherlands	6	9.09	77.27
Portugal	4	6.06	83.33
Spain	5	7.58	90.91
Sweden	3	4.55	95.45
United Kingdom	3	4.55	100.00
Total	66	100.00	

(1 real change made)
(1 real change made)

English country name	Freq.	Percent	Cum.
Austria	4	5.88	5.88
Belgium	5	7.35	13.24
Cyprus	1	1.47	14.71
Denmark	5	7.35	22.06
Finland	4	5.88	27.94
Germany	4	5.88	33.82
Greece	8	11.76	45.59
Ireland	5	7.35	52.94
Italy	6	8.82	61.76
Luxembourg	4	5.88	67.65
Netherlands	6	8.82	76.47
Portugal	4	5.88	82.35
Spain	6	8.82	91.18
Sweden	3	4.41	95.59
United Kingdom	3	4.41	100.00
Total	68	100.00	

(1 real change made)
(1 real change made)

English country name	Freq.	Percent	Cum.
Austria	4	6.06	6.06
Belgium	5	7.58	13.64
Cyprus	1	1.52	15.15
Denmark	5	7.58	22.73
Finland	4	6.06	28.79
Germany	5	7.58	36.36
Greece	7	10.61	46.97
Ireland	4	6.06	53.03
Italy	6	9.09	62.12
Luxembourg	4	6.06	68.18
Netherlands	5	7.58	75.76
Portugal	5	7.58	83.33
Spain	5	7.58	90.91
Sweden	3	4.55	95.45
United Kingdom	3	4.55	100.00
Total	66	100.00	

(1 real change made)
(1 real change made)

English country name	Freq.	Percent	Cum.
Austria	4	5.97	5.97
Belgium	6	8.96	14.93
Cyprus	1	1.49	16.42
Denmark	4	5.97	22.39
Finland	4	5.97	28.36
Germany	4	5.97	34.33
Greece	6	8.96	43.28
Ireland	4	5.97	49.25
Italy	6	8.96	58.21
Luxembourg	4	5.97	64.18
Netherlands	6	8.96	73.13
Portugal	5	7.46	80.60
Spain	5	7.46	88.06
Sweden	4	5.97	94.03
United Kingdom	4	5.97	100.00
Total	67	100.00	

(1 real change made)
(1 real change made)

English country name	Freq.	Percent	Cum.
Austria	4	5.63	5.63
Belgium	5	7.04	12.68
Cyprus	2	2.82	15.49
Denmark	5	7.04	22.54
Finland	5	7.04	29.58
Germany	4	5.63	35.21
Greece	6	8.45	43.66
Ireland	5	7.04	50.70
Italy	6	8.45	59.15
Luxembourg	4	5.63	64.79
Netherlands	6	8.45	73.24
Portugal	5	7.04	80.28
Spain	6	8.45	88.73
Sweden	4	5.63	94.37
United Kingdom	4	5.63	100.00
Total	71	100.00	

(1 real change made)
(1 real change made)

English country name	Freq.	Percent	Cum.
Austria	4	5.63	5.63
Belgium	5	7.04	12.68
Cyprus	2	2.82	15.49
Denmark	5	7.04	22.54
Finland	5	7.04	29.58
Germany	4	5.63	35.21
Greece	8	11.27	46.48
Ireland	4	5.63	52.11
Italy	5	7.04	59.15
Luxembourg	4	5.63	64.79
Netherlands	7	9.86	74.65
Portugal	5	7.04	81.69
Spain	6	8.45	90.14
Sweden	4	5.63	95.77
United Kingdom	3	4.23	100.00
Total	71	100.00	

(1 real change made)
(1 real change made)

English country name	Freq.	Percent	Cum.
Austria	4	5.56	5.56
Belgium	5	6.94	12.50
Cyprus	2	2.78	15.28
Denmark	5	6.94	22.22
Finland	5	6.94	29.17
Germany	5	6.94	36.11
Greece	7	9.72	45.83
Ireland	4	5.56	51.39
Italy	6	8.33	59.72
Luxembourg	5	6.94	66.67
Netherlands	7	9.72	76.39
Portugal	5	6.94	83.33
Spain	5	6.94	90.28
Sweden	4	5.56	95.83
United Kingdom	3	4.17	100.00
Total	72	100.00	

(1 real change made)
(1 real change made)

```
         English country name |      Freq.     Percent        Cum.
------------------------------+-----------------------------------
                      Austria |          4        5.97        5.97
                      Belgium |          5        7.46       13.43
                       Cyprus |          2        2.99       16.42
                      Denmark |          4        5.97       22.39
                      Finland |          5        7.46       29.85
                      Germany |          4        5.97       35.82
                       Greece |          7       10.45       46.27
                      Ireland |          4        5.97       52.24
                        Italy |          5        7.46       59.70
                   Luxembourg |          4        5.97       65.67
                  Netherlands |          6        8.96       74.63
                     Portugal |          5        7.46       82.09
                        Spain |          5        7.46       89.55
                       Sweden |          4        5.97       95.52
               United Kingdom |          3        4.48      100.00
------------------------------+-----------------------------------
                        Total |         67      100.00
(1 real change made)
(1 real change made)

         English country name |      Freq.     Percent        Cum.
------------------------------+-----------------------------------
                      Austria |          4        5.88        5.88
                      Belgium |          4        5.88       11.76
                       Cyprus |          2        2.94       14.71
                      Denmark |          4        5.88       20.59
                      Finland |          4        5.88       26.47
                      Germany |          4        5.88       32.35
                       Greece |          9       13.24       45.59
                      Ireland |          4        5.88       51.47
                        Italy |          5        7.35       58.82
                   Luxembourg |          4        5.88       64.71
                  Netherlands |          6        8.82       73.53
                     Portugal |          4        5.88       79.41
                        Spain |          6        8.82       88.24
                       Sweden |          4        5.88       94.12
               United Kingdom |          4        5.88      100.00
------------------------------+-----------------------------------
                        Total |         68      100.00
(1 real change made)
(1 real change made)

.
. gen window = _n in 1/23
(106 missing values generated)

. label var window "Window variable for histogram"

.
. gen x_var = _n in 1/23
(106 missing values generated)

. label var x_var "Help var for moving window plot"

.
. foreach v of varlist wdi_export_l1 wdi_import_l1 {
  2.         gen green_`v' = 1 if low_lrecomean_`v' > 0
  3.         label var green_`v' "Marginal effect for `v' significant at .05, two-side
> d"
  4.         replace green_`v' = 1 if upp_lrecomean_`v' < 0
  5.         replace green_`v' = 0 if green_`v' == .
  6. }
(6 missing values generated)
(0 real changes made)
(6 real changes made)
(23 missing values generated)
(17 real changes made)
(6 real changes made)
```

```
.
. * Plot for imports
. scatter x_var upp_lrecomean_wdi_import_l1 if green_wdi_import_l1 == 1, ///
>         ms(i) mlabpos(3) mlabel(window_obs) mlabcolor(gs0) mlabsize(medlarge) || ///
> scatter x_var upp_lrecomean_wdi_import_l1 if green_wdi_import_l1 == 0, ///
>         ms(i) mlabpos(3) mlabel(window_obs) mlabcolor(gs10) mlabsize(medlarge) || //
> /
> scatter x_var me_lrecomean_wdi_import_l1 if green_wdi_import_l1 == 1, ///
>         mcolor(gs0) legend(off) || ///
> scatter x_var me_lrecomean_wdi_import_l1 if green_wdi_import_l1 == 0, ///
>         mcolor(gs10) legend(off) || ///
> rspike upp_lrecomean_wdi_import_l1 low_lrecomean_wdi_import_l1 x_var if ///
>         green_wdi_import_l1 == 1, lc(gs0) horizontal || ///
> rspike upp_lrecomean_wdi_import_l1 low_lrecomean_wdi_import_l1 x_var if ///
>         green_wdi_import_l1 == 0, lc(gs10) horizontal ///
>         ytitle("") ylabel(1(5)23) title("{bf:Imports}", size(medlarge)) ///
>         xlabel(, labsize(medlarge)) xtitle("") ///
>         ylabel(1 "1974-1993" 2 "1975-1994" 3 "1976-1995" 4 "1977-1996" 5 "1978-1997"
>  ///
>         6 "1979-1998" 7 "1980-1999" 8 "1981-2000" 9 "1982-2001" 10 "1983-2002" ///
>         11 "1984-2003" 12 "1985-2004" 13 "1986-2005" 14 "1987-2006" 15 "1988-2007" /
> //
>         16 "1989-2008" 17 "1990-2009" 18 "1991-2010" 19 "1992-2011" 20 "1993-2012" /
> //
>         21 "1994-2013" 22 "1995-2014" 23 "1996-2015", ///
>         labsize(medlarge) angle(0) nogrid) ///
>         xline(0, lp(dash) lc(gs8) lwidth(thin)) ///
>         legend(off) graphregion(color(white) lwidth(medium)) // note("Numbers next t
> o confidence inte
> rvals are observations per window", span)

. graph save center_window_imports_vertical_95.gph, replace
(note: file center_window_imports_vertical_95.gph not found)
(file center_window_imports_vertical_95.gph saved)

.
. * Plot for exports
. scatter x_var upp_lrecomean_wdi_export_l1 if green_wdi_export_l1 == 1, ///
>         ms(i) mlabpos(3) mlabel(window_obs) mlabcolor(gs0) mlabsize(medlarge) || ///
> scatter x_var upp_lrecomean_wdi_export_l1 if green_wdi_export_l1 == 0, ///
>         ms(i) mlabpos(3) mlabel(window_obs) mlabcolor(gs10) mlabsize(medlarge) || //
> /
> scatter x_var me_lrecomean_wdi_export_l1 if green_wdi_export_l1 == 1, ///
>         mcolor(gs0) legend(off) || ///
> scatter x_var me_lrecomean_wdi_export_l1 if green_wdi_export_l1 == 0, ///
>         mcolor(gs10) legend(off) || ///
> rspike upp_lrecomean_wdi_export_l1 low_lrecomean_wdi_export_l1 x_var if ///
>         green_wdi_export_l1 == 1, lc(gs0) horizontal || ///
> rspike upp_lrecomean_wdi_export_l1 low_lrecomean_wdi_export_l1 x_var if ///
> green_wdi_export_l1 == 0, lc(gs10) horizontal ///
>         ytitle("") ylabel(1(5)23) title("{bf:Exports}", size(medlarge)) ///
>         xlabel(, labsize(medlarge)) xtitle("") ///
>         ylabel(1 "1974-1993" 2 "1975-1994" 3 "1976-1995" 4 "1977-1996" 5 "1978-1997"
>  ///
>         6 "1979-1998" 7 "1980-1999" 8 "1981-2000" 9 "1982-2001" 10 "1983-2002" ///
>         11 "1984-2003" 12 "1985-2004" 13 "1986-2005" 14 "1987-2006" 15 "1988-2007" /
> //
>         16 "1989-2008" 17 "1990-2009" 18 "1991-2010" 19 "1992-2011" 20 "1993-2012" /
> //
>         21 "1994-2013" 22 "1995-2014" 23 "1996-2015", ///
>         labsize(medlarge) angle(0) nogrid) ///
>         xline(0, lp(dash) lc(gs8) lwidth(thin)) ///
>         legend(off) graphregion(color(white) lwidth(medium)) // note("Numbers next t
> o confidence inte
> rvals are observations per window", span)
```

```
. graph save center_window_exports_vertical_95.gph, replace
(note: file center_window_exports_vertical_95.gph not found)
(file center_window_exports_vertical_95.gph saved)

.
. * Fig 3
. graph combine center_window_imports_vertical_95.gph ///
>         center_window_exports_vertical_95.gph ///
>         , row(1) ///
>         altshrink graphregion(color(white) lwidth(medium))

. graph save center_window_comb, replace
(note: file center_window_comb.gph not found)
(file center_window_comb.gph saved)

. graph export fig3.eps, replace
(note: file fig3.eps not found)
(file fig3.eps written in EPS format)

.
. drop window_* green_* window

.
. * Country-wise deletion, preparatory work
. foreach v of varlist wdi_export_l1 wdi_import_l1 {
  2.         gen me_cwise_`v'_full = .
  3.         label var me_cwise_`v'_full "Marg eff for country-wise deletion"
  4.         gen upp_cwise_`v'_full = .
  5.         label var upp_cwise_`v'_full "Upper CI for country-wise deletion"
  6.         gen low_cwise_`v'_full = .
  7.         label var low_cwise_`v'_full "Lower CI for country-wise deletion"
  8. }
(129 missing values generated)
(129 missing values generated)
(129 missing values generated)
(129 missing values generated)
(129 missing values generated)
(129 missing values generated)

.
. quietly: tab country, gen(c)

.
. * Country-wise deletion with 95% CI
. forvalues n = 1/15 {
  2.         quietly: cluster2 lr_eco_broad wdi_export_l1 wdi_import_l1 po_mean_l1 ///
>                 wdi_gdpgr_l1 wdi_gdpcapcur_l1 lr_eco_broad_l1 if c`n' == 0, ///
>                 fcluster(countryname) tcluster(year)
  3.         foreach v of varlist wdi_export_l1 wdi_import_l1 {
  4.                 replace me_cwise_`v'_full = _b[`v'] in `n'
  5.                 replace upp_cwise_`v'_full = _b[`v']+1.96*_se[`v'] in `n'
  6.                 replace low_cwise_`v'_full = _b[`v']-1.96*_se[`v'] in `n'
  7.         }
  8. }
(1 real change made)
(1 real change made)
(1 real change made)
(1 real change made)
(1 real change made)
(1 real change made)
(1 real change made)
(1 real change made)
(1 real change made)
(1 real change made)
(1 real change made)
(1 real change made)
(1 real change made)
(1 real change made)
(1 real change made)
(1 real change made)
(1 real change made)
(1 real change made)
```

```
(1 real change made)
(1 real change made)
(1 real change made)
(1 real change made)
(1 real change made)
(1 real change made)
(1 real change made)
(1 real change made)
(1 real change made)
(1 real change made)
(1 real change made)
(1 real change made)
(1 real change made)
(1 real change made)
(1 real change made)
(1 real change made)
(1 real change made)
(1 real change made)
(1 real change made)
(1 real change made)
(1 real change made)
(1 real change made)
(1 real change made)
(1 real change made)
(1 real change made)
(1 real change made)
(1 real change made)
(1 real change made)
(1 real change made)
(1 real change made)
(1 real change made)
(1 real change made)
(1 real change made)
(1 real change made)
(1 real change made)
(1 real change made)
(1 real change made)
(1 real change made)
(1 real change made)
(1 real change made)
(1 real change made)
(1 real change made)
(1 real change made)
(1 real change made)
(1 real change made)
(1 real change made)
(1 real change made)
(1 real change made)
(1 real change made)
(1 real change made)
(1 real change made)
(1 real change made)
(1 real change made)
(1 real change made)
(1 real change made)
(1 real change made)
(1 real change made)
(1 real change made)
(1 real change made)
(1 real change made)
(1 real change made)
(1 real change made)
(1 real change made)
(1 real change made)
(1 real change made)
(1 real change made)
(1 real change made)
(1 real change made)
(1 real change made)
(1 real change made)
(1 real change made)
(1 real change made)
```

```
.
. * S5 Fig
. scatter me_cwise_wdi_export_l1_full x_var in 1/15, ///
>         mc(gs0) ///
>         xlabel(1(1)15) || ///
> rspike upp_cwise_wdi_export_l1_full low_cwise_wdi_export_l1_full x_var in 1/15, ///
>         lc(gs0) graphregion(color(white) lwidth(medium)) ///
>         xlabel(1(1)15) xtitle(" " "No. of deleted country according to dummy list",
> size(medium)) ///
>         ytitle("Marginal effect and 95% CI for exports" " ", size(medium)) ///
>         yline(0, lp(dash) lc(gs0)) ///
>         legend(off)

. graph save cwise_exports_95_full, replace
(note: file cwise_exports_95_full.gph not found)
(file cwise_exports_95_full.gph saved)

. graph export s5_fig.eps, replace
(note: file s5_fig.eps not found)
(file s5_fig.eps written in EPS format)

.
. * S6 Fig
. scatter me_cwise_wdi_import_l1_full x_var in 1/15, ///
>         mc(gs0) ///
>         xlabel(1(1)15) || ///
> rspike upp_cwise_wdi_import_l1_full low_cwise_wdi_import_l1_full x_var in 1/15, ///
>         lc(gs0) graphregion(color(white) lwidth(medium)) ///
>         xlabel(1(1)15) xtitle(" " "No. of deleted country according to dummy list",
> size(medium)) ///
>         ytitle("Marginal effect and 95% CI for imports" " ", size(medium)) yline(0,
> lp(dash) ///
>         lc(gs0)) legend(off)

. graph save cwise_imports_95_full, replace
(note: file cwise_imports_95_full.gph not found)
(file cwise_imports_95_full.gph saved)

. graph export s6_fig.eps, replace
(note: file s6_fig.eps not found)
(file s6_fig.eps written in EPS format)

.
. capture drop me_lrecomean_wdi_export_l1_full - low_cwise_wdi_import_l1_full

. capture drop me_prais_full - _est_noedu_base

.
. *** Manual moving-window analysis for trade balance with full model
. gen window_1 = 1 if year < 1994
(76 missing values generated)

. forvalues n = 1/22 {
  2.         local window = 1+`n'
  3.         local start = 1974+`n'
  4.         local stop = 1993+`n'
  5.         gen window_`window' = `window' if year >= `start' & year <= `stop'
  6. }
(71 missing values generated)
(68 missing values generated)
(67 missing values generated)
(69 missing values generated)
(66 missing values generated)
(65 missing values generated)
(64 missing values generated)
(66 missing values generated)
(63 missing values generated)
(63 missing values generated)
(62 missing values generated)
(64 missing values generated)
(61 missing values generated)
```

```
(63 missing values generated)
(61 missing values generated)
(63 missing values generated)
(62 missing values generated)
(58 missing values generated)
(58 missing values generated)
(57 missing values generated)
(62 missing values generated)
(61 missing values generated)

.
. foreach v of varlist tradebal_wdi_l1 {
  2.         gen me_lr_`v'_full = .
  3.         label var me_lr_`v'_full "Moving window est for `v'"
  4.         gen upp_lr_`v'_full = .
  5.         label var upp_lr_`v'_full "Moving window est for `v'"
  6.         gen low_lr_`v'_full = .
  7.         label var low_lr_`v'_full "Moving window est for `v'"
  8. }
(129 missing values generated)
(129 missing values generated)
(129 missing values generated)

.
. forvalues n = 1/23 {
  2.         quietly: cluster2 lr_eco_broad tradebal_wdi_l1 po_mean_l1 ///
>                 wdi_gdpgr_l1 wdi_gdpcapcur_l1 lr_eco_broad_l1 if window_`n' != ., //
> /
>                 fcluster(countryname) tcluster(year)
  3.         foreach v of varlist tradebal_wdi_l1 {
  4.                 replace me_lr_`v'_full = _b[`v'] in `n'
  5.                 replace me_lr_`v'_full = round(me_lr_`v', .0001)
  6.                 replace upp_lr_`v'_full = _b[`v']+1.96*_se[`v'] in `n'
  7.                 replace upp_lr_`v'_full = round(upp_lr_`v', .0001)
  8.                 replace low_lr_`v'_full = _b[`v']-1.96*_se[`v'] in `n'
  9.                 replace low_lr_`v'_full = round(low_lr_`v', .0001)
 10.         }
 11. }
(1 real change made)
(1 real change made)
(1 real change made)
(1 real change made)
(1 real change made)
(1 real change made)
(1 real change made)
(1 real change made)
(1 real change made)
(1 real change made)
(1 real change made)
(1 real change made)
(1 real change made)
(1 real change made)
(1 real change made)
(1 real change made)
(1 real change made)
(1 real change made)
(1 real change made)
(1 real change made)
(1 real change made)
(1 real change made)
(1 real change made)
(1 real change made)
(1 real change made)
(1 real change made)
(1 real change made)
(1 real change made)
(1 real change made)
(1 real change made)
(1 real change made)
(1 real change made)
(1 real change made)
(1 real change made)
```

```
(1 real change made)
(1 real change made)
(1 real change made)
(1 real change made)
(1 real change made)
(1 real change made)
(1 real change made)
(1 real change made)
(1 real change made)
(1 real change made)
(1 real change made)
(1 real change made)
(1 real change made)
(1 real change made)
(1 real change made)
(1 real change made)
(1 real change made)
(1 real change made)
(1 real change made)
(1 real change made)
(1 real change made)
(1 real change made)
(1 real change made)
(1 real change made)
(1 real change made)
(1 real change made)
(1 real change made)
(1 real change made)
(1 real change made)
(1 real change made)
(1 real change made)
(1 real change made)
(1 real change made)
(1 real change made)
(1 real change made)
(1 real change made)
(1 real change made)
(1 real change made)
(1 real change made)
(1 real change made)
(1 real change made)
(1 real change made)
(1 real change made)
(1 real change made)
(1 real change made)
(1 real change made)
(1 real change made)
(1 real change made)
(1 real change made)
(1 real change made)
(1 real change made)
(1 real change made)
(1 real change made)
(1 real change made)
(1 real change made)
(1 real change made)
(1 real change made)
(1 real change made)
(1 real change made)
(1 real change made)
(1 real change made)
(1 real change made)
(1 real change made)
(1 real change made)
(1 real change made)
(1 real change made)
(1 real change made)
(1 real change made)
(1 real change made)
(1 real change made)
(1 real change made)
(1 real change made)
```

```
(1 real change made)
(1 real change made)
(1 real change made)
(1 real change made)
(1 real change made)
(1 real change made)
(1 real change made)
(1 real change made)
(1 real change made)
(1 real change made)
(1 real change made)
(1 real change made)
(1 real change made)
(1 real change made)
(1 real change made)
(1 real change made)
(1 real change made)
(1 real change made)
(1 real change made)
(1 real change made)
(1 real change made)
(1 real change made)
(1 real change made)
(1 real change made)
(1 real change made)
(1 real change made)
(1 real change made)
(1 real change made)
(1 real change made)
(1 real change made)
(1 real change made)
(1 real change made)

.
. quietly: reg lr_eco_broad tradebal_wdi_l1 po_mean_l1 ///
>         wdi_gdpgr_l1 wdi_gdpcapcur_l1 lr_eco_broad_l1, cluster(countryname)

. /* quiet regression for generating e(sample) */
. gen window_obs = .
(129 missing values generated)

. label var window_obs "Obs per window"

. gen window_countries = .
(129 missing values generated)

. label var window_countries "Countries per window"

. forvalues n = 1/23 {
  2.         tab country if e(sample) & window_`n' == `n'
  3.         replace window_obs = r(N) in `n'
  4.         replace window_countries = r(r) in `n'
  5. }

         English country name |      Freq.     Percent        Cum.
------------------------------+-----------------------------------
                      Belgium |          6       11.32       11.32
                      Denmark |          7       13.21       24.53
                      Germany |          5        9.43       33.96
                       Greece |          6       11.32       45.28
                      Ireland |          7       13.21       58.49
                        Italy |          5        9.43       67.92
                   Luxembourg |          3        5.66       73.58
                  Netherlands |          5        9.43       83.02
                     Portugal |          2        3.77       86.79
                        Spain |          3        5.66       92.45
               United Kingdom |          4        7.55      100.00
------------------------------+-----------------------------------
                        Total |         53      100.00
(1 real change made)
(1 real change made)
```

English country name	Freq.	Percent	Cum.
Belgium	6	10.34	10.34
Denmark	8	13.79	24.14
Germany	6	10.34	34.48
Greece	6	10.34	44.83
Ireland	7	12.07	56.90
Italy	6	10.34	67.24
Luxembourg	4	6.90	74.14
Netherlands	6	10.34	84.48
Portugal	2	3.45	87.93
Spain	3	5.17	93.10
United Kingdom	4	6.90	100.00
Total	58	100.00	

(1 real change made)
(1 real change made)

English country name	Freq.	Percent	Cum.
Belgium	7	11.48	11.48
Denmark	8	13.11	24.59
Finland	1	1.64	26.23
Germany	6	9.84	36.07
Greece	6	9.84	45.90
Ireland	7	11.48	57.38
Italy	6	9.84	67.21
Luxembourg	4	6.56	73.77
Netherlands	6	9.84	83.61
Portugal	3	4.92	88.52
Spain	3	4.92	93.44
United Kingdom	4	6.56	100.00
Total	61	100.00	

(1 real change made)
(1 real change made)

English country name	Freq.	Percent	Cum.
Belgium	7	11.29	11.29
Denmark	8	12.90	24.19
Finland	1	1.61	25.81
Germany	5	8.06	33.87
Greece	7	11.29	45.16
Ireland	7	11.29	56.45
Italy	6	9.68	66.13
Luxembourg	4	6.45	72.58
Netherlands	6	9.68	82.26
Portugal	3	4.84	87.10
Spain	4	6.45	93.55
United Kingdom	4	6.45	100.00
Total	62	100.00	

(1 real change made)
(1 real change made)

English country name	Freq.	Percent	Cum.
Belgium	6	10.00	10.00
Denmark	7	11.67	21.67
Finland	1	1.67	23.33
Germany	5	8.33	31.67
Greece	7	11.67	43.33
Ireland	7	11.67	55.00
Italy	6	10.00	65.00
Luxembourg	4	6.67	71.67
Netherlands	5	8.33	80.00
Portugal	3	5.00	85.00
Spain	4	6.67	91.67
United Kingdom	5	8.33	100.00
Total	60	100.00	

(1 real change made)
(1 real change made)

English country name	Freq.	Percent	Cum.
Belgium	5	7.94	7.94
Denmark	8	12.70	20.63
Finland	1	1.59	22.22
Germany	6	9.52	31.75
Greece	7	11.11	42.86
Ireland	7	11.11	53.97
Italy	6	9.52	63.49
Luxembourg	4	6.35	69.84
Netherlands	6	9.52	79.37
Portugal	3	4.76	84.13
Spain	4	6.35	90.48
Sweden	1	1.59	92.06
United Kingdom	5	7.94	100.00
Total	63	100.00	

(1 real change made)
(1 real change made)

English country name	Freq.	Percent	Cum.
Austria	1	1.56	1.56
Belgium	6	9.38	10.94
Denmark	7	10.94	21.88
Finland	2	3.13	25.00
Germany	6	9.38	34.38
Greece	7	10.94	45.31
Ireland	7	10.94	56.25
Italy	5	7.81	64.06
Luxembourg	4	6.25	70.31
Netherlands	6	9.38	79.69
Portugal	4	6.25	85.94
Spain	4	6.25	92.19
Sweden	1	1.56	93.75
United Kingdom	4	6.25	100.00
Total	64	100.00	

(1 real change made)
(1 real change made)

English country name	Freq.	Percent	Cum.
Austria	1	1.54	1.54
Belgium	6	9.23	10.77
Denmark	7	10.77	21.54
Finland	2	3.08	24.62
Germany	5	7.69	32.31
Greece	8	12.31	44.62
Ireland	7	10.77	55.38
Italy	5	7.69	63.08
Luxembourg	4	6.15	69.23
Netherlands	6	9.23	78.46
Portugal	4	6.15	84.62
Spain	5	7.69	92.31
Sweden	1	1.54	93.85
United Kingdom	4	6.15	100.00
Total	65	100.00	

(1 real change made)
(1 real change made)

English country name	Freq.	Percent	Cum.
Austria	1	1.59	1.59
Belgium	5	7.94	9.52
Denmark	7	11.11	20.63
Finland	2	3.17	23.81
Germany	5	7.94	31.75
Greece	7	11.11	42.86
Ireland	6	9.52	52.38
Italy	6	9.52	61.90
Luxembourg	4	6.35	68.25
Netherlands	5	7.94	76.19
Portugal	4	6.35	82.54
Spain	5	7.94	90.48
Sweden	1	1.59	92.06
United Kingdom	5	7.94	100.00
Total	63	100.00	

(1 real change made)
(1 real change made)

English country name	Freq.	Percent	Cum.
Austria	2	3.03	3.03
Belgium	5	7.58	10.61
Denmark	7	10.61	21.21
Finland	2	3.03	24.24
Germany	6	9.09	33.33
Greece	7	10.61	43.94
Ireland	5	7.58	51.52
Italy	6	9.09	60.61
Luxembourg	4	6.06	66.67
Netherlands	5	7.58	74.24
Portugal	5	7.58	81.82
Spain	5	7.58	89.39
Sweden	2	3.03	92.42
United Kingdom	5	7.58	100.00
Total	66	100.00	

(1 real change made)
(1 real change made)

English country name	Freq.	Percent	Cum.
Austria	2	3.03	3.03
Belgium	6	9.09	12.12
Denmark	7	10.61	22.73
Finland	3	4.55	27.27
Germany	5	7.58	34.85
Greece	7	10.61	45.45
Ireland	5	7.58	53.03
Italy	5	7.58	60.61
Luxembourg	4	6.06	66.67
Netherlands	6	9.09	75.76
Portugal	5	7.58	83.33
Spain	5	7.58	90.91
Sweden	2	3.03	93.94
United Kingdom	4	6.06	100.00
Total	66	100.00	

(1 real change made)
(1 real change made)

English country name	Freq.	Percent	Cum.
Austria	2	2.99	2.99
Belgium	6	8.96	11.94
Denmark	6	8.96	20.90
Finland	3	4.48	25.37
Germany	5	7.46	32.84
Greece	8	11.94	44.78
Ireland	5	7.46	52.24
Italy	5	7.46	59.70
Luxembourg	4	5.97	65.67
Netherlands	6	8.96	74.63
Portugal	5	7.46	82.09
Spain	6	8.96	91.04
Sweden	2	2.99	94.03
United Kingdom	4	5.97	100.00
Total	67	100.00	

(1 real change made)
(1 real change made)

English country name	Freq.	Percent	Cum.
Austria	2	3.08	3.08
Belgium	5	7.69	10.77
Denmark	6	9.23	20.00
Finland	3	4.62	24.62
Germany	5	7.69	32.31
Greece	7	10.77	43.08
Ireland	5	7.69	50.77
Italy	5	7.69	58.46
Luxembourg	4	6.15	64.62
Netherlands	6	9.23	73.85
Portugal	5	7.69	81.54
Spain	6	9.23	90.77
Sweden	2	3.08	93.85
United Kingdom	4	6.15	100.00
Total	65	100.00	

(1 real change made)
(1 real change made)

English country name	Freq.	Percent	Cum.
Austria	3	4.41	4.41
Belgium	5	7.35	11.76
Cyprus	1	1.47	13.24
Denmark	6	8.82	22.06
Finland	3	4.41	26.47
Germany	5	7.35	33.82
Greece	7	10.29	44.12
Ireland	5	7.35	51.47
Italy	6	8.82	60.29
Luxembourg	4	5.88	66.18
Netherlands	6	8.82	75.00
Portugal	5	7.35	82.35
Spain	5	7.35	89.71
Sweden	3	4.41	94.12
United Kingdom	4	5.88	100.00
Total	68	100.00	

(1 real change made)
(1 real change made)

English country name	Freq.	Percent	Cum.
Austria	3	4.55	4.55
Belgium	5	7.58	12.12
Cyprus	1	1.52	13.64
Denmark	6	9.09	22.73
Finland	4	6.06	28.79
Germany	4	6.06	34.85
Greece	8	12.12	46.97
Ireland	5	7.58	54.55
Italy	5	7.58	62.12
Luxembourg	4	6.06	68.18
Netherlands	6	9.09	77.27
Portugal	4	6.06	83.33
Spain	5	7.58	90.91
Sweden	3	4.55	95.45
United Kingdom	3	4.55	100.00
Total	66	100.00	

(1 real change made)
(1 real change made)

English country name	Freq.	Percent	Cum.
Austria	4	5.88	5.88
Belgium	5	7.35	13.24
Cyprus	1	1.47	14.71
Denmark	5	7.35	22.06
Finland	4	5.88	27.94
Germany	4	5.88	33.82
Greece	8	11.76	45.59
Ireland	5	7.35	52.94
Italy	6	8.82	61.76
Luxembourg	4	5.88	67.65
Netherlands	6	8.82	76.47
Portugal	4	5.88	82.35
Spain	6	8.82	91.18
Sweden	3	4.41	95.59
United Kingdom	3	4.41	100.00
Total	68	100.00	

(1 real change made)
(1 real change made)

English country name	Freq.	Percent	Cum.
Austria	4	6.06	6.06
Belgium	5	7.58	13.64
Cyprus	1	1.52	15.15
Denmark	5	7.58	22.73
Finland	4	6.06	28.79
Germany	5	7.58	36.36
Greece	7	10.61	46.97
Ireland	4	6.06	53.03
Italy	6	9.09	62.12
Luxembourg	4	6.06	68.18
Netherlands	5	7.58	75.76
Portugal	5	7.58	83.33
Spain	5	7.58	90.91
Sweden	3	4.55	95.45
United Kingdom	3	4.55	100.00
Total	66	100.00	

(1 real change made)
(1 real change made)

English country name	Freq.	Percent	Cum.
Austria	4	5.97	5.97
Belgium	6	8.96	14.93
Cyprus	1	1.49	16.42
Denmark	4	5.97	22.39
Finland	4	5.97	28.36
Germany	4	5.97	34.33
Greece	6	8.96	43.28
Ireland	4	5.97	49.25
Italy	6	8.96	58.21
Luxembourg	4	5.97	64.18
Netherlands	6	8.96	73.13
Portugal	5	7.46	80.60
Spain	5	7.46	88.06
Sweden	4	5.97	94.03
United Kingdom	4	5.97	100.00
Total	67	100.00	

(1 real change made)
(1 real change made)

English country name	Freq.	Percent	Cum.
Austria	4	5.63	5.63
Belgium	5	7.04	12.68
Cyprus	2	2.82	15.49
Denmark	5	7.04	22.54
Finland	5	7.04	29.58
Germany	4	5.63	35.21
Greece	6	8.45	43.66
Ireland	5	7.04	50.70
Italy	6	8.45	59.15
Luxembourg	4	5.63	64.79
Netherlands	6	8.45	73.24
Portugal	5	7.04	80.28
Spain	6	8.45	88.73
Sweden	4	5.63	94.37
United Kingdom	4	5.63	100.00
Total	71	100.00	

(1 real change made)
(1 real change made)

English country name	Freq.	Percent	Cum.
Austria	4	5.63	5.63
Belgium	5	7.04	12.68
Cyprus	2	2.82	15.49
Denmark	5	7.04	22.54
Finland	5	7.04	29.58
Germany	4	5.63	35.21
Greece	8	11.27	46.48
Ireland	4	5.63	52.11
Italy	5	7.04	59.15
Luxembourg	4	5.63	64.79
Netherlands	7	9.86	74.65
Portugal	5	7.04	81.69
Spain	6	8.45	90.14
Sweden	4	5.63	95.77
United Kingdom	3	4.23	100.00
Total	71	100.00	

(1 real change made)
(1 real change made)

English country name	Freq.	Percent	Cum.
Austria	4	5.56	5.56
Belgium	5	6.94	12.50
Cyprus	2	2.78	15.28
Denmark	5	6.94	22.22
Finland	5	6.94	29.17
Germany	5	6.94	36.11
Greece	7	9.72	45.83
Ireland	4	5.56	51.39
Italy	6	8.33	59.72
Luxembourg	5	6.94	66.67
Netherlands	7	9.72	76.39
Portugal	5	6.94	83.33
Spain	5	6.94	90.28
Sweden	4	5.56	95.83
United Kingdom	3	4.17	100.00
Total	72	100.00	

(1 real change made)
(1 real change made)

English country name	Freq.	Percent	Cum.
Austria	4	5.97	5.97
Belgium	5	7.46	13.43
Cyprus	2	2.99	16.42
Denmark	4	5.97	22.39
Finland	5	7.46	29.85
Germany	4	5.97	35.82
Greece	7	10.45	46.27
Ireland	4	5.97	52.24
Italy	5	7.46	59.70
Luxembourg	4	5.97	65.67
Netherlands	6	8.96	74.63
Portugal	5	7.46	82.09
Spain	5	7.46	89.55
Sweden	4	5.97	95.52
United Kingdom	3	4.48	100.00
Total	67	100.00	

(1 real change made)
(1 real change made)

English country name	Freq.	Percent	Cum.
Austria	4	5.88	5.88
Belgium	4	5.88	11.76
Cyprus	2	2.94	14.71
Denmark	4	5.88	20.59
Finland	4	5.88	26.47
Germany	4	5.88	32.35
Greece	9	13.24	45.59
Ireland	4	5.88	51.47
Italy	5	7.35	58.82
Luxembourg	4	5.88	64.71
Netherlands	6	8.82	73.53
Portugal	4	5.88	79.41
Spain	6	8.82	88.24
Sweden	4	5.88	94.12
United Kingdom	4	5.88	100.00
Total	68	100.00	

(1 real change made)
(1 real change made)

```
.
. gen x_var = _n in 1/23
(106 missing values generated)

. label var x_var "Help var for moving window plot"

.
. * Visual representation
. foreach v of varlist tradebal_wdi_l1 {
  2.         gen green_`v' = 1 if low_lr_`v' > 0
  3.         label var green_`v' "Marginal effect for `v' significant at .05, two-side
> d"
  4.         replace green_`v' = 1 if upp_lr_`v' < 0
  5.         replace green_`v' = 0 if green_`v' == .
  6. }
(12 missing values generated)
(0 real changes made)
(12 real changes made)

.
. * Plot for trade balance
. * S7 Fig
. scatter x_var upp_lr_tradebal_wdi_l1 if green_tradebal_wdi_l1 == 1, ///
>         ms(i) mlabpos(3) mlabel(window_obs) mlabcolor(gs0) mlabsize(medium) || ///
> scatter x_var upp_lr_tradebal_wdi_l1 if green_tradebal_wdi_l1 == 0, ///
>         ms(i) mlabpos(3) mlabel(window_obs) mlabcolor(gs10) mlabsize(medium) || ///
> scatter x_var upp_lr_tradebal_wdi_l1, ms(i) mlabpos(3) mlabel(window_obs) ///
>         mlabcolor(gs0) mlabsize(medium) || ///
> scatter x_var me_lr_tradebal_wdi_l1 if green_tradebal_wdi_l1 == 1, ///
>         mcolor(gs0) legend(off) || ///
> scatter x_var me_lr_tradebal_wdi_l1 if green_tradebal_wdi_l1 == 0, ///
>         mcolor(gs10) legend(off) || ///
> rspike upp_lr_tradebal_wdi_l1 low_lr_tradebal_wdi_l1 x_var if ///
>         green_tradebal_wdi_l1 == 1, lc(gs0) horizontal || ///
> rspike upp_lr_tradebal_wdi_l1 low_lr_tradebal_wdi_l1 x_var if ///
> green_tradebal_wdi_l1 == 0, lc(gs10) horizontal ///
>         ytitle("") ylabel(1(5)23) title("") ///
>         xtitle("") ///
>         ylabel(1 "1974-1993" 3 "1976-1995" 5 "1978-1997" ///
>         7 "1980-1999" 9 "1982-2001" ///
>         11 "1984-2003" 13 "1986-2005" 15 "1988-2007" ///
>         17 "1990-2009" 19 "1992-2011" ///
>         21 "1994-2013" 23 "1996-2015", ///
>         angle(0) nogrid) ///
>         xline(0, lp(dash) lc(gs8) lwidth(thin)) ///
>         legend(off) graphregion(color(white) lwidth(medium)) // note("Numbers next t
> o confidence inte
> rvals are observations per window", span)

. graph save center_window_tradebal_vertical_95.gph, replace
(note: file center_window_tradebal_vertical_95.gph not found)
(file center_window_tradebal_vertical_95.gph saved)

. graph export s7_fig.eps, replace
(note: file s7_fig.eps not found)
(file s7_fig.eps written in EPS format)

.
. drop window_*
```

```
.
. *** Case-wise deletion
. foreach v of varlist wdi_export_l1 wdi_import_l1 {
  2.         gen me_casew_`v'_full = .
  3.         label var me_casew_`v'_full "Marg eff of `v' if i'th case dropped"
  4.         gen upp_casew_`v'_full_95 = .
  5.         label var upp_casew_`v'_full_95 "Upper 95% CI for `v' if i'th case droppe
> d"
  6.         gen low_casew_`v'_full_95 = .
  7.         label var low_casew_`v'_full_95 "Lower 95% CI for `v' if i'th case droppe
> d"
  8. }
(129 missing values generated)
(129 missing values generated)
(129 missing values generated)
(129 missing values generated)
(129 missing values generated)
(129 missing values generated)

.
. quietly: reg lr_eco_broad wdi_export_l1 wdi_import_l1 ///
>         po_mean_l1 wdi_gdpgr_l1 wdi_gdpcapcur_l1 lr_eco_broad_l1, cluster(countrynam
> e)

. predict resid if e(sample), resid

. sort resid

. gen casewise = _n if resid != .

. label var casewise "Help var for case-wise deletion"

. drop resid

. xtset panelvar_id timevar_id
       panel variable:  panelvar_id (unbalanced)
        time variable:  timevar_id, 1 to 15
                delta:  1 unit

. foreach v of varlist wdi_export_l1 wdi_import_l1 {
  2.         forvalues n = 1/129 {
  3.                 quietly: cluster2 lr_eco_broad wdi_export_l1 wdi_import_l1 po_mea
> n_l1 ///
>               wdi_gdpgr_l1 wdi_gdpcapcur_l1 lr_eco_broad_l1 if casewise != `n', //
> /
>               fcluster(countryname) tcluster(year)
  4.                 replace me_casew_`v'_full = _b[`v'] in `n'
  5.                 replace upp_casew_`v'_full_95 = _b[`v']+1.96*_se[`v'] in `n'
  6.                 replace low_casew_`v'_full_95 = _b[`v']-1.96*_se[`v'] in `n'
  7.         }
  8. }
(1 real change made)
(1 real change made)
(1 real change made)
(1 real change made)
(1 real change made)
(1 real change made)
(1 real change made)
(1 real change made)
(1 real change made)
(1 real change made)
(1 real change made)
(1 real change made)
(1 real change made)
(1 real change made)
(1 real change made)
(1 real change made)
(1 real change made)
(1 real change made)
(1 real change made)
(1 real change made)
(1 real change made)
```

```
(1 real change made)
(1 real change made)
(1 real change made)
(1 real change made)
(1 real change made)
(1 real change made)
(1 real change made)
(1 real change made)
(1 real change made)
(1 real change made)
(1 real change made)
(1 real change made)
(1 real change made)
(1 real change made)
(1 real change made)
(1 real change made)
(1 real change made)
(1 real change made)
(1 real change made)
(1 real change made)
(1 real change made)
(1 real change made)
(1 real change made)
(1 real change made)
(1 real change made)
(1 real change made)
(1 real change made)
(1 real change made)
(1 real change made)
(1 real change made)
(1 real change made)
(1 real change made)
(1 real change made)
(1 real change made)
(1 real change made)
(1 real change made)
(1 real change made)
(1 real change made)
(1 real change made)
(1 real change made)
(1 real change made)
(1 real change made)
(1 real change made)
(1 real change made)
(1 real change made)
(1 real change made)
(1 real change made)
(1 real change made)
(1 real change made)
(1 real change made)
(1 real change made)
(1 real change made)
(1 real change made)
(1 real change made)
(1 real change made)
(1 real change made)
(1 real change made)
(1 real change made)
(1 real change made)
(1 real change made)
(1 real change made)
(1 real change made)
(1 real change made)
(1 real change made)
(1 real change made)
(1 real change made)
(1 real change made)
(1 real change made)
(1 real change made)
(1 real change made)
(1 real change made)
(1 real change made)
```

```
(1 real change made)
(1 real change made)
(1 real change made)
(1 real change made)
(1 real change made)
(1 real change made)
(1 real change made)
(1 real change made)
(1 real change made)
(1 real change made)
(1 real change made)
(1 real change made)
(1 real change made)
(1 real change made)
(1 real change made)
(1 real change made)
(1 real change made)
(1 real change made)
(1 real change made)
(1 real change made)
(1 real change made)
(1 real change made)
(1 real change made)
(1 real change made)
(1 real change made)
(1 real change made)
(1 real change made)
(1 real change made)
(1 real change made)
(1 real change made)
(1 real change made)
(1 real change made)
(1 real change made)
(1 real change made)
(1 real change made)
(1 real change made)
(1 real change made)
(1 real change made)
(1 real change made)
(1 real change made)
(1 real change made)
(1 real change made)
(1 real change made)
(1 real change made)
(1 real change made)
(1 real change made)
(1 real change made)
(1 real change made)
(1 real change made)
(1 real change made)
(1 real change made)
(1 real change made)
(1 real change made)
(1 real change made)
(1 real change made)
(1 real change made)
(1 real change made)
(1 real change made)
(1 real change made)
(1 real change made)
(1 real change made)
(1 real change made)
(1 real change made)
(1 real change made)
(1 real change made)
(1 real change made)
(1 real change made)
(1 real change made)
(1 real change made)
(1 real change made)
(1 real change made)
(1 real change made)
```

```
(1 real change made)
(1 real change made)
(1 real change made)
(1 real change made)
(1 real change made)
(1 real change made)
(1 real change made)
(1 real change made)
(1 real change made)
(1 real change made)
(1 real change made)
(1 real change made)
(1 real change made)
(1 real change made)
(1 real change made)
(1 real change made)
(1 real change made)
(1 real change made)
(1 real change made)
(1 real change made)
(1 real change made)
(1 real change made)
(1 real change made)
(1 real change made)
(1 real change made)
(1 real change made)
(1 real change made)
(1 real change made)
(1 real change made)
(1 real change made)
(1 real change made)
(1 real change made)
(1 real change made)
(1 real change made)
(1 real change made)
(1 real change made)
(1 real change made)
(1 real change made)
(1 real change made)
(1 real change made)
(1 real change made)
(1 real change made)
(1 real change made)
(1 real change made)
(1 real change made)
(1 real change made)
(1 real change made)
(1 real change made)
(1 real change made)
(1 real change made)
(1 real change made)
(1 real change made)
(1 real change made)
(1 real change made)
(1 real change made)
(1 real change made)
(1 real change made)
(1 real change made)
(1 real change made)
(1 real change made)
(1 real change made)
(1 real change made)
(1 real change made)
(1 real change made)
(1 real change made)
(1 real change made)
(1 real change made)
(1 real change made)
(1 real change made)
(1 real change made)
(1 real change made)
(1 real change made)
```

```
(1 real change made)
(1 real change made)
(1 real change made)
(1 real change made)
(1 real change made)
(1 real change made)
(1 real change made)
(1 real change made)
(1 real change made)
(1 real change made)
(1 real change made)
(1 real change made)
(1 real change made)
(1 real change made)
(1 real change made)
(1 real change made)
(1 real change made)
(1 real change made)
(1 real change made)
(1 real change made)
(1 real change made)
(1 real change made)
(1 real change made)
(1 real change made)
(1 real change made)
(1 real change made)
(1 real change made)
(1 real change made)
(1 real change made)
(1 real change made)
(1 real change made)
(1 real change made)
(1 real change made)
(1 real change made)
(1 real change made)
(1 real change made)
(1 real change made)
(1 real change made)
(1 real change made)
(1 real change made)
(1 real change made)
(1 real change made)
(1 real change made)
(1 real change made)
(1 real change made)
(1 real change made)
(1 real change made)
(1 real change made)
(1 real change made)
(1 real change made)
(1 real change made)
(1 real change made)
(1 real change made)
(1 real change made)
(1 real change made)
(1 real change made)
(1 real change made)
(1 real change made)
(1 real change made)
(1 real change made)
(1 real change made)
(1 real change made)
(1 real change made)
(1 real change made)
(1 real change made)
(1 real change made)
(1 real change made)
(1 real change made)
(1 real change made)
(1 real change made)
(1 real change made)
(1 real change made)
```

```
(1 real change made)
(1 real change made)
(1 real change made)
(1 real change made)
(1 real change made)
(1 real change made)
(1 real change made)
(1 real change made)
(1 real change made)
(1 real change made)
(1 real change made)
(1 real change made)
(1 real change made)
(1 real change made)
(1 real change made)
(1 real change made)
(1 real change made)
(1 real change made)
(1 real change made)
(1 real change made)
(1 real change made)
(1 real change made)
(1 real change made)
(1 real change made)
(1 real change made)
(1 real change made)
(1 real change made)
(1 real change made)
(1 real change made)
(1 real change made)
(1 real change made)
(1 real change made)
(1 real change made)
(1 real change made)
(1 real change made)
(1 real change made)
(1 real change made)
(1 real change made)
(1 real change made)
(1 real change made)
(1 real change made)
(1 real change made)
(1 real change made)
(1 real change made)
(1 real change made)
(1 real change made)
(1 real change made)
(1 real change made)
(1 real change made)
(1 real change made)
(1 real change made)
(1 real change made)
(1 real change made)
(1 real change made)
(1 real change made)
(1 real change made)
(1 real change made)
(1 real change made)
(1 real change made)
(1 real change made)
(1 real change made)
(1 real change made)
(1 real change made)
(1 real change made)
(1 real change made)
(1 real change made)
(1 real change made)
(1 real change made)
(1 real change made)
(1 real change made)
(1 real change made)
(1 real change made)
(1 real change made)
(1 real change made)
(1 real change made)
(1 real change made)
```

```
(1 real change made)
(1 real change made)
(1 real change made)
(1 real change made)
(1 real change made)
(1 real change made)
(1 real change made)
(1 real change made)
(1 real change made)
(1 real change made)
(1 real change made)
(1 real change made)
(1 real change made)
(1 real change made)
(1 real change made)
(1 real change made)
(1 real change made)
(1 real change made)
(1 real change made)
(1 real change made)
(1 real change made)
(1 real change made)
(1 real change made)
(1 real change made)
(1 real change made)
(1 real change made)
(1 real change made)
(1 real change made)
(1 real change made)
(1 real change made)
(1 real change made)
(1 real change made)
(1 real change made)
(1 real change made)
(1 real change made)
(1 real change made)
(1 real change made)
(1 real change made)
(1 real change made)
(1 real change made)
(1 real change made)
(1 real change made)
(1 real change made)
(1 real change made)
(1 real change made)
(1 real change made)
(1 real change made)
(1 real change made)
(1 real change made)
(1 real change made)
(1 real change made)
(1 real change made)
(1 real change made)
(1 real change made)
(1 real change made)
(1 real change made)
(1 real change made)
(1 real change made)
(1 real change made)
(1 real change made)
(1 real change made)
(1 real change made)
(1 real change made)
(1 real change made)
(1 real change made)
(1 real change made)
(1 real change made)
(1 real change made)
(1 real change made)
(1 real change made)
(1 real change made)
(1 real change made)
```

```
(1 real change made)
(1 real change made)
(1 real change made)
(1 real change made)
(1 real change made)
(1 real change made)
(1 real change made)
(1 real change made)
(1 real change made)
(1 real change made)
(1 real change made)
(1 real change made)
(1 real change made)
(1 real change made)
(1 real change made)
(1 real change made)
(1 real change made)
(1 real change made)
(1 real change made)
(1 real change made)
(1 real change made)
(1 real change made)
(1 real change made)
(1 real change made)
(1 real change made)
(1 real change made)
(1 real change made)
(1 real change made)
(1 real change made)
(1 real change made)
(1 real change made)
(1 real change made)
(1 real change made)
(1 real change made)
(1 real change made)
(1 real change made)
(1 real change made)
(1 real change made)
(1 real change made)
(1 real change made)
(1 real change made)
(1 real change made)
(1 real change made)
(1 real change made)
(1 real change made)
(1 real change made)
(1 real change made)
(1 real change made)
(1 real change made)
(1 real change made)
(1 real change made)
(1 real change made)
(1 real change made)
(1 real change made)
(1 real change made)
(1 real change made)
(1 real change made)
(1 real change made)
(1 real change made)
(1 real change made)
(1 real change made)
(1 real change made)
(1 real change made)
(1 real change made)
(1 real change made)
(1 real change made)
(1 real change made)
(1 real change made)
(1 real change made)
(1 real change made)
(1 real change made)
(1 real change made)
```

```
(1 real change made)
(1 real change made)
(1 real change made)
(1 real change made)
(1 real change made)
(1 real change made)
(1 real change made)
(1 real change made)
(1 real change made)
(1 real change made)
(1 real change made)
(1 real change made)
(1 real change made)
(1 real change made)
(1 real change made)
(1 real change made)
(1 real change made)
(1 real change made)
(1 real change made)
(1 real change made)
(1 real change made)
(1 real change made)
(1 real change made)
(1 real change made)
(1 real change made)
(1 real change made)
(1 real change made)
(1 real change made)
(1 real change made)
(1 real change made)
(1 real change made)
(1 real change made)
(1 real change made)
(1 real change made)
(1 real change made)
(1 real change made)
(1 real change made)
(1 real change made)
(1 real change made)
(1 real change made)
(1 real change made)
(1 real change made)
(1 real change made)
(1 real change made)
(1 real change made)
(1 real change made)
(1 real change made)
(1 real change made)
(1 real change made)
(1 real change made)
(1 real change made)
(1 real change made)
(1 real change made)
(1 real change made)
(1 real change made)
(1 real change made)
(1 real change made)
(1 real change made)
(1 real change made)
(1 real change made)
(1 real change made)
(1 real change made)
(1 real change made)
(1 real change made)
(1 real change made)
(1 real change made)
(1 real change made)
(1 real change made)
(1 real change made)
(1 real change made)
(1 real change made)
(1 real change made)
```

```
(1 real change made)
(1 real change made)
(1 real change made)
(1 real change made)
(1 real change made)
(1 real change made)
(1 real change made)
(1 real change made)
(1 real change made)
(1 real change made)
(1 real change made)
(1 real change made)
(1 real change made)
(1 real change made)
(1 real change made)
(1 real change made)
(1 real change made)
(1 real change made)
(1 real change made)
(1 real change made)
(1 real change made)
(1 real change made)
(1 real change made)
(1 real change made)
(1 real change made)
(1 real change made)
(1 real change made)
(1 real change made)
(1 real change made)
(1 real change made)
(1 real change made)
(1 real change made)
(1 real change made)
(1 real change made)
(1 real change made)
(1 real change made)
(1 real change made)
(1 real change made)
(1 real change made)
(1 real change made)
(1 real change made)
(1 real change made)
(1 real change made)
(1 real change made)
(1 real change made)
(1 real change made)
(1 real change made)
(1 real change made)
(1 real change made)
(1 real change made)
(1 real change made)
(1 real change made)
(1 real change made)
(1 real change made)
(1 real change made)
(1 real change made)
(1 real change made)
(1 real change made)
(1 real change made)
(1 real change made)
(1 real change made)
(1 real change made)
(1 real change made)
(1 real change made)
(1 real change made)
(1 real change made)
(1 real change made)
(1 real change made)
(1 real change made)
(1 real change made)
(1 real change made)
(1 real change made)
```

```
(1 real change made)
(1 real change made)
(1 real change made)
(1 real change made)
(1 real change made)
(1 real change made)
(1 real change made)
(1 real change made)
(1 real change made)
(1 real change made)
(1 real change made)
(1 real change made)
(1 real change made)
(1 real change made)
(1 real change made)
(1 real change made)
(1 real change made)
(1 real change made)
(1 real change made)
(1 real change made)
(1 real change made)
(1 real change made)
(1 real change made)
(1 real change made)
(1 real change made)
(1 real change made)
(1 real change made)
(1 real change made)
(1 real change made)
(1 real change made)
(1 real change made)
(1 real change made)
(1 real change made)
(1 real change made)
(1 real change made)
(1 real change made)
(1 real change made)
(1 real change made)
(1 real change made)
(1 real change made)
(1 real change made)
(1 real change made)
(1 real change made)
(1 real change made)
(1 real change made)
(1 real change made)
(1 real change made)
(1 real change made)
(1 real change made)
(1 real change made)
(1 real change made)
(1 real change made)
(1 real change made)
(1 real change made)
(1 real change made)
(1 real change made)
(1 real change made)
(1 real change made)
(1 real change made)
(1 real change made)
(1 real change made)
(1 real change made)
(1 real change made)
(1 real change made)
(1 real change made)
(1 real change made)
(1 real change made)
(1 real change made)
(1 real change made)
(1 real change made)
(1 real change made)
(1 real change made)
```

```
(1 real change made)
(1 real change made)
(1 real change made)
(1 real change made)
(1 real change made)
(1 real change made)
(1 real change made)
(1 real change made)
(1 real change made)
(1 real change made)
(1 real change made)
(1 real change made)
(1 real change made)
(1 real change made)
(1 real change made)
(1 real change made)
(1 real change made)
(1 real change made)
(1 real change made)
(1 real change made)
(1 real change made)
(1 real change made)
(1 real change made)
(1 real change made)
(1 real change made)
(1 real change made)
(1 real change made)
(1 real change made)
(1 real change made)
(1 real change made)
(1 real change made)
(1 real change made)
(1 real change made)

.
. * S3 Fig
. scatter me_casew_wdi_import_l1_full casewise, ///
>         mc(gs0) msize(small) ///
>         legend(off) graphregion(color(gs16)) || ///
> rspike upp_casew_wdi_import_l1_full_95 low_casew_wdi_import_l1_full_95 casewise, ///
>         lc(gs0) lwidth(vthin) ///
>         xlabel(1 25 50 75 100 125, labsize(medium)) xtitle(" " "Case ID", size(mediu
> m)) ///
>         ytitle("Marginal effect and 95% CI for imports" " ", size(medium)) ///
>         yline(0, lp(dash) lc(gs0)) ylabel(, labsize(medium)) ///
>         legend(off)

. graph save casewise_imports_full_95, replace
(note: file casewise_imports_full_95.gph not found)
(file casewise_imports_full_95.gph saved)

. graph export s3_fig.eps, replace
(note: file s3_fig.eps not found)
(file s3_fig.eps written in EPS format)

.
. * S4 Fig
. scatter me_casew_wdi_export_l1_full casewise, ///
>         mc(gs0) msize(small) ///
>         legend(off) graphregion(color(gs16)) || ///
> rspike upp_casew_wdi_export_l1_full_95 low_casew_wdi_export_l1_full_95 casewise, ///
>         lc(gs0) lwidth(vthin) ///
>         xlabel(1 25 50 75 100 125, labsize(medium)) xtitle(" " "Case ID", size(mediu
> m)) ///
>         ytitle("Marginal effect and 95% CI for exports" " ", ///
>         size(mediumsmall)) yline(0, lp(dash) lc(gs0)) ylabel(, labsize(medium)) ///
>         legend(off)
(note:  named style mediumsmall not found in class gsize, default attributes used)
```

```
. graph save casewise_exports_full_95, replace
(note: file casewise_exports_full_95.gph not found)
(file casewise_exports_full_95.gph saved)

. graph export s4_fig.eps, replace
(note: file s4_fig.eps not found)
(file s4_fig.eps written in EPS format)

.
.
. *** Predicted outcomes for levels of imports and exports
. /* We use one-way clustering by country to be able to use margins */
. quietly: reg lr_eco_broad wdi_export_l1 wdi_import_l1 po_mean_l1 ///
>         wdi_gdpgr_l1 wdi_gdpcapcur_l1 lr_eco_broad_l1, cluster(countryname)

.
. * Fig 6
. margins, at(wdi_import_l1=(20(5)70) wdi_export_l1=(50))

Predictive margins                              Number of obs     =        129
Model VCE    : Robust

Expression   : Linear prediction, predict()

1._at        : wdi_export~1    =          50
               wdi_import~1    =          20

2._at        : wdi_export~1    =          50
               wdi_import~1    =          25

3._at        : wdi_export~1    =          50
               wdi_import~1    =          30

4._at        : wdi_export~1    =          50
               wdi_import~1    =          35

5._at        : wdi_export~1    =          50
               wdi_import~1    =          40

6._at        : wdi_export~1    =          50
               wdi_import~1    =          45

7._at        : wdi_export~1    =          50
               wdi_import~1    =          50

8._at        : wdi_export~1    =          50
               wdi_import~1    =          55

9._at        : wdi_export~1    =          50
               wdi_import~1    =          60

10._at       : wdi_export~1    =          50
               wdi_import~1    =          65

11._at       : wdi_export~1    =          50
               wdi_import~1    =          70

------------------------------------------------------------------------------
             |            Delta-method
             |     Margin   Std. Err.      t    P>|t|     [95% Conf. Interval]
-------------+----------------------------------------------------------------
         _at |
          1  |  -.3053747   .0647201    -4.72   0.000    -.4441855   -.1665639
          2  |  -.3375558   .0537605    -6.28   0.000    -.4528607   -.2222509
          3  |   -.369737   .0428756    -8.62   0.000    -.4616959   -.2777781
          4  |  -.4019181    .032141   -12.50   0.000    -.4708536   -.3329826
          5  |  -.4340993   .0217803   -19.93   0.000    -.4808133   -.3873853
          6  |  -.4662804   .0127408   -36.60   0.000    -.4936067   -.4389541
          7  |  -.4984616   .0098431   -50.64   0.000     -.519573   -.4773502
          8  |  -.5306427    .016675   -31.82   0.000    -.5664071   -.4948784
          9  |  -.5628239   .0265676   -21.18   0.000    -.6198057   -.5058421
         10  |   -.595005   .0371514   -16.02   0.000    -.6746869   -.5153232
```

```
        11  |  -.6271862   .0479711   -13.07   0.000     -.730074   -.5242984
------------------------------------------------------------------------------

. marginsplot, xtitle("Level of imports") xlabel(20(5)70) xscale(titlegap(*10)) ///
>         ytitle("Predicted center of gravity") ylabel(-0.8(0.1)-0.1, angle(0)) yscale
> (titlegap(*10)) /
> //
>         title("") graphregion(color(gs16)) plotopts(mc(gs0) c(i)) ///
>         ciopts(lcolor(gs0))

  Variables that uniquely identify margins: wdi_import_l1

. graph save predimports, replace
(note: file predimports.gph not found)
(file predimports.gph saved)

. graph export fig6.eps, replace
(note: file fig6.eps not found)
(file fig6.eps written in EPS format)

.
. * Fig 7
. margins, at(wdi_export_l1=(20(5)70) wdi_import_l1=(50))

Predictive margins                              Number of obs     =        129
Model VCE    : Robust

Expression   : Linear prediction, predict()

1._at        : wdi_export~1    =          20
               wdi_import~1    =          50

2._at        : wdi_export~1    =          25
               wdi_import~1    =          50

3._at        : wdi_export~1    =          30
               wdi_import~1    =          50

4._at        : wdi_export~1    =          35
               wdi_import~1    =          50

5._at        : wdi_export~1    =          40
               wdi_import~1    =          50

6._at        : wdi_export~1    =          45
               wdi_import~1    =          50

7._at        : wdi_export~1    =          50
               wdi_import~1    =          50

8._at        : wdi_export~1    =          55
               wdi_import~1    =          50

9._at        : wdi_export~1    =          60
               wdi_import~1    =          50

10._at       : wdi_export~1    =          65
               wdi_import~1    =          50

11._at       : wdi_export~1    =          70
               wdi_import~1    =          50
```

```
------------------------------------------------------------------------------
             |            Delta-method
             |     Margin   Std. Err.      t    P>|t|     [95% Conf. Interval]
-------------+----------------------------------------------------------------
         _at |
          1  |  -.6687649   .0574176   -11.65   0.000    -.7919134   -.5456163
          2  |   -.640381   .0485573   -13.19   0.000     -.744526   -.5362359
          3  |  -.6119971   .0397631   -15.39   0.000    -.6972804   -.5267138
          4  |  -.5836132   .0310911   -18.77   0.000    -.6502969   -.5169295
          5  |  -.5552293   .0226819   -24.48   0.000    -.6038772   -.5065815
          6  |  -.5268455   .0149849   -35.16   0.000    -.5589848   -.4947061
          7  |  -.4984616   .0098431   -50.64   0.000     -.519573   -.4773502
          8  |  -.4700777   .0114669   -40.99   0.000    -.4946718   -.4454836
          9  |  -.4416938   .0181206   -24.38   0.000    -.4805586    -.402829
         10  |  -.4133099   .0262199   -15.76   0.000    -.4695461   -.3570738
         11  |  -.3849261   .0347689   -11.07   0.000    -.4594979   -.3103542
------------------------------------------------------------------------------

. marginsplot, xtitle("Level of exports") xlabel(20(5)70) xscale(titlegap(*10)) ///
>         ytitle("Predicted center of gravity") ylabel(-.9(0.1)-0.2, angle(0)) yscale(
> titlegap(*10)) //
> /
>         title("") graphregion(color(gs16)) plotopts(mc(gs0) c(i)) ///
>         ciopts(lcolor(gs0))

  Variables that uniquely identify margins: wdi_export_l1

. graph save predexports, replace
(note: file predexports.gph not found)
(file predexports.gph saved)

. graph export fig7.eps, replace
(note: file fig7.eps not found)
(file fig7.eps written in EPS format)

.
. sum wdi_import_l1, d // Displaying percentiles

                  wdi_import w one-year lag
-------------------------------------------------------------
      Percentiles      Smallest
 1%     17.40621       16.88713
 5%     19.64142       17.40621
10%      21.7402       17.50577       Obs                 129
25%     27.50004       18.71774       Sum of Wgt.         129

50%     35.24374                      Mean            42.1822
                        Largest       Std. Dev.      23.69967
75%     52.71184       104.7872
90%     68.81677       116.8144       Variance       561.6745
95%     79.82157       158.8848       Skewness       2.384934
99%     158.8848       159.3568       Kurtosis       11.09486

. quietly: sum wdi_import_l1 if wdi_import_l1 >= 20 & wdi_import_l1 <= 70

. display as text "Range of 20 to 70 covers " r(N) " observations."
Range of 20 to 70 covers 108 observations.

.
.
```

```
. *** Correlation Matrix DV
. * Table 3
. correlate lr_eco_broad lr_eco_lb lr_eco_bl lr_eco_t lr_eco_bh lr_eco_p
(obs=129)

             | lr_eco~d lr_eco~b lr_eco~l lr_eco_t lr_eco~h lr_eco_p
-------------+------------------------------------------------------
lr_eco_broad |   1.0000
   lr_eco_lb |   0.4543   1.0000
   lr_eco_bl |   0.8989   0.5378   1.0000
    lr_eco_t |   0.4825   0.9658   0.5526   1.0000
   lr_eco_bh |   0.9711   0.4719   0.9173   0.4991   1.0000
    lr_eco_p |   0.8800   0.4163   0.8584   0.4365   0.8132   1.0000


.
. *** Comparison different constructions of economic dimension
. cluster2 lr_eco_broad wdi_export_l1 wdi_import_l1 po_mean_l1 ///
>         wdi_gdpgr_l1 wdi_gdpcapcur_l1 lr_eco_broad_l1, fcluster(countryname) tcluste
> r(year)

Linear regression with 2D clustered SEs                Number of obs =     129
                                                       F(  6,   124) =   14.69
                                                       Prob > F      =  0.0000
Number of clusters (countryname) =    15               R-squared     =  0.3896
Number of clusters (year) =     38                     Root MSE      =  0.1449
------------------------------------------------------------------------------
    lr_eco_broad |      Coef.   Std. Err.      t    P>|t|     [95% Conf. Interval]
-----------------+------------------------------------------------------------
   wdi_export_l1 |   .0056768   .0021612     2.63   0.010     .0013992    .0099543
   wdi_import_l1 |  -.0064362   .0026401    -2.44   0.016    -.0116618   -.0012106
      po_mean_l1 |    .087551   .0349771     2.50   0.014     .0183216    .1567805
    wdi_gdpgr_l1 |  -.0218497   .0032979    -6.63   0.000    -.0283771   -.0153223
wdi_gdpcapcur_l1 |  -3.59e-06   1.12e-06    -3.20   0.002    -5.81e-06   -1.37e-06
 lr_eco_broad_l1 |   .3442217   .0531408     6.48   0.000     .2390413    .4494022
           _cons |  -.6347477    .193572    -3.28   0.001    -1.017881   -.2516144
------------------------------------------------------------------------------

     SE clustered by countryname and year (multiple obs per countryname-year)


. est sto broad

. cluster2 lr_eco_lb wdi_export_l1 wdi_import_l1 po_mean_l1 ///
>         wdi_gdpgr_l1 wdi_gdpcapcur_l1 lr_eco_broad_l1, fcluster(countryname) tcluste
> r(year)

Linear regression with 2D clustered SEs                Number of obs =     129
                                                       F(  6,   124) =    5.69
                                                       Prob > F      =  0.0000
Number of clusters (countryname) =    15               R-squared     =  0.2119
Number of clusters (year) =     38                     Root MSE      =  0.2377
------------------------------------------------------------------------------
       lr_eco_lb |      Coef.   Std. Err.      t    P>|t|     [95% Conf. Interval]
-----------------+------------------------------------------------------------
   wdi_export_l1 |   .0151225   .0031363     4.82   0.000     .0089149    .0213301
   wdi_import_l1 |  -.0188825   .0040709    -4.64   0.000      -.02694    -.010825
      po_mean_l1 |   .1821657   .0571234     3.19   0.002     .0691025    .2952289
    wdi_gdpgr_l1 |  -.0123444   .0090054    -1.37   0.173    -.0301686    .0054797
wdi_gdpcapcur_l1 |  -8.15e-07   1.68e-06    -0.49   0.627    -4.13e-06    2.50e-06
 lr_eco_broad_l1 |   .2556139   .1738837     1.47   0.144    -.0885507    .5997785
           _cons |  -.4675921   .3043949    -1.54   0.127    -1.070075    .1348906
------------------------------------------------------------------------------

     SE clustered by countryname and year (multiple obs per countryname-year)
```

```
. est sto lb

. cluster2 lr_eco_bl wdi_export_l1 wdi_import_l1 po_mean_l1 ///
>         wdi_gdpgr_l1 wdi_gdpcapcur_l1 lr_eco_broad_l1, fcluster(countryname) tcluste
> r(year)

Linear regression with 2D clustered SEs                Number of obs =     129
                                                       F(  6,   124) =   13.51
                                                       Prob > F      =  0.0000
Number of clusters (countryname) =    15               R-squared     =  0.3571
Number of clusters (year) =      38                    Root MSE      =  0.1616
------------------------------------------------------------------------------
       lr_eco_bl |      Coef.   Std. Err.      t    P>|t|     [95% Conf. Interval]
-----------------+------------------------------------------------------------
   wdi_export_l1 |   .0098075   .0024349     4.03   0.000     .0049881     .014627
   wdi_import_l1 |  -.0109021   .0028438    -3.83   0.000    -.0165309   -.0052734
      po_mean_l1 |   .1058235   .0409129     2.59   0.011     .0248454    .1868016
    wdi_gdpgr_l1 |  -.0253084   .0051681    -4.90   0.000    -.0355375   -.0150793
wdi_gdpcapcur_l1 |  -3.53e-06   1.54e-06    -2.29   0.023    -6.57e-06   -4.85e-07
 lr_eco_broad_l1 |   .3440575   .0734766     4.68   0.000     .1986267    .4894883
           _cons |  -.6612245    .210132    -3.15   0.002    -1.077135   -.2453145
------------------------------------------------------------------------------

     SE clustered by countryname and year (multiple obs per countryname-year)


. est sto bl

. cluster2 lr_eco_t wdi_export_l1 wdi_import_l1 po_mean_l1 ///
>         wdi_gdpgr_l1 wdi_gdpcapcur_l1 lr_eco_broad_l1, fcluster(countryname) tcluste
> r(year)

Linear regression with 2D clustered SEs                Number of obs =     129
                                                       F(  6,   124) =    6.91
                                                       Prob > F      =  0.0000
Number of clusters (countryname) =    15               R-squared     =  0.2345
Number of clusters (year) =      38                    Root MSE      =  0.2386
------------------------------------------------------------------------------
        lr_eco_t |      Coef.   Std. Err.      t    P>|t|     [95% Conf. Interval]
-----------------+------------------------------------------------------------
   wdi_export_l1 |   .0129843   .0025142     5.16   0.000     .0080081    .0179605
   wdi_import_l1 |  -.0167709   .0032457    -5.17   0.000     -.023195   -.0103468
      po_mean_l1 |   .2161079   .0453588     4.76   0.000     .1263301    .3058857
    wdi_gdpgr_l1 |  -.0171415   .0069364    -2.47   0.015    -.0308705   -.0034125
wdi_gdpcapcur_l1 |  -6.73e-07   1.83e-06    -0.37   0.714    -4.30e-06    2.95e-06
 lr_eco_broad_l1 |   .2834828   .1679976     1.69   0.094    -.0490316    .6159972
           _cons |   -.654389   .2371715    -2.76   0.007    -1.123818   -.1849602
------------------------------------------------------------------------------

     SE clustered by countryname and year (multiple obs per countryname-year)


. est sto t

. cluster2 lr_eco_bh wdi_export_l1 wdi_import_l1 po_mean_l1 ///
>         wdi_gdpgr_l1 wdi_gdpcapcur_l1 lr_eco_broad_l1, fcluster(countryname) tcluste
> r(year)

Linear regression with 2D clustered SEs                Number of obs =     129
                                                       F(  6,   124) =   13.16
                                                       Prob > F      =  0.0000
Number of clusters (countryname) =    15               R-squared     =  0.3695
Number of clusters (year) =      38                    Root MSE      =  0.1572
------------------------------------------------------------------------------
       lr_eco_bh |      Coef.   Std. Err.      t    P>|t|     [95% Conf. Interval]
-----------------+------------------------------------------------------------
   wdi_export_l1 |   .0062929   .0022325     2.82   0.006     .0018741    .0107117
   wdi_import_l1 |   -.006986   .0027355    -2.55   0.012    -.0124003   -.0015716
      po_mean_l1 |   .0653343   .0380946     1.72   0.089    -.0100655    .1407341
    wdi_gdpgr_l1 |  -.0212588   .0046223    -4.60   0.000    -.0304076   -.0121099
wdi_gdpcapcur_l1 |  -4.17e-06   1.34e-06    -3.10   0.002    -6.83e-06   -1.51e-06
 lr_eco_broad_l1 |   .3711389   .0807222     4.60   0.000      .211367    .5309108
```

```
          _cons |  -.4170698   .2018481    -2.07   0.041    -.8165836   -.0175559
------------------------------------------------------------------------------

     SE clustered by countryname and year (multiple obs per countryname-year)


. est sto bh

. cluster2 lr_eco_p wdi_export_l1 wdi_import_l1 po_mean_l1 ///
>         wdi_gdpgr_l1 wdi_gdpcapcur_l1 lr_eco_broad_l1, fcluster(countryname) tcluste
> r(year)

Linear regression with 2D clustered SEs               Number of obs =     129
                                                      F(  6,   124) =   10.42
                                                      Prob > F      =  0.0000
Number of clusters (countryname) =    15              R-squared     =  0.3185
Number of clusters (year) =     38                    Root MSE      =  0.1398
------------------------------------------------------------------------------
        lr_eco_p |      Coef.   Std. Err.      t    P>|t|     [95% Conf. Interval]
-----------------+------------------------------------------------------------
   wdi_export_l1 |   .0064567    .001849     3.49   0.001     .0027969    .0101164
   wdi_import_l1 |  -.0076331   .0022042    -3.46   0.001    -.0119959   -.0032704
      po_mean_l1 |    .092913   .0407515     2.28   0.024     .0122544    .1735715
    wdi_gdpgr_l1 |  -.0215378   .0040748    -5.29   0.000     -.029603   -.0134726
wdi_gdpcapcur_l1 |  -2.11e-06   1.03e-06    -2.05   0.043    -4.15e-06   -7.12e-08
 lr_eco_broad_l1 |   .2710015   .0105948    25.58   0.000     .2500314    .2919717
           _cons |  -.8898023   .2091467    -4.25   0.000    -1.303762   -.4758424
------------------------------------------------------------------------------

     SE clustered by countryname and year (multiple obs per countryname-year)


. est sto p

.
. * Summarizing results for different outcome measures
. * S3 Table
. esttab broad lb bl t bh p using ///
>         dv_reg_table.rtf, replace ///
>         cells(b(star fmt(%9.4f)) se(par)) stats(r2_a N, fmt(2 0)) varwidth(10) ///
>         modelwidth(9) order(wdi_import_l1 wdi_export_l1 po_mean_l1 ///
>         wdi_gdpgr_l1 wdi_gdpcapcur_l1 lr_eco_broad_l1 _cons) ///
>         varlabels(wdi_import_l1 "Imports" wdi_export_l1 "Exports" ///
>         po_mean_l1 "Median voter" wdi_gdpgr_l1 "GDP growth" ///
>         wdi_gdpcapcur_l1 "GDP/capita" lr_eco_broad_l1 "Lagged DV" _cons "Constant")
> ///
>         addnote("Standard errors in parentheses; two-sided tests; p < .05 *; p < .01
>  **; p < .001 ***
> ") ///
>         nonumbers nolines
(note: file dv_reg_table.rtf not found)
(output written to dv_reg_table.rtf)

.
. * Model with country and period fixed effects
. // We use r2 and not adjusted r2 because it is not computed
. xtset panelvar_id timevar_id
       panel variable:  panelvar_id (unbalanced)
        time variable:  timevar_id, 1 to 15
                delta:  1 unit
```

```
. * Baseline + country FE
. xi: xtreg lr_eco_broad wdi_export_l1 wdi_import_l1 po_mean_l1 ///
>         wdi_gdpgr_l1 wdi_gdpcapcur_l1 lr_eco_broad_l1 i.countryname
i.countryname     _Icountryna_1-15    (_Icountryna_1 for cou~e==Austria omitted)

Random-effects GLS regression                   Number of obs     =        129
Group variable: panelvar_id                     Number of groups  =         15

R-sq:                                           Obs per group:
     within  = 0.2914                                         min =          2
     between = 1.0000                                         avg =        8.6
     overall = 0.4501                                         max =         15

                                                Wald chi2(20)     =      88.41
corr(u_i, X)   = 0 (assumed)                    Prob > chi2       =     0.0000

------------------------------------------------------------------------------
    lr_eco_broad |      Coef.   Std. Err.      z    P>|z|     [95% Conf. Interval]
-----------------+------------------------------------------------------------
   wdi_export_l1 |   .0060645   .0037001     1.64   0.101    -.0011875    .0133165
   wdi_import_l1 |  -.0049146   .0047159    -1.04   0.297    -.0141575    .0043283
      po_mean_l1 |   .0639001   .0590459     1.08   0.279    -.0518277    .1796279
    wdi_gdpgr_l1 |  -.0205403   .0054625    -3.76   0.000    -.0312466   -.0098339
wdi_gdpcapcur_l1 |  -4.58e-06   1.81e-06    -2.53   0.011    -8.13e-06   -1.04e-06
 lr_eco_broad_l1 |   .2848274    .084636     3.37   0.001     .1189438    .4507109
   _Icountryna_2 |   .1245628   .1056293     1.18   0.238    -.0824669    .3315925
   _Icountryna_3 |   .0880674   .1356143     0.65   0.516    -.1777318    .3538666
   _Icountryna_4 |   .2282088   .0921332     2.48   0.013     .0476311    .4087865
   _Icountryna_5 |   .1898678   .1116021     1.70   0.089    -.0288683    .4086038
   _Icountryna_6 |   .1393108   .0921178     1.51   0.130    -.0412367    .3198584
   _Icountryna_7 |   .1770012   .0891159     1.99   0.047     .0023373    .3516651
   _Icountryna_8 |   .1442062   .1155983     1.25   0.212    -.0823623    .3707746
   _Icountryna_9 |   .2478541   .0963878     2.57   0.010     .0589376    .4367707
  _Icountryna_10 |   .0362847   .1646284     0.22   0.826    -.2863811    .3589505
  _Icountryna_11 |   .1026175   .0941784     1.09   0.276    -.0819688    .2872038
  _Icountryna_12 |   .1731735   .0972937     1.78   0.075    -.0175186    .3638656
  _Icountryna_13 |   .1395346   .0966157     1.44   0.149    -.0498288    .3288979
  _Icountryna_14 |   .1193413   .1132868     1.05   0.292    -.1026967    .3413793
  _Icountryna_15 |   .2170072   .0973876     2.23   0.026      .026131    .4078835
           _cons |    -.74962   .3327525    -2.25   0.024    -1.401803    -.097437
-----------------+------------------------------------------------------------
         sigma_u |          0
         sigma_e |  .14616034
             rho |          0   (fraction of variance due to u_i)
------------------------------------------------------------------------------

. eststo countryfe

.
. * Baseline + period FE
. xi: xtreg lr_eco_broad wdi_export_l1 wdi_import_l1 po_mean_l1 ///
>         wdi_gdpgr_l1 wdi_gdpcapcur_l1 lr_eco_broad_l1 i.year
i.year            _Iyear_1976-2015    (naturally coded; _Iyear_1976 omitted)

Random-effects GLS regression                   Number of obs     =        129
Group variable: panelvar_id                     Number of groups  =         15

R-sq:                                           Obs per group:
     within  = 0.5605                                         min =          2
     between = 0.7978                                         avg =        8.6
     overall = 0.6179                                         max =         15

                                                Wald chi2(43)     =     137.45
corr(u_i, X)   = 0 (assumed)                    Prob > chi2       =     0.0000
```

```
------------------------------------------------------------------------------
    lr_eco_broad |      Coef.   Std. Err.      z    P>|z|     [95% Conf. Interval]
-----------------+----------------------------------------------------------------
   wdi_export_l1 |   .0048959   .0035673     1.37   0.170     -.002096    .0118878
   wdi_import_l1 |  -.0054821   .0040867    -1.34   0.180    -.0134918    .0025276
      po_mean_l1 |    .073472   .0384018     1.91   0.056    -.0017942    .1487382
     wdi_gdpgr_l1 |   -.014944   .0082514    -1.81   0.070    -.0311164    .0012285
wdi_gdpcapcur_l1 |  -2.76e-06   2.24e-06    -1.23   0.219    -7.15e-06    1.64e-06
 lr_eco_broad_l1 |    .391474   .0823862     4.75   0.000     .2300001    .5529479
      _Iyear_1977 |   .1448379   .1307225     1.11   0.268    -.1113734    .4010492
      _Iyear_1978 |   .2554728   .1725743     1.48   0.139    -.0827665    .5937122
      _Iyear_1979 |   .1662779   .1255266     1.32   0.185    -.0797497    .4123055
      _Iyear_1980 |    .052466   .1768975     0.30   0.767    -.2942467    .3991788
      _Iyear_1981 |   .2501823   .1240062     2.02   0.044     .0071345    .4932301
      _Iyear_1982 |   .0605222   .1397392     0.43   0.665    -.2133615     .334406
      _Iyear_1983 |   .1442898    .128105     1.13   0.260    -.1067915     .395371
      _Iyear_1984 |   -.019414   .1468926    -0.13   0.895    -.3073182    .2684902
      _Iyear_1985 |   .2369987   .1452159     1.63   0.103    -.0476192    .5216165
      _Iyear_1986 |   .0679182   .1425517     0.48   0.634     -.211478    .3473143
      _Iyear_1987 |   .0353581   .1175342     0.30   0.764    -.1950046    .2657209
      _Iyear_1988 |   .3121388   .1720525     1.81   0.070    -.0250779    .6493555
      _Iyear_1989 |   .0546629   .1254004     0.44   0.663    -.1911175    .3004432
      _Iyear_1990 |   -.078644   .1336382    -0.59   0.556      -.34057     .183282
      _Iyear_1991 |  -.0368306   .1476563    -0.25   0.803    -.3262315    .2525703
      _Iyear_1992 |   .1217601   .1289772     0.94   0.345    -.1310304    .3745507
      _Iyear_1993 |   .1289463   .1431471     0.90   0.368    -.1516169    .4095094
      _Iyear_1994 |   .1952195   .1197724     1.63   0.103    -.0395302    .4299692
      _Iyear_1995 |   .1302404   .1321765     0.99   0.324    -.1288208    .3893016
      _Iyear_1996 |   .1622612   .1339517     1.21   0.226    -.1002792    .4248017
      _Iyear_1997 |   .1526333    .151185     1.01   0.313    -.1436839    .4489504
      _Iyear_1998 |   .1508576    .131279     1.15   0.250    -.1064446    .4081598
      _Iyear_1999 |  -.0436263   .1300979    -0.34   0.737    -.2986134    .2113608
      _Iyear_2000 |  -.0124244   .1484241    -0.08   0.933    -.3033303    .2784814
      _Iyear_2001 |   .0226376   .1385906     0.16   0.870     -.248995    .2942702
      _Iyear_2002 |   .0243989   .1200953     0.20   0.839    -.2109836    .2597814
      _Iyear_2003 |  -.0204588   .1298953    -0.16   0.875    -.2750489    .2341313
      _Iyear_2004 |   .0106844   .1418433     0.08   0.940    -.2673235    .2886922
      _Iyear_2006 |   .0557946   .1305277     0.43   0.669    -.2000351    .3116242
      _Iyear_2007 |  -.0208323   .1433697    -0.15   0.884    -.3018318    .2601671
      _Iyear_2008 |    .092523   .1498551     0.62   0.537    -.2011877    .3862337
      _Iyear_2009 |   .0625923   .1503446     0.42   0.677    -.2320776    .3572622
      _Iyear_2010 |   .0766544   .1407654     0.54   0.586    -.1992407    .3525495
      _Iyear_2011 |   .2467677   .1323394     1.86   0.062    -.0126128    .5061481
      _Iyear_2012 |   .1196173   .1451203     0.82   0.410    -.1648132    .4040478
      _Iyear_2013 |   .0479902   .1518873     0.32   0.752    -.2497034    .3456837
      _Iyear_2015 |   .0906473   .1333301     0.68   0.497     -.170675    .3519695
            _cons |    -.66525   .2295037    -2.90   0.004    -1.115069   -.2154309
-----------------+----------------------------------------------------------------
          sigma_u |          0
          sigma_e |  .13277651
              rho |          0   (fraction of variance due to u_i)
------------------------------------------------------------------------------

. eststo yearfe

.
. * Baseline + two-way FE
. xi: xtreg lr_eco_broad wdi_export_l1 wdi_import_l1 po_mean_l1 ///
>          wdi_gdpgr_l1 wdi_gdpcapcur_l1 lr_eco_broad_l1 i.countryname i.year
i.countryname     _Icountryna_1-15    (_Icountryna_1 for cou~e==Austria omitted)
i.year            _Iyear_1976-2015    (naturally coded; _Iyear_1976 omitted)

Random-effects GLS regression                   Number of obs     =        129
Group variable: panelvar_id                     Number of groups  =         15

R-sq:                                           Obs per group:
     within  = 0.6156                                         min =          2
     between = 1.0000                                         avg =        8.6
     overall = 0.7017                                         max =         15
```

```
                                                Wald chi2(57)     =     167.01
corr(u_i, X)   = 0 (assumed)                    Prob > chi2       =     0.0000

------------------------------------------------------------------------------
    lr_eco_broad |      Coef.   Std. Err.      z    P>|z|     [95% Conf. Interval]
-----------------+------------------------------------------------------------
   wdi_export_l1 |   .0073806    .004368     1.69   0.091    -.0011805    .0159418
   wdi_import_l1 |  -.0005265   .0060646    -0.09   0.931    -.0124128    .0113599
      po_mean_l1 |   .0940595    .064808     1.45   0.147    -.0329618    .2210809
    wdi_gdpgr_l1 |  -.0112481    .008359    -1.35   0.178    -.0276314    .0051351
wdi_gdpcapcur_l1 |  -8.46e-06   3.70e-06    -2.29   0.022    -.0000157   -1.21e-06
 lr_eco_broad_l1 |   .3103849   .0914011     3.40   0.001      .131242    .4895278
   _Icountryna_2 |   -.032812   .1279531    -0.26   0.798    -.2835955    .2179715
   _Icountryna_3 |  -.1478304   .1547228    -0.96   0.339    -.4510816    .1554207
   _Icountryna_4 |   .2006005   .0971654     2.06   0.039     .0101598    .3910411
   _Icountryna_5 |   .2184204   .1181751     1.85   0.065    -.0131985    .4500394
   _Icountryna_6 |    .244299   .1002588     2.44   0.015     .0477953    .4408026
   _Icountryna_7 |   .1895331   .0983177     1.93   0.054    -.0031659    .3822322
   _Icountryna_8 |  -.0680664   .1404109    -0.48   0.628    -.3432668    .2071339
   _Icountryna_9 |   .3365722   .1029007     3.27   0.001     .1348905    .5382538
  _Icountryna_10 |  -.2539053   .1907165    -1.33   0.183    -.6277028    .1198921
  _Icountryna_11 |  -.0370404   .1020219    -0.36   0.717    -.2369997    .1629188
  _Icountryna_12 |   .1766941   .1081574     1.63   0.102    -.0352906    .3886788
  _Icountryna_13 |   .1713868    .105032     1.63   0.103    -.0344722    .3772458
  _Icountryna_14 |   .0960062   .1123821     0.85   0.393    -.1242586     .316271
  _Icountryna_15 |   .2632888   .1036532     2.54   0.011     .0601323    .4664453
     _Iyear_1977 |    .216985   .1321683     1.64   0.101    -.0420602    .4760302
     _Iyear_1978 |   .3579384   .1787633     2.00   0.045     .0075688    .7083079
     _Iyear_1979 |   .1819841   .1272024     1.43   0.153     -.067328    .4312962
     _Iyear_1980 |   .0932479   .1751283     0.53   0.594    -.2499972     .436493
     _Iyear_1981 |    .338767   .1273848     2.66   0.008     .0890974    .5884366
     _Iyear_1982 |    .154474   .1441718     1.07   0.284    -.1280976    .4370455
     _Iyear_1983 |   .1488775   .1254534     1.19   0.235    -.0970067    .3947618
     _Iyear_1984 |   .0345654   .1509159     0.23   0.819    -.2612243    .3303552
     _Iyear_1985 |   .2772785   .1494952     1.85   0.064    -.0157267    .5702837
     _Iyear_1986 |   .1558606   .1478208     1.05   0.292    -.1338629     .445584
     _Iyear_1987 |   .0922322   .1192787     0.77   0.439    -.1415498    .3260141
     _Iyear_1988 |   .3945655   .1761665     2.24   0.025     .0492855    .7398454
     _Iyear_1989 |   .1671566   .1332088     1.25   0.210    -.0939279     .428241
     _Iyear_1990 |  -.0041393    .135481    -0.03   0.976    -.2696772    .2613986
     _Iyear_1991 |   .0109236   .1552489     0.07   0.944    -.2933587    .3152058
     _Iyear_1992 |    .221421   .1400134     1.58   0.114    -.0530001    .4958422
     _Iyear_1993 |   .2684667   .1567707     1.71   0.087    -.0387983    .5757317
     _Iyear_1994 |   .3385326   .1335312     2.54   0.011     .0768162     .600249
     _Iyear_1995 |    .197817   .1475919     1.34   0.180    -.0914578    .4870918
     _Iyear_1996 |   .2654508   .1448174     1.83   0.067    -.0183861    .5492876
     _Iyear_1997 |   .2030793   .1598156     1.27   0.204    -.1101534    .5163121
     _Iyear_1998 |   .3099653   .1412848     2.19   0.028     .0330523    .5868784
     _Iyear_1999 |   .0705616   .1424586     0.50   0.620    -.2086521    .3497753
     _Iyear_2000 |     .07561   .1577484     0.48   0.632    -.2335713    .3847912
     _Iyear_2001 |   .0652095   .1412347     0.46   0.644    -.2116055    .3420245
     _Iyear_2002 |   .1189928   .1265424     0.94   0.347    -.1290258    .3670113
     _Iyear_2003 |   .0821639   .1460489     0.56   0.574    -.2040866    .3684145
     _Iyear_2004 |    .108049   .1513308     0.71   0.475     -.188554     .404652
     _Iyear_2006 |   .2544551   .1498215     1.70   0.089    -.0391897       .5481
     _Iyear_2007 |   .0927859   .1623509     0.57   0.568     -.225416    .4109878
     _Iyear_2008 |   .2646209   .1656309     1.60   0.110    -.0600096    .5892515
     _Iyear_2009 |   .1550864   .1678879     0.92   0.356    -.1739679    .4841407
     _Iyear_2010 |   .3016684   .1720046     1.75   0.079    -.0354545    .6387913
     _Iyear_2011 |    .397884   .1591364     2.50   0.012     .0859823    .7097857
     _Iyear_2012 |   .2952563   .1710685     1.73   0.084    -.0400318    .6305444
     _Iyear_2013 |   .0985677   .1625189     0.61   0.544    -.2199635     .417099
     _Iyear_2015 |   .2177608   .1558904     1.40   0.162    -.0877788    .5233003
           _cons |  -1.210207   .4029104    -3.00   0.003    -1.999897    -.420517
-----------------+------------------------------------------------------------
         sigma_u |          0
         sigma_e |  .13277651
             rho |          0   (fraction of variance due to u_i)
------------------------------------------------------------------------------
```

```
. eststo twowayfe

.
. * Regression table for fixed effects
. * S4 Table
. esttab countryfe yearfe twowayfe using ///
>         fe_table.rtf, replace keep(wdi_import_l1 wdi_export_l1 po_mean_l1 ///
>         wdi_gdpgr_l1 wdi_gdpcapcur_l1 lr_eco_broad_l1 _cons) ///
>         cells(b(star fmt(%9.4f)) se(par)) stats(r2_o N, fmt(2 0)) varwidth(10) ///
>         modelwidth(9) order(wdi_import_l1 wdi_export_l1 po_mean_l1 ///
>         wdi_gdpgr_l1 wdi_gdpcapcur_l1 lr_eco_broad_l1 _cons) ///
>         varlabels(wdi_import_l1 "Imports" wdi_export_l1 "Exports" ///
>         po_mean_l1 "Median voter" wdi_gdpgr_l1 "GDP growth" ///
>         wdi_gdpcapcur_l1 "GDP/capita" lr_eco_broad_l1 "Lagged DV" _cons "Constant")
> ///
>         mlabels("country FE" "year FE" "two-way") ///
>         addnote("Standard errors in parentheses; two-sided tests; p < .05 *; p < .01
>  **; p < .001 ***
> ") ///
>         nonumbers nolines
(note: file fe_table.rtf not found)
(output written to fe_table.rtf)

.
. log close
      name:  <unnamed>
       log:  C:\data\sync\global\3 - PLOS\revision 2\OSF\center_of_gravity.log
  log type:  text
 closed on:   6 Feb 2019, 10:01:20
-------------------------------------------------------------------------------------
> -----------------
```
